# Searches for physics beyond the standard model with the $M_{\mathrm {T2}}$ variable in hadronic final states with and without disappearing tracks in proton–proton collisions at $\sqrt{s}=13\,\text {Te}\text {V}$

**DOI:** 10.1140/epjc/s10052-019-7493-x

**Published:** 2020-01-03

**Authors:** A. M. Sirunyan, A. Tumasyan, W. Adam, F. Ambrogi, T. Bergauer, J. Brandstetter, M. Dragicevic, J. Erö, A. Escalante Del Valle, M. Flechl, R. Frühwirth, M. Jeitler, N. Krammer, I. Krätschmer, D. Liko, T. Madlener, I. Mikulec, N. Rad, J. Schieck, R. Schöfbeck, M. Spanring, D. Spitzbart, W. Waltenberger, C.-E. Wulz, M. Zarucki, V. Drugakov, V. Mossolov, J. Suarez Gonzalez, M. R. Darwish, E. A. De Wolf, D. Di Croce, X. Janssen, A. Lelek, M. Pieters, H. Rejeb Sfar, H. Van Haevermaet, P. Van Mechelen, S. Van Putte, N. Van Remortel, F. Blekman, E. S. Bols, S. S. Chhibra, J. D’Hondt, J. De Clercq, D. Lontkovskyi, S. Lowette, I. Marchesini, S. Moortgat, Q. Python, K. Skovpen, S. Tavernier, W. Van Doninck, P. Van Mulders, D. Beghin, B. Bilin, H. Brun, B. Clerbaux, G. De Lentdecker, H. Delannoy, B. Dorney, L. Favart, A. Grebenyuk, A. K. Kalsi, A. Popov, N. Postiau, E. Starling, L. Thomas, C. Vander Velde, P. Vanlaer, D. Vannerom, T. Cornelis, D. Dobur, I. Khvastunov, M. Niedziela, C. Roskas, D. Trocino, M. Tytgat, W. Verbeke, B. Vermassen, M. Vit, O. Bondu, G. Bruno, C. Caputo, P. David, C. Delaere, M. Delcourt, A. Giammanco, V. Lemaitre, J. Prisciandaro, A. Saggio, M. Vidal Marono, P. Vischia, J. Zobec, F. L. Alves, G. A. Alves, G. Correia Silva, C. Hensel, A. Moraes, P. Rebello Teles, E. Belchior Batista Das Chagas, W. Carvalho, J. Chinellato, E. Coelho, E. M. Da Costa, G. G. Da Silveira, D. De Jesus Damiao, C. De Oliveira Martins, S. Fonseca De Souza, L. M. Huertas Guativa, H. Malbouisson, J. Martins, D. Matos Figueiredo, M. Medina Jaime, M. Melo De Almeida, C. Mora Herrera, L. Mundim, H. Nogima, W. L. Prado Da Silva, L. J. Sanchez Rosas, A. Santoro, A. Sznajder, M. Thiel, E. J. Tonelli Manganote, F. Torres Da Silva De Araujo, A. Vilela Pereira, C. A. Bernardes, L. Calligaris, T. R. Fernandez Perez Tomei, E. M. Gregores, D. S. Lemos, P. G. Mercadante, S. F. Novaes, SandraS. Padula, A. Aleksandrov, G. Antchev, R. Hadjiiska, P. Iaydjiev, M. Misheva, M. Rodozov, M. Shopova, G. Sultanov, M. Bonchev, A. Dimitrov, T. Ivanov, L. Litov, B. Pavlov, P. Petkov, W. Fang, X. Gao, L. Yuan, G. M. Chen, H. S. Chen, M. Chen, C. H. Jiang, D. Leggat, H. Liao, Z. Liu, A. Spiezia, J. Tao, E. Yazgan, H. Zhang, S. Zhang, J. Zhao, A. Agapitos, Y. Ban, G. Chen, A. Levin, J. Li, L. Li, Q. Li, Y. Mao, S. J. Qian, D. Wang, Q. Wang, M. Ahmad, Z. Hu, Y. Wang, M. Xiao, C. Avila, A. Cabrera, C. Florez, C. F. González Hernández, M. A. Segura Delgado, J. Mejia Guisao, J. D. Ruiz Alvarez, C. A. Salazar González, N. Vanegas Arbelaez, D. Giljanović, N. Godinovic, D. Lelas, I. Puljak, T. Sculac, Z. Antunovic, M. Kovac, V. Brigljevic, D. Ferencek, K. Kadija, B. Mesic, M. Roguljic, A. Starodumov, T. Susa, M. W. Ather, A. Attikis, E. Erodotou, A. Ioannou, M. Kolosova, S. Konstantinou, G. Mavromanolakis, J. Mousa, C. Nicolaou, F. Ptochos, P. A. Razis, H. Rykaczewski, D. Tsiakkouri, M. Finger, M. Finger, A. Kveton, J. Tomsa, E. Ayala, E. Carrera Jarrin, Y. Assran, S. Elgammal, S. Bhowmik, A. Carvalho Antunes De Oliveira, R. K. Dewanjee, K. Ehataht, M. Kadastik, M. Raidal, C. Veelken, P. Eerola, L. Forthomme, H. Kirschenmann, K. Osterberg, M. Voutilainen, F. Garcia, J. Havukainen, J. K. Heikkilä, V. Karimäki, M. S. Kim, R. Kinnunen, T. Lampén, K. Lassila-Perini, S. Laurila, S. Lehti, T. Lindén, P. Luukka, T. Mäenpää, H. Siikonen, E. Tuominen, J. Tuominiemi, T. Tuuva, M. Besancon, F. Couderc, M. Dejardin, D. Denegri, B. Fabbro, J. L. Faure, F. Ferri, S. Ganjour, A. Givernaud, P. Gras, G. Hamel de Monchenault, P. Jarry, C. Leloup, E. Locci, J. Malcles, J. Rander, A. Rosowsky, M. Ö. Sahin, A. Savoy-Navarro, M. Titov, S. Ahuja, C. Amendola, F. Beaudette, P. Busson, C. Charlot, B. Diab, G. Falmagne, R. Granier de Cassagnac, I. Kucher, A. Lobanov, C. Martin Perez, M. Nguyen, C. Ochando, P. Paganini, J. Rembser, R. Salerno, J. B. Sauvan, Y. Sirois, A. Zabi, A. Zghiche, J.-L. Agram, J. Andrea, D. Bloch, G. Bourgatte, J.-M. Brom, E. C. Chabert, C. Collard, E. Conte, J.-C. Fontaine, D. Gelé, U. Goerlach, M. Jansová, A.-C. Le Bihan, N. Tonon, P. Van Hove, S. Gadrat, S. Beauceron, C. Bernet, G. Boudoul, C. Camen, A. Carle, N. Chanon, R. Chierici, D. Contardo, P. Depasse, H. El Mamouni, J. Fay, S. Gascon, M. Gouzevitch, B. Ille, Sa. Jain, F. Lagarde, I. B. Laktineh, H. Lattaud, A. Lesauvage, M. Lethuillier, L. Mirabito, S. Perries, V. Sordini, L. Torterotot, G. Touquet, M. Vander Donckt, S. Viret, A. Khvedelidze, Z. Tsamalaidze, C. Autermann, L. Feld, M. K. Kiesel, K. Klein, M. Lipinski, D. Meuser, A. Pauls, M. Preuten, M. P. Rauch, J. Schulz, M. Teroerde, B. Wittmer, M. Erdmann, B. Fischer, S. Ghosh, T. Hebbeker, K. Hoepfner, H. Keller, L. Mastrolorenzo, M. Merschmeyer, A. Meyer, P. Millet, G. Mocellin, S. Mondal, S. Mukherjee, D. Noll, A. Novak, T. Pook, A. Pozdnyakov, T. Quast, M. Radziej, Y. Rath, H. Reithler, J. Roemer, A. Schmidt, S. C. Schuler, A. Sharma, S. Wiedenbeck, S. Zaleski, G. Flügge, W. Haj Ahmad, O. Hlushchenko, T. Kress, T. Müller, A. Nowack, C. Pistone, O. Pooth, D. Roy, H. Sert, A. Stahl, M. Aldaya Martin, P. Asmuss, I. Babounikau, H. Bakhshiansohi, K. Beernaert, O. Behnke, A. Bermúdez Martínez, D. Bertsche, A. A. Bin Anuar, K. Borras, V. Botta, A. Campbell, A. Cardini, P. Connor, S. Consuegra Rodríguez, C. Contreras-Campana, V. Danilov, A. De Wit, M. M. Defranchis, C. Diez Pardos, D. Domínguez Damiani, G. Eckerlin, D. Eckstein, T. Eichhorn, A. Elwood, E. Eren, E. Gallo, A. Geiser, A. Grohsjean, M. Guthoff, M. Haranko, A. Harb, A. Jafari, N. Z. Jomhari, H. Jung, A. Kasem, M. Kasemann, H. Kaveh, J. Keaveney, C. Kleinwort, J. Knolle, D. Krücker, W. Lange, T. Lenz, J. Lidrych, K. Lipka, W. Lohmann, R. Mankel, I.-A. Melzer-Pellmann, A. B. Meyer, M. Meyer, M. Missiroli, G. Mittag, J. Mnich, A. Mussgiller, V. Myronenko, D. Pérez Adán, S. K. Pflitsch, D. Pitzl, A. Raspereza, A. Saibel, M. Savitskyi, V. Scheurer, P. Schütze, C. Schwanenberger, R. Shevchenko, A. Singh, H. Tholen, O. Turkot, A. Vagnerini, M. Van De Klundert, R. Walsh, Y. Wen, K. Wichmann, C. Wissing, O. Zenaiev, R. Zlebcik, R. Aggleton, S. Bein, L. Benato, A. Benecke, V. Blobel, T. Dreyer, A. Ebrahimi, F. Feindt, A. Fröhlich, C. Garbers, E. Garutti, D. Gonzalez, P. Gunnellini, J. Haller, A. Hinzmann, A. Karavdina, G. Kasieczka, R. Klanner, R. Kogler, N. Kovalchuk, S. Kurz, V. Kutzner, J. Lange, T. Lange, A. Malara, J. Multhaup, C. E. N. Niemeyer, A. Perieanu, A. Reimers, O. Rieger, C. Scharf, P. Schleper, S. Schumann, J. Schwandt, J. Sonneveld, H. Stadie, G. Steinbrück, F. M. Stober, B. Vormwald, I. Zoi, M. Akbiyik, C. Barth, M. Baselga, S. Baur, T. Berger, E. Butz, R. Caspart, T. Chwalek, W. De Boer, A. Dierlamm, K. El Morabit, N. Faltermann, M. Giffels, P. Goldenzweig, A. Gottmann, M. A. Harrendorf, F. Hartmann, U. Husemann, I. Katkov, S. Kudella, S. Mitra, M. U. Mozer, D. Müller, Th. Müller, M. Musich, A. Nürnberg, G. Quast, K. Rabbertz, M. Schröder, I. Shvetsov, H. J. Simonis, R. Ulrich, M. Wassmer, M. Weber, C. Wöhrmann, R. Wolf, G. Anagnostou, P. Asenov, G. Daskalakis, T. Geralis, A. Kyriakis, D. Loukas, G. Paspalaki, M. Diamantopoulou, G. Karathanasis, P. Kontaxakis, A. Manousakis-katsikakis, A. Panagiotou, I. Papavergou, N. Saoulidou, A. Stakia, K. Theofilatos, K. Vellidis, E. Vourliotis, G. Bakas, K. Kousouris, I. Papakrivopoulos, G. Tsipolitis, I. Evangelou, C. Foudas, P. Gianneios, P. Katsoulis, P. Kokkas, S. Mallios, K. Manitara, N. Manthos, I. Papadopoulos, J. Strologas, F. A. Triantis, D. Tsitsonis, M. Bartók, R. Chudasama, M. Csanad, P. Major, K. Mandal, A. Mehta, M. I. Nagy, G. Pasztor, O. Surányi, G. I. Veres, G. Bencze, C. Hajdu, D. Horvath, F. Sikler, T. Vámi, V. Veszpremi, G. Vesztergombi, N. Beni, S. Czellar, J. Karancsi, A. Makovec, J. Molnar, Z. Szillasi, P. Raics, D. Teyssier, Z. L. Trocsanyi, B. Ujvari, T. Csorgo, W. J. Metzger, F. Nemes, T. Novak, S. Choudhury, J. R. Komaragiri, P. C. Tiwari, S. Bahinipati, C. Kar, G. Kole, P. Mal, V. K. Muraleedharan Nair Bindhu, A. Nayak, D. K. Sahoo, S. K. Swain, S. Bansal, S. B. Beri, V. Bhatnagar, S. Chauhan, R. Chawla, N. Dhingra, R. Gupta, A. Kaur, M. Kaur, S. Kaur, P. Kumari, M. Lohan, M. Meena, K. Sandeep, S. Sharma, J. B. Singh, A. K. Virdi, G. Walia, A. Bhardwaj, B. C. Choudhary, R. B. Garg, M. Gola, S. Keshri, Ashok Kumar, M. Naimuddin, P. Priyanka, K. Ranjan, Aashaq Shah, R. Sharma, R. Bhardwaj, M. Bharti, R. Bhattacharya, S. Bhattacharya, U. Bhawandeep, D. Bhowmik, S. Dutta, S. Ghosh, M. Maity, K. Mondal, S. Nandan, A. Purohit, P. K. Rout, G. Saha, S. Sarkar, T. Sarkar, M. Sharan, B. Singh, S. Thakur, P. K. Behera, P. Kalbhor, A. Muhammad, P. R. Pujahari, A. Sharma, A. K. Sikdar, D. Dutta, V. Jha, V. Kumar, D. K. Mishra, P. K. Netrakanti, L. M. Pant, P. Shukla, T. Aziz, M. A. Bhat, S. Dugad, G. B. Mohanty, N. Sur, RavindraKumar Verma, S. Banerjee, S. Bhattacharya, S. Chatterjee, P. Das, M. Guchait, S. Karmakar, S. Kumar, G. Majumder, K. Mazumdar, N. Sahoo, S. Sawant, S. Dube, V. Hegde, B. Kansal, A. Kapoor, K. Kothekar, S. Pandey, A. Rane, A. Rastogi, S. Sharma, S. Chenarani, E. Eskandari Tadavani, S. M. Etesami, M. Khakzad, M. Mohammadi Najafabadi, M. Naseri, F. Rezaei Hosseinabadi, M. Felcini, M. Grunewald, M. Abbrescia, R. Aly, C. Calabria, A. Colaleo, D. Creanza, L. Cristella, N. De Filippis, M. De Palma, A. Di Florio, W. Elmetenawee, L. Fiore, A. Gelmi, G. Iaselli, M. Ince, S. Lezki, G. Maggi, M. Maggi, G. Miniello, S. My, S. Nuzzo, A. Pompili, G. Pugliese, R. Radogna, A. Ranieri, G. Selvaggi, L. Silvestris, F. M. Simone, R. Venditti, P. Verwilligen, G. Abbiendi, C. Battilana, D. Bonacorsi, L. Borgonovi, S. Braibant-Giacomelli, R. Campanini, P. Capiluppi, A. Castro, F. R. Cavallo, C. Ciocca, G. Codispoti, M. Cuffiani, G. M. Dallavalle, F. Fabbri, A. Fanfani, E. Fontanesi, P. Giacomelli, C. Grandi, L. Guiducci, F. Iemmi, S. Lo Meo, S. Marcellini, G. Masetti, F. L. Navarria, A. Perrotta, F. Primavera, A. M. Rossi, T. Rovelli, G. P. Siroli, N. Tosi, S. Albergo, S. Costa, A. Di Mattia, R. Potenza, A. Tricomi, C. Tuve, G. Barbagli, A. Cassese, R. Ceccarelli, V. Ciulli, C. Civinini, R. D’Alessandro, E. Focardi, G. Latino, P. Lenzi, M. Meschini, S. Paoletti, G. Sguazzoni, L. Viliani, L. Benussi, S. Bianco, D. Piccolo, M. Bozzo, F. Ferro, R. Mulargia, E. Robutti, S. Tosi, A. Benaglia, A. Beschi, F. Brivio, V. Ciriolo, S. Di Guida, M. E. Dinardo, P. Dini, S. Gennai, A. Ghezzi, P. Govoni, L. Guzzi, M. Malberti, S. Malvezzi, D. Menasce, F. Monti, L. Moroni, M. Paganoni, D. Pedrini, S. Ragazzi, T. Tabarelli de Fatis, D. Zuolo, S. Buontempo, N. Cavallo, A. De Iorio, A. Di Crescenzo, F. Fabozzi, F. Fienga, G. Galati, A. O. M. Iorio, L. Lista, S. Meola, P. Paolucci, B. Rossi, C. Sciacca, E. Voevodina, P. Azzi, N. Bacchetta, D. Bisello, A. Boletti, A. Bragagnolo, R. Carlin, P. Checchia, P. De Castro Manzano, T. Dorigo, U. Dosselli, F. Gasparini, U. Gasparini, A. Gozzelino, S. Y. Hoh, P. Lujan, M. Margoni, A. T. Meneguzzo, J. Pazzini, M. Presilla, P. Ronchese, R. Rossin, F. Simonetto, A. Tiko, M. Tosi, M. Zanetti, P. Zotto, G. Zumerle, A. Braghieri, D. Fiorina, P. Montagna, S. P. Ratti, V. Re, M. Ressegotti, C. Riccardi, P. Salvini, I. Vai, P. Vitulo, M. Biasini, G. M. Bilei, D. Ciangottini, L. Fanò, P. Lariccia, R. Leonardi, E. Manoni, G. Mantovani, V. Mariani, M. Menichelli, A. Rossi, A. Santocchia, D. Spiga, K. Androsov, P. Azzurri, G. Bagliesi, V. Bertacchi, L. Bianchini, T. Boccali, R. Castaldi, M. A. Ciocci, R. Dell’Orso, G. Fedi, L. Giannini, A. Giassi, M. T. Grippo, F. Ligabue, E. Manca, G. Mandorli, A. Messineo, F. Palla, A. Rizzi, G. Rolandi, S. Roy Chowdhury, A. Scribano, P. Spagnolo, R. Tenchini, G. Tonelli, N. Turini, A. Venturi, P. G. Verdini, F. Cavallari, M. Cipriani, D. Del Re, E. Di Marco, M. Diemoz, E. Longo, P. Meridiani, G. Organtini, F. Pandolfi, R. Paramatti, C. Quaranta, S. Rahatlou, C. Rovelli, F. Santanastasio, L. Soffi, N. Amapane, R. Arcidiacono, S. Argiro, M. Arneodo, N. Bartosik, R. Bellan, A. Bellora, C. Biino, A. Cappati, N. Cartiglia, S. Cometti, M. Costa, R. Covarelli, N. Demaria, B. Kiani, C. Mariotti, S. Maselli, E. Migliore, V. Monaco, E. Monteil, M. Monteno, M. M. Obertino, G. Ortona, L. Pacher, N. Pastrone, M. Pelliccioni, G. L. Pinna Angioni, A. Romero, M. Ruspa, R. Salvatico, V. Sola, A. Solano, D. Soldi, A. Staiano, S. Belforte, V. Candelise, M. Casarsa, F. Cossutti, A. Da Rold, G. Della Ricca, F. Vazzoler, A. Zanetti, B. Kim, D. H. Kim, G. N. Kim, J. Lee, S. W. Lee, C. S. Moon, Y. D. Oh, S. I. Pak, S. Sekmen, D. C. Son, Y. C. Yang, H. Kim, D. H. Moon, G. Oh, B. Francois, T. J. Kim, J. Park, S. Cho, S. Choi, Y. Go, S. Ha, B. Hong, K. Lee, K. S. Lee, J. Lim, J. Park, S. K. Park, Y. Roh, J. Yoo, J. Goh, H. S. Kim, J. Almond, J. H. Bhyun, J. Choi, S. Jeon, J. Kim, J. S. Kim, H. Lee, K. Lee, S. Lee, K. Nam, M. Oh, S. B. Oh, B. C. Radburn-Smith, U. K. Yang, H. D. Yoo, I. Yoon, G. B. Yu, D. Jeon, H. Kim, J. H. Kim, J. S. H. Lee, I. C. Park, I. J Watson, Y. Choi, C. Hwang, Y. Jeong, J. Lee, Y. Lee, I. Yu, V. Veckalns, V. Dudenas, A. Juodagalvis, A. Rinkevicius, G. Tamulaitis, J. Vaitkus, Z. A. Ibrahim, F. Mohamad Idris, W. A. T. Wan Abdullah, M. N. Yusli, Z. Zolkapli, J. F. Benitez, A. Castaneda Hernandez, J. A. Murillo Quijada, L. Valencia Palomo, H. Castilla-Valdez, E. De La Cruz-Burelo, I. Heredia-De La Cruz, R. Lopez-Fernandez, A. Sanchez-Hernandez, S. Carrillo Moreno, C. Oropeza Barrera, M. Ramirez-Garcia, F. Vazquez Valencia, J. Eysermans, I. Pedraza, H. A. Salazar Ibarguen, C. Uribe Estrada, A. Morelos Pineda, J. Mijuskovic, N. Raicevic, D. Krofcheck, S. Bheesette, P. H. Butler, A. Ahmad, M. Ahmad, Q. Hassan, H. R. Hoorani, W. A. Khan, M. A. Shah, M. Shoaib, M. Waqas, V. Avati, L. Grzanka, M. Malawski, H. Bialkowska, M. Bluj, B. Boimska, M. Górski, M. Kazana, M. Szleper, P. Zalewski, K. Bunkowski, A. Byszuk, K. Doroba, A. Kalinowski, M. Konecki, J. Krolikowski, M. Misiura, M. Olszewski, M. Walczak, M. Araujo, P. Bargassa, D. Bastos, A. Di Francesco, P. Faccioli, B. Galinhas, M. Gallinaro, J. Hollar, N. Leonardo, T. Niknejad, J. Seixas, K. Shchelina, G. Strong, O. Toldaiev, J. Varela, S. Afanasiev, P. Bunin, M. Gavrilenko, I. Golutvin, I. Gorbunov, A. Kamenev, V. Karjavine, A. Lanev, A. Malakhov, V. Matveev, P. Moisenz, V. Palichik, V. Perelygin, M. Savina, S. Shmatov, S. Shulha, N. Skatchkov, V. Smirnov, N. Voytishin, A. Zarubin, L. Chtchipounov, V. Golovtcov, Y. Ivanov, V. Kim, E. Kuznetsova, P. Levchenko, V. Murzin, V. Oreshkin, I. Smirnov, D. Sosnov, V. Sulimov, L. Uvarov, A. Vorobyev, Yu. Andreev, A. Dermenev, S. Gninenko, N. Golubev, A. Karneyeu, M. Kirsanov, N. Krasnikov, A. Pashenkov, D. Tlisov, A. Toropin, V. Epshteyn, V. Gavrilov, N. Lychkovskaya, A. Nikitenko, V. Popov, I. Pozdnyakov, G. Safronov, A. Spiridonov, A. Stepennov, M. Toms, E. Vlasov, A. Zhokin, T. Aushev, O. Bychkova, R. Chistov, M. Danilov, S. Polikarpov, E. Tarkovskii, V. Andreev, M. Azarkin, I. Dremin, M. Kirakosyan, A. Terkulov, A. Belyaev, E. Boos, V. Bunichev, M. Dubinin, L. Dudko, A. Ershov, A. Gribushin, V. Klyukhin, O. Kodolova, I. Lokhtin, S. Obraztsov, S. Petrushanko, V. Savrin, A. Barnyakov, V. Blinov, T. Dimova, L. Kardapoltsev, Y. Skovpen, I. Azhgirey, I. Bayshev, S. Bitioukov, V. Kachanov, D. Konstantinov, P. Mandrik, V. Petrov, R. Ryutin, S. Slabospitskii, A. Sobol, S. Troshin, N. Tyurin, A. Uzunian, A. Volkov, A. Babaev, A. Iuzhakov, V. Okhotnikov, V. Borchsh, V. Ivanchenko, E. Tcherniaev, P. Adzic, P. Cirkovic, M. Dordevic, P. Milenovic, J. Milosevic, M. Stojanovic, M. Aguilar-Benitez, J. Alcaraz Maestre, A. lvarez Fernández, I. Bachiller, M. Barrio Luna, J. A. Brochero Cifuentes, C. A. Carrillo Montoya, M. Cepeda, M. Cerrada, N. Colino, B. De La Cruz, A. Delgado Peris, C. Fernandez Bedoya, J. P. Fernández Ramos, J. Flix, M. C. Fouz, O. Gonzalez Lopez, S. Goy Lopez, J. M. Hernandez, M. I. Josa, D. Moran, Navarro Tobar, A. Pérez-Calero Yzquierdo, J. Puerta Pelayo, I. Redondo, L. Romero, S. Sánchez Navas, M. S. Soares, A. Triossi, C. Willmott, C. Albajar, J. F. de Trocóniz, R. Reyes-Almanza, B. Alvarez Gonzalez, J. Cuevas, C. Erice, J. Fernandez Menendez, S. Folgueras, I. Gonzalez Caballero, J. R. González Fernández, E. Palencia Cortezon, V. Rodríguez Bouza, S. Sanchez Cruz, I. J. Cabrillo, A. Calderon, B. Chazin Quero, J. Duarte Campderros, M. Fernandez, P. J. Fernández Manteca, A. García Alonso, G. Gomez, C. Martinez Rivero, P. Martinez Ruiz del Arbol, F. Matorras, J. Piedra Gomez, C. Prieels, T. Rodrigo, A. Ruiz-Jimeno, L. Russo, L. Scodellaro, I. Vila, J. M. Vizan Garcia, K. Malagalage, W. G. D. Dharmaratna, N. Wickramage, D. Abbaneo, B. Akgun, E. Auffray, G. Auzinger, J. Baechler, P. Baillon, A. H. Ball, D. Barney, J. Bendavid, M. Bianco, A. Bocci, P. Bortignon, E. Bossini, C. Botta, E. Brondolin, T. Camporesi, A. Caratelli, G. Cerminara, E. Chapon, G. Cucciati, D. d’Enterria, A. Dabrowski, N. Daci, V. Daponte, A. David, O. Davignon, A. De Roeck, M. Deile, M. Dobson, M. Dünser, N. Dupont, A. Elliott-Peisert, N. Emriskova, F. Fallavollita, D. Fasanella, S. Fiorendi, G. Franzoni, J. Fulcher, W. Funk, S. Giani, D. Gigi, A. Gilbert, K. Gill, F. Glege, L. Gouskos, M. Gruchala, M. Guilbaud, D. Gulhan, J. Hegeman, C. Heidegger, Y. Iiyama, V. Innocente, T. James, P. Janot, O. Karacheban, J. Kaspar, J. Kieseler, M. Krammer, N. Kratochwil, C. Lange, P. Lecoq, C. Lourenço, L. Malgeri, M. Mannelli, A. Massironi, F. Meijers, J. A. Merlin, S. Mersi, E. Meschi, F. Moortgat, M. Mulders, J. Ngadiuba, J. Niedziela, S. Nourbakhsh, S. Orfanelli, L. Orsini, F. Pantaleo, L. Pape, E. Perez, M. Peruzzi, A. Petrilli, G. Petrucciani, A. Pfeiffer, M. Pierini, F. M. Pitters, D. Rabady, A. Racz, M. Rieger, M. Rovere, H. Sakulin, C. Schäfer, C. Schwick, M. Selvaggi, A. Sharma, P. Silva, W. Snoeys, P. Sphicas, J. Steggemann, S. Summers, V. R. Tavolaro, D. Treille, A. Tsirou, G. P. Van Onsem, A. Vartak, M. Verzetti, W. D. Zeuner, L. Caminada, K. Deiters, W. Erdmann, R. Horisberger, Q. Ingram, H. C. Kaestli, D. Kotlinski, U. Langenegger, T. Rohe, S. A. Wiederkehr, M. Backhaus, P. Berger, N. Chernyavskaya, G. Dissertori, M. Dittmar, M. Donegà, C. Dorfer, T. A. Gómez Espinosa, C. Grab, D. Hits, T. Klijnsma, W. Lustermann, A.-M. Lyon, R. A. Manzoni, M. T. Meinhard, F. Micheli, P. Musella, F. Nessi-Tedaldi, F. Pauss, G. Perrin, L. Perrozzi, S. Pigazzini, M. G. Ratti, M. Reichmann, C. Reissel, T. Reitenspiess, D. Ruini, D. A. Sanz Becerra, M. Schönenberger, L. Shchutska, M. L. Vesterbacka Olsson, R. Wallny, D. H. Zhu, T. K. Aarrestad, C. Amsler, D. Brzhechko, M. F. Canelli, A. De Cosa, R. Del Burgo, S. Donato, B. Kilminster, S. Leontsinis, V. M. Mikuni, I. Neutelings, G. Rauco, P. Robmann, K. Schweiger, C. Seitz, Y. Takahashi, S. Wertz, A. Zucchetta, T. H. Doan, C. M. Kuo, W. Lin, A. Roy, S. S. Yu, P. Chang, Y. Chao, K. F. Chen, P. H. Chen, W.-S. Hou, Y. y. Li, R.-S. Lu, E. Paganis, A. Psallidas, A. Steen, B. Asavapibhop, C. Asawatangtrakuldee, N. Srimanobhas, N. Suwonjandee, A. Bat, F. Boran, A. Celik, S. Cerci, S. Damarseckin, Z. S. Demiroglu, F. Dolek, C. Dozen, I. Dumanoglu, G. Gokbulut, EmineGurpinar Guler, Y. Guler, I. Hos, C. Isik, E. E. Kangal, O. Kara, A. Kayis Topaksu, U. Kiminsu, G. Onengut, K. Ozdemir, S. Ozturk, A. E. Simsek, D. Sunar Cerci, U. G. Tok, S. Turkcapar, I. S. Zorbakir, C. Zorbilmez, B. Isildak, G. Karapinar, M. Yalvac, I. O. Atakisi, E. Gülmez, M. Kaya, O. Kaya, Ö. Özçelik, S. Tekten, E. A. Yetkin, A. Cakir, K. Cankocak, Y. Komurcu, S. Sen, B. Kaynak, S. Ozkorucuklu, B. Grynyov, L. Levchuk, E. Bhal, S. Bologna, J. J. Brooke, D. Burns, E. Clement, D. Cussans, H. Flacher, J. Goldstein, G. P. Heath, H. F. Heath, L. Kreczko, B. Krikler, S. Paramesvaran, B. Penning, T. Sakuma, S. Seif El Nasr-Storey, V. J. Smith, J. Taylor, A. Titterton, K. W. Bell, A. Belyaev, C. Brew, R. M. Brown, D. J. A. Cockerill, J. A. Coughlan, K. Harder, S. Harper, J. Linacre, K. Manolopoulos, D. M. Newbold, E. Olaiya, D. Petyt, T. Reis, T. Schuh, C. H. Shepherd-Themistocleous, A. Thea, I. R. Tomalin, T. Williams, W. J. Womersley, R. Bainbridge, P. Bloch, J. Borg, S. Breeze, O. Buchmuller, A. Bundock, GurpreetSingh CHAHAL, D. Colling, P. Dauncey, G. Davies, M. Della Negra, R. Di Maria, P. Everaerts, G. Hall, G. Iles, M. Komm, C. Laner, L. Lyons, A.-M. Magnan, S. Malik, A. Martelli, V. Milosevic, A. Morton, J. Nash, V. Palladino, M. Pesaresi, D. M. Raymond, A. Richards, A. Rose, E. Scott, C. Seez, A. Shtipliyski, M. Stoye, T. Strebler, A. Tapper, K. Uchida, T. Virdee, N. Wardle, D. Winterbottom, J. Wright, A. G. Zecchinelli, S. C. Zenz, J. E. Cole, P. R. Hobson, A. Khan, P. Kyberd, C. K. Mackay, I. D. Reid, L. Teodorescu, S. Zahid, K. Call, B. Caraway, J. Dittmann, K. Hatakeyama, C. Madrid, B. McMaster, N. Pastika, C. Smith, R. Bartek, A. Dominguez, R. Uniyal, A. M. Vargas Hernandez, A. Buccilli, S. I. Cooper, C. Henderson, P. Rumerio, C. West, A. Albert, D. Arcaro, Z. Demiragli, D. Gastler, C. Richardson, J. Rohlf, D. Sperka, I. Suarez, L. Sulak, D. Zou, G. Benelli, B. Burkle, X. Coubez, D. Cutts, Y. t. Duh, M. Hadley, U. Heintz, J. M. Hogan, K. H. M. Kwok, E. Laird, G. Landsberg, K. T. Lau, J. Lee, Z. Mao, M. Narain, S. Sagir, R. Syarif, E. Usai, D. Yu, W. Zhang, R. Band, C. Brainerd, R. Breedon, M. Calderon De La Barca Sanchez, M. Chertok, J. Conway, R. Conway, P. T. Cox, R. Erbacher, C. Flores, G. Funk, F. Jensen, W. Ko, O. Kukral, R. Lander, M. Mulhearn, D. Pellett, J. Pilot, M. Shi, D. Taylor, K. Tos, M. Tripathi, Z. Wang, F. Zhang, M. Bachtis, C. Bravo, R. Cousins, A. Dasgupta, A. Florent, J. Hauser, M. Ignatenko, N. Mccoll, W. A. Nash, S. Regnard, D. Saltzberg, C. Schnaible, B. Stone, V. Valuev, K. Burt, Y. Chen, R. Clare, J. W. Gary, S. M. A. Ghiasi Shirazi, G. Hanson, G. Karapostoli, E. Kennedy, O. R. Long, M. Olmedo Negrete, M. I. Paneva, W. Si, L. Wang, S. Wimpenny, B. R. Yates, Y. Zhang, J. G. Branson, P. Chang, S. Cittolin, S. Cooperstein, N. Deelen, M. Derdzinski, R. Gerosa, D. Gilbert, B. Hashemi, D. Klein, V. Krutelyov, J. Letts, M. Masciovecchio, S. May, S. Padhi, M. Pieri, V. Sharma, M. Tadel, F. Würthwein, A. Yagil, G. Zevi Della Porta, N. Amin, R. Bhandari, C. Campagnari, M. Citron, V. Dutta, M. Franco Sevilla, J. Incandela, B. Marsh, H. Mei, A. Ovcharova, H. Qu, J. Richman, U. Sarica, D. Stuart, S. Wang, D. Anderson, A. Bornheim, O. Cerri, I. Dutta, J. M. Lawhorn, N. Lu, J. Mao, H. B. Newman, T. Q. Nguyen, J. Pata, M. Spiropulu, J. R. Vlimant, S. Xie, Z. Zhang, R. Y. Zhu, M. B. Andrews, T. Ferguson, T. Mudholkar, M. Paulini, M. Sun, I. Vorobiev, M. Weinberg, J. P. Cumalat, W. T. Ford, E. MacDonald, T. Mulholland, R. Patel, A. Perloff, K. Stenson, K. A. Ulmer, S. R. Wagner, J. Alexander, Y. Cheng, J. Chu, A. Datta, A. Frankenthal, K. Mcdermott, J. R. Patterson, D. Quach, A. Ryd, S. M. Tan, Z. Tao, J. Thom, P. Wittich, M. Zientek, S. Abdullin, M. Albrow, M. Alyari, G. Apollinari, A. Apresyan, A. Apyan, S. Banerjee, L. A. T. Bauerdick, A. Beretvas, D. Berry, J. Berryhill, P. C. Bhat, K. Burkett, J. N. Butler, A. Canepa, G. B. Cerati, H. W. K. Cheung, F. Chlebana, M. Cremonesi, J. Duarte, V. D. Elvira, J. Freeman, Z. Gecse, E. Gottschalk, L. Gray, D. Green, S. Grünendahl, O. Gutsche, AllisonReinsvold Hall, J. Hanlon, R. M. Harris, S. Hasegawa, R. Heller, J. Hirschauer, B. Jayatilaka, S. Jindariani, M. Johnson, U. Joshi, B. Klima, M. J. Kortelainen, B. Kreis, S. Lammel, J. Lewis, D. Lincoln, R. Lipton, M. Liu, T. Liu, J. Lykken, K. Maeshima, J. M. Marraffino, D. Mason, P. McBride, P. Merkel, S. Mrenna, S. Nahn, V. O’Dell, V. Papadimitriou, K. Pedro, C. Pena, G. Rakness, F. Ravera, L. Ristori, B. Schneider, E. Sexton-Kennedy, N. Smith, A. Soha, W. J. Spalding, L. Spiegel, S. Stoynev, J. Strait, N. Strobbe, L. Taylor, S. Tkaczyk, N. V. Tran, L. Uplegger, E. W. Vaandering, C. Vernieri, R. Vidal, M. Wang, H. A. Weber, D. Acosta, P. Avery, D. Bourilkov, A. Brinkerhoff, L. Cadamuro, A. Carnes, V. Cherepanov, F. Errico, R. D. Field, S. V. Gleyzer, B. M. Joshi, M. Kim, J. Konigsberg, A. Korytov, K. H. Lo, P. Ma, K. Matchev, N. Menendez, G. Mitselmakher, D. Rosenzweig, K. Shi, J. Wang, S. Wang, X. Zuo, Y. R. Joshi, T. Adams, A. Askew, S. Hagopian, V. Hagopian, K. F. Johnson, R. Khurana, T. Kolberg, G. Martinez, T. Perry, H. Prosper, C. Schiber, R. Yohay, J. Zhang, M. M. Baarmand, M. Hohlmann, D. Noonan, M. Rahmani, M. Saunders, F. Yumiceva, M. R. Adams, L. Apanasevich, R. R. Betts, R. Cavanaugh, X. Chen, S. Dittmer, O. Evdokimov, C. E. Gerber, D. A. Hangal, D. J. Hofman, K. Jung, C. Mills, T. Roy, M. B. Tonjes, N. Varelas, J. Viinikainen, H. Wang, X. Wang, Z. Wu, M. Alhusseini, B. Bilki, W. Clarida, K. Dilsiz, S. Durgut, R. P. Gandrajula, M. Haytmyradov, V. Khristenko, O. K. Köseyan, J.-P. Merlo, A. Mestvirishvili, A. Moeller, J. Nachtman, H. Ogul, Y. Onel, F. Ozok, A. Penzo, C. Snyder, E. Tiras, J. Wetzel, B. Blumenfeld, A. Cocoros, N. Eminizer, A. V. Gritsan, W. T. Hung, S. Kyriacou, P. Maksimovic, J. Roskes, M. Swartz, C. Baldenegro Barrera, P. Baringer, A. Bean, S. Boren, J. Bowen, A. Bylinkin, T. Isidori, S. Khalil, J. King, G. Krintiras, A. Kropivnitskaya, C. Lindsey, D. Majumder, W. Mcbrayer, N. Minafra, M. Murray, C. Rogan, C. Royon, S. Sanders, E. Schmitz, J. D. Tapia Takaki, Q. Wang, J. Williams, G. Wilson, S. Duric, A. Ivanov, K. Kaadze, D. Kim, Y. Maravin, D. R. Mendis, T. Mitchell, A. Modak, A. Mohammadi, F. Rebassoo, D. Wright, A. Baden, O. Baron, A. Belloni, S. C. Eno, Y. Feng, N. J. Hadley, S. Jabeen, G. Y. Jeng, R. G. Kellogg, J. Kunkle, A. C. Mignerey, S. Nabili, F. Ricci-Tam, M. Seidel, Y. H. Shin, A. Skuja, S. C. Tonwar, K. Wong, D. Abercrombie, B. Allen, A. Baty, R. Bi, S. Brandt, W. Busza, I. A. Cali, M. D’Alfonso, G. Gomez Ceballos, M. Goncharov, P. Harris, D. Hsu, M. Hu, M. Klute, D. Kovalskyi, Y.-J. Lee, P. D. Luckey, B. Maier, A. C. Marini, C. Mcginn, C. Mironov, S. Narayanan, X. Niu, C. Paus, D. Rankin, C. Roland, G. Roland, Z. Shi, G. S. F. Stephans, K. Sumorok, K. Tatar, D. Velicanu, J. Wang, T. W. Wang, B. Wyslouch, R. M. Chatterjee, A. Evans, S. Guts, P. Hansen, J. Hiltbrand, Y. Kubota, Z. Lesko, J. Mans, R. Rusack, M. A. Wadud, J. G. Acosta, S. Oliveros, K. Bloom, S. Chauhan, D. R. Claes, C. Fangmeier, L. Finco, F. Golf, R. Kamalieddin, I. Kravchenko, J. E. Siado, G. R. Snow, B. Stieger, W. Tabb, G. Agarwal, C. Harrington, I. Iashvili, A. Kharchilava, C. McLean, D. Nguyen, A. Parker, J. Pekkanen, S. Rappoccio, B. Roozbahani, G. Alverson, E. Barberis, C. Freer, Y. Haddad, A. Hortiangtham, G. Madigan, B. Marzocchi, D. M. Morse, T. Orimoto, L. Skinnari, A. Tishelman-Charny, T. Wamorkar, B. Wang, A. Wisecarver, D. Wood, S. Bhattacharya, J. Bueghly, T. Gunter, K. A. Hahn, N. Odell, M. H. Schmitt, K. Sung, M. Trovato, M. Velasco, R. Bucci, N. Dev, R. Goldouzian, M. Hildreth, K. Hurtado Anampa, C. Jessop, D. J. Karmgard, K. Lannon, W. Li, N. Loukas, N. Marinelli, I. Mcalister, F. Meng, C. Mueller, Y. Musienko, M. Planer, R. Ruchti, P. Siddireddy, G. Smith, S. Taroni, M. Wayne, A. Wightman, M. Wolf, A. Woodard, J. Alimena, B. Bylsma, L. S. Durkin, B. Francis, C. Hill, W. Ji, A. Lefeld, T. Y. Ling, B. L. Winer, G. Dezoort, P. Elmer, J. Hardenbrook, N. Haubrich, S. Higginbotham, A. Kalogeropoulos, S. Kwan, D. Lange, M. T. Lucchini, J. Luo, D. Marlow, K. Mei, I. Ojalvo, J. Olsen, C. Palmer, P. Piroué, J. Salfeld-Nebgen, D. Stickland, C. Tully, Z. Wang, S. Malik, S. Norberg, A. Barker, V. E. Barnes, S. Das, L. Gutay, M. Jones, A. W. Jung, A. Khatiwada, B. Mahakud, D. H. Miller, G. Negro, N. Neumeister, C. C. Peng, S. Piperov, H. Qiu, J. F. Schulte, N. Trevisani, F. Wang, R. Xiao, W. Xie, T. Cheng, J. Dolen, N. Parashar, U. Behrens, K. M. Ecklund, S. Freed, F. J. M. Geurts, M. Kilpatrick, Arun Kumar, W. Li, B. P. Padley, R. Redjimi, J. Roberts, J. Rorie, W. Shi, A. G. Stahl Leiton, Z. Tu, A. Zhang, A. Bodek, P. de Barbaro, R. Demina, J. L. Dulemba, C. Fallon, T. Ferbel, M. Galanti, A. Garcia-Bellido, O. Hindrichs, A. Khukhunaishvili, E. Ranken, R. Taus, B. Chiarito, J. P. Chou, A. Gandrakota, Y. Gershtein, E. Halkiadakis, A. Hart, M. Heindl, E. Hughes, S. Kaplan, I. Laflotte, A. Lath, R. Montalvo, K. Nash, M. Osherson, H. Saka, S. Salur, S. Schnetzer, S. Somalwar, R. Stone, S. Thomas, H. Acharya, A. G. Delannoy, S. Spanier, O. Bouhali, A. Celik, M. Dalchenko, M. De Mattia, A. Delgado, S. Dildick, R. Eusebi, J. Gilmore, T. Huang, T. Kamon, S. Luo, S. Malhotra, D. Marley, R. Mueller, D. Overton, L. Perniè, D. Rathjens, A. Safonov, N. Akchurin, J. Damgov, F. De Guio, S. Kunori, K. Lamichhane, S. W. Lee, T. Mengke, S. Muthumuni, T. Peltola, S. Undleeb, I. Volobouev, Z. Wang, A. Whitbeck, S. Greene, A. Gurrola, R. Janjam, W. Johns, C. Maguire, A. Melo, H. Ni, K. Padeken, F. Romeo, P. Sheldon, S. Tuo, J. Velkovska, M. Verweij, M. W. Arenton, P. Barria, B. Cox, G. Cummings, J. Hakala, R. Hirosky, M. Joyce, A. Ledovskoy, C. Neu, B. Tannenwald, Y. Wang, E. Wolfe, F. Xia, R. Harr, P. E. Karchin, N. Poudyal, J. Sturdy, P. Thapa, T. Bose, J. Buchanan, C. Caillol, D. Carlsmith, S. Dasu, I. De Bruyn, L. Dodd, F. Fiori, C. Galloni, B. Gomber, H. He, M. Grothe, M. Herndon, A. Hervé, U. Hussain, P. Klabbers, A. Lanaro, A. Loeliger, K. Long, R. Loveless, J. Madhusudanan Sreekala, D. Pinna, T. Ruggles, A. Savin, V. Sharma, W. H. Smith, D. Teague, S. Trembath-reichert, N. Woods, CMS Collaboration

**Affiliations:** 1 Yerevan Physics Institute, Yerevan, Armenia; 2 Institut für Hochenergiephysik, Wien, Austria; 3 Institute for Nuclear Problems, Minsk, Belarus; 4 Universiteit Antwerpen, Antwerpen, Belgium; 5 Vrije Universiteit Brussel, Brussel, Belgium; 6 Université Libre de Bruxelles, Bruxelles, Belgium; 7 Ghent University, Ghent, Belgium; 8 Université Catholique de Louvain, Louvain-la-Neuve, Belgium; 9 Centro Brasileiro de Pesquisas Fisicas, Rio de Janeiro, Brazil; 10 Universidade do Estado do Rio de Janeiro, Rio de Janeiro, Brazil; 11 Universidade Estadual Paulista, Universidade Federal do ABC, São Paulo, Brazil; 12 Institute for Nuclear Research and Nuclear Energy, Bulgarian Academy of Sciences, Sofia, Bulgaria; 13 University of Sofia, Sofia, Bulgaria; 14 Beihang University, Beijing, China; 15 Institute of High Energy Physics, Beijing, China; 16 State Key Laboratory of Nuclear Physics and Technology, Peking University, Beijing, China; 17 Tsinghua University, Beijing, China; 18 Zhejiang University, Hangzhou, China; 19 Universidad de Los Andes, Bogota, Colombia; 20 Universidad de Antioquia, Medellin, Colombia; 21 University of Split, Faculty of Electrical Engineering, Mechanical Engineering and Naval Architecture, Split, Croatia; 22 University of Split, Faculty of Science, Split, Croatia; 23 Institute Rudjer Boskovic, Zagreb, Croatia; 24 University of Cyprus, Nicosia, Cyprus; 25 Charles University, Prague, Czech Republic; 26 Escuela Politecnica Nacional, Quito, Ecuador; 27 Universidad San Francisco de Quito, Quito, Ecuador; 28 Academy of Scientific Research and Technology of the Arab Republic of Egypt, Egyptian Network of High Energy Physics, Cairo, Egypt; 29 National Institute of Chemical Physics and Biophysics, Tallinn, Estonia; 30 Department of Physics, University of Helsinki, Helsinki, Finland; 31 Helsinki Institute of Physics, Helsinki, Finland; 32 Lappeenranta University of Technology, Lappeenranta, Finland; 33 IRFU, CEA, Université Paris-Saclay, Gif-sur-Yvette, France; 34 Laboratoire Leprince-Ringuet, Ecole polytechnique, CNRS/IN2P3, Université Paris-Saclay, Palaiseau, France; 35 Université de Strasbourg, CNRS, IPHC UMR 7178, Strasbourg, France; 36 Centre de Calcul de l’Institut National de Physique Nucleaire et de Physique des Particules, CNRS/IN2P3, Villeurbanne, France; 37 Université de Lyon, Université Claude Bernard Lyon 1, CNRS-IN2P3, Institut de Physique Nucléaire de Lyon, Villeurbanne, France; 38 Georgian Technical University, Tbilisi, Georgia; 39 Tbilisi State University, Tbilisi, Georgia; 40 RWTH Aachen University, I. Physikalisches Institut, Aachen, Germany; 41 RWTH Aachen University, III. Physikalisches Institut A, Aachen, Germany; 42 RWTH Aachen University, III. Physikalisches Institut B, Aachen, Germany; 43 Deutsches Elektronen-Synchrotron, Hamburg, Germany; 44 University of Hamburg, Hamburg, Germany; 45 Karlsruher Institut fuer Technologie, Karlsruhe, Germany; 46 Institute of Nuclear and Particle Physics (INPP), NCSR Demokritos, Aghia Paraskevi, Greece; 47 National and Kapodistrian University of Athens, Athens, Greece; 48 National Technical University of Athens, Athens, Greece; 49 University of Ioánnina, Ioánnina, Greece; 50 MTA-ELTE Lendület CMS Particle and Nuclear Physics Group, Eötvös Loránd University, Budapest, Hungary; 51 Wigner Research Centre for Physics, Budapest, Hungary; 52 Institute of Nuclear Research ATOMKI, Debrecen, Hungary; 53 Institute of Physics, University of Debrecen, Debrecen, Hungary; 54 Eszterhazy Karoly University, Karoly Robert Campus, Gyongyos, Hungary; 55 Indian Institute of Science (IISc), Bangalore, India; 56 National Institute of Science Education and Research, HBNI, Bhubaneswar, India; 57 Panjab University, Chandigarh, India; 58 University of Delhi, Delhi, India; 59 Saha Institute of Nuclear Physics, HBNI, Kolkata, India; 60 Indian Institute of Technology Madras, Madras, India; 61 Bhabha Atomic Research Centre, Mumbai, India; 62 Tata Institute of Fundamental Research-A, Mumbai, India; 63 Tata Institute of Fundamental Research-B, Mumbai, India; 64 Indian Institute of Science Education and Research (IISER), Pune, India; 65 Institute for Research in Fundamental Sciences (IPM), Tehran, Iran; 66 University College Dublin, Dublin, Ireland; 67 INFN Sezione di Bari, Università di Bari, Politecnico di Bari, Bari, Italy; 68 INFN Sezione di Bologna, Università di Bologna, Bologna, Italy; 69 INFN Sezione di Catania, Università di Catania, Catania, Italy; 70 INFN Sezione di Firenze, Università di Firenze, Firenze, Italy; 71 INFN Laboratori Nazionali di Frascati, Frascati, Italy; 72 INFN Sezione di Genova, Università di Genova, Genova, Italy; 73 INFN Sezione di Milano-Bicocca, Università di Milano-Bicocca, Milano, Italy; 74 INFN Sezione di Napoli, Università di Napoli ’Federico II’ , Napoli, Italy, Università della Basilicata, Potenza, Italy, Università G. Marconi, Roma, Italy; 75 INFN Sezione di Padova, Università di Padova, Padova, Italy, Università di Trento, Trento, Italy; 76 INFN Sezione di Pavia, Università di Pavia, Pavia, Italy; 77 INFN Sezione di Perugia, Università di Perugia, Perugia, Italy; 78 INFN Sezione di Pisa, Università di Pisa, Scuola Normale Superiore di Pisa, Pisa, Italy; 79 INFN Sezione di Roma, Sapienza Università di Roma, Rome, Italy; 80 INFN Sezione di Torino, Università di Torino, Torino, Italy, Università del Piemonte Orientale, Novara, Italy; 81 INFN Sezione di Trieste, Università di Trieste, Trieste, Italy; 82 Kyungpook National University, Daegu, Korea; 83 Chonnam National University, Institute for Universe and Elementary Particles, Kwangju, Korea; 84 Hanyang University, Seoul, Korea; 85 Korea University, Seoul, Korea; 86 Department of Physics, Kyung Hee University, Seoul, South Korea; 87 Sejong University, Seoul, Korea; 88 Seoul National University, Seoul, Korea; 89 University of Seoul, Seoul, Korea; 90 Sungkyunkwan University, Suwon, Korea; 91 Riga Technical University, Riga, Latvia; 92 Vilnius University, Vilnius, Lithuania; 93 National Centre for Particle Physics, Universiti Malaya, Kuala Lumpur, Malaysia; 94 Universidad de Sonora (UNISON), Hermosillo, Mexico; 95 Centro de Investigacion y de Estudios Avanzados del IPN, Mexico City, Mexico; 96 Universidad Iberoamericana, Mexico City, Mexico; 97 Benemerita Universidad Autonoma de Puebla, Puebla, Mexico; 98 Universidad Autónoma de San Luis Potosí, San Luis Potosí, Mexico; 99 University of Montenegro, Podgorica, Montenegro; 100 University of Auckland, Auckland, New Zealand; 101 University of Canterbury, Christchurch, New Zealand; 102 National Centre for Physics, Quaid-I-Azam University, Islamabad, Pakistan; 103 AGH University of Science and Technology Faculty of Computer Science, Electronics and Telecommunications, Krakow, Poland; 104 National Centre for Nuclear Research, Swierk, Poland; 105 Institute of Experimental Physics, Faculty of Physics, University of Warsaw, Warsaw, Poland; 106 Laboratório de Instrumentação e Física Experimental de Partículas, Lisboa, Portugal; 107 Joint Institute for Nuclear Research, Dubna, Russia; 108 Petersburg Nuclear Physics Institute, Gatchina (St. Petersburg), Russia; 109 Institute for Nuclear Research, Moscow, Russia; 110 Institute for Theoretical and Experimental Physics named by A.I. Alikhanov of NRC ‘Kurchatov Institute’, Moscow, Russia; 111 Moscow Institute of Physics and Technology, Moscow, Russia; 112 National Research Nuclear University ’Moscow Engineering Physics Institute’ (MEPhI), Moscow, Russia; 113 P.N. Lebedev Physical Institute, Moscow, Russia; 114 Skobeltsyn Institute of Nuclear Physics, Lomonosov Moscow State University, Moscow, Russia; 115 Novosibirsk State University (NSU), Novosibirsk, Russia; 116 Institute for High Energy Physics of National Research Centre ‘Kurchatov Institute’, Protvino, Russia; 117 National Research Tomsk Polytechnic University, Tomsk, Russia; 118 Tomsk State University, Tomsk, Russia; 119 University of Belgrade: Faculty of Physics and VINCA Institute of Nuclear Sciences, Belgrade, Serbia; 120 Centro de Investigaciones Energéticas Medioambientales y Tecnológicas (CIEMAT), Madrid, Spain; 121 Universidad Autónoma de Madrid, Madrid, Spain; 122 Universidad de Oviedo, Instituto Universitario de Ciencias y Tecnologías Espaciales de Asturias (ICTEA), Oviedo, Spain; 123 Instituto de Física de Cantabria (IFCA), CSIC-Universidad de Cantabria, Santander, Spain; 124 University of Colombo, Colombo, Sri Lanka; 125 Department of Physics, University of Ruhuna, Matara, Sri Lanka; 126 CERN, European Organization for Nuclear Research, Geneva, Switzerland; 127 Paul Scherrer Institut, Villigen, Switzerland; 128 ETH Zurich-Institute for Particle Physics and Astrophysics (IPA), Zurich, Switzerland; 129 Universität Zürich, Zurich, Switzerland; 130 National Central University, Chung-Li, Taiwan; 131 National Taiwan University (NTU), Taipei, Taiwan; 132 Chulalongkorn University, Faculty of Science, Department of Physics, Bangkok, Thailand; 133 ukurova University, Physics Department, Science and Art Faculty, Adana, Turkey; 134 Middle East Technical University, Physics Department, Ankara, Turkey; 135 Bogazici University, Istanbul, Turkey; 136 Istanbul Technical University, Istanbul, Turkey; 137 Istanbul University, Istanbul, Turkey; 138 Institute for Scintillation Materials of National Academy of Science of Ukraine, Kharkov, Ukraine; 139 National Scientific Center, Kharkov Institute of Physics and Technology, Kharkov, Ukraine; 140 University of Bristol, Bristol, United Kingdom; 141 Rutherford Appleton Laboratory, Didcot, United Kingdom; 142 Imperial College, London, United Kingdom; 143 Brunel University, Uxbridge, United Kingdom; 144 Baylor University, Waco, USA; 145 Catholic University of America, Washington DC, USA; 146 The University of Alabama, Tuscaloosa, USA; 147 Boston University, Boston, USA; 148 Brown University, Providence, USA; 149 University of California, Davis, Davis USA; 150 University of California, Los Angeles, USA; 151 University of California, Riverside, Riverside, USA; 152 University of California, San Diego, La Jolla, USA; 153 University of California, Santa Barbara-Department of Physics, Santa Barbara, USA; 154 California Institute of Technology, Pasadena, USA; 155 Carnegie Mellon University, Pittsburgh, USA; 156 University of Colorado Boulder, Boulder, USA; 157 Cornell University, Ithaca, USA; 158 Fermi National Accelerator Laboratory, Batavia, USA; 159 University of Florida, Gainesville, USA; 160 Florida International University, Miami, USA; 161 Florida State University, Tallahassee, USA; 162 Florida Institute of Technology, Melbourne, USA; 163 University of Illinois at Chicago (UIC), Chicago, USA; 164 The University of Iowa, Iowa City, USA; 165 Johns Hopkins University, Baltimore, USA; 166 The University of Kansas, Lawrence, USA; 167 Kansas State University, Manhattan, USA; 168 Lawrence Livermore National Laboratory, Livermore, USA; 169 University of Maryland, College Park, USA; 170 Massachusetts Institute of Technology, Cambridge, USA; 171 University of Minnesota, Minneapolis, USA; 172 University of Mississippi, Oxford, USA; 173 University of Nebraska-Lincoln, Lincoln, USA; 174 State University of New York at Buffalo, Buffalo, USA; 175 Northeastern University, Boston, USA; 176 Northwestern University, Evanston, USA; 177 University of Notre Dame, Notre Dame, USA; 178 The Ohio State University, Columbus, USA; 179 Princeton University, Princeton, USA; 180 University of Puerto Rico, Mayaguez, USA; 181 Purdue University, West Lafayette, USA; 182 Purdue University Northwest, Hammond, USA; 183 Rice University, Houston, USA; 184 University of Rochester, Rochester, USA; 185 Rutgers, The State University of New Jersey, Piscataway, USA; 186 University of Tennessee, Knoxville, USA; 187 Texas A & M University, College Station, USA; 188 Texas Tech University, Lubbock, USA; 189 Vanderbilt University, Nashville, USA; 190 University of Virginia, Charlottesville, USA; 191 Wayne State University, Detroit, USA; 192 University of Wisconsin-Madison, Madison, WI USA; 193 CERN, 1211 Geneva 23, Switzerland

## Abstract

Two related searches for phenomena beyond the standard model (BSM) are performed using events with hadronic jets and significant transverse momentum imbalance. The results are based on a sample of proton–proton collisions at a center-of-mass energy of $13\,\text {Te}\text {V}$, collected by the CMS experiment at the LHC in 2016–2018 and corresponding to an integrated luminosity of 137$\,\text {fb}^{-1}$. The first search is inclusive, based on signal regions defined by the hadronic energy in the event, the jet multiplicity, the number of jets identified as originating from bottom quarks, and the value of the kinematic variable $M_{\mathrm {T2}}$ for events with at least two jets. For events with exactly one jet, the transverse momentum of the jet is used instead. The second search looks in addition for disappearing tracks produced by BSM long-lived charged particles that decay within the volume of the tracking detector. No excess event yield is observed above the predicted standard model background. This is used to constrain a range of BSM models that predict the following: the pair production of gluinos and squarks in the context of supersymmetry models conserving *R*-parity, with or without intermediate long-lived charginos produced in the decay chain; the resonant production of a colored scalar state decaying to a massive Dirac fermion and a quark; or the pair production of scalar and vector leptoquarks each decaying to a neutrino and a top, bottom, or light-flavor quark. In most of the cases, the results obtained are the most stringent constraints to date.

## Introduction

We present results of two related searches for physics beyond the standard model (BSM) in events with jets and significant transverse momentum imbalance. These are based on a data set of proton–proton (${\text {p}} {\text {p}}$) collisions at $\sqrt{s} = 13\,\text {Te}\text {V}$, collected with the CMS detector at the CERN LHC in 2016–2018, and corresponding to an integrated luminosity of 137$\,\text {fb}^{-1}$.

The first is an inclusive search that exploits the transverse momentum imbalance as inferred from the kinematic variable $M_{\mathrm {T2}}$ [1], defined in Sect. 3.1, in events with at least two hadronic jets, or the transverse momentum ($p_{\mathrm {T}}$) of the jet in events with just one jet. Similar searches were previously conducted by both the ATLAS [2–7] and CMS [8–12] Collaborations. Our analysis builds on the work presented in Refs. [9, 11], using improved methods to estimate the background from standard model (SM) processes, in particular the multijet background arising from instrumental effects. Event counts in bins of the number of jets ($N_{\mathrm {j}}$), the number of jets identified as originating from the fragmentation of a bottom quark (b-tagged jets, $N_{{\text {b}}}$), the scalar $p_{\mathrm {T}}$ sum of all selected jets ($H_{\mathrm {T}}$), and the $M_{\mathrm {T2}}$ variable or the $p_{\mathrm {T}}$ of the single jet, are compared against estimates of the background from SM processes, as derived from dedicated data control samples.

The second search aims at extending the sensitivity of the inclusive search for scenarios where the mass spectrum of potential new particles is compressed. In such scenarios, some theoretical models [13, 14] predict the existence of long-lived charged particles that can be identified as disappearing tracks, when they decay within the volume of the tracking detector and their charged decay products are below the $p_{\mathrm {T}}$ detection threshold. Such signatures are rare in the SM and are often dominated by instrumental effects. The presence of disappearing tracks is exploited in order to suppress the background from SM processes, and to enhance the sensitivity towards these scenarios. Similar analyses were previously conducted by both the ATLAS [15, 16] and CMS [17–20] Collaborations. We use events with at least two jets, and the $M_{\mathrm {T2}}$ variable to further suppress the background from SM processes. Event counts in bins of $N_{\mathrm {j}}$, $H_{\mathrm {T}}$, disappearing track length, and disappearing track $p_{\mathrm {T}}$ are compared against estimates of the background from SM processes derived from dedicated data control samples.

The results are interpreted in the context of simplified models [21–25] of *R*-parity [26] conserving supersymmetry (SUSY) [27–34] where gluinos and squarks are pair-produced and the lightest SUSY particle is a neutralino.

The results of the inclusive $M_{\mathrm {T2}}$ search are also interpreted in the context of a BSM scenario where a colored scalar state $\phi$ is resonantly produced through coupling to quarks, and decays to an invisible massive Dirac fermion $\psi$ and an SM quark. This is referred to as the mono-$\phi$ model. It has been recently proposed as an explanation of an excess in data in regions with low jet multiplicities, identified in the context of a reinterpretation [35, 36] of the results of the previous inclusive $M_{\mathrm {T2}}$ search [9] as well as of other similar searches by both the ATLAS [6, 7] and CMS [8, 37] Collaborations.

Finally, the inclusive $M_{\mathrm {T2}}$ search is interpreted using models of leptoquark (LQ) pair production, similarly to Ref. [11]. Leptoquarks are hypothetical particles with quantum numbers of both quarks and leptons [38]. The spin of an LQ state is either 0 (scalar LQ or $\mathrm {LQ_{S}}$) or 1 (vector LQ or $\mathrm {LQ_{V}}$). Leptoquarks appear in BSM theories such as grand unified theories [38–41], technicolor models [42–45], compositeness scenarios [46, 47], and *R*-parity violating SUSY [27–34, 48], and have been suggested as an explanation of the anomalies observed in flavor physics [49–55] by the BaBar [56, 57], Belle [58–62], and LHCb [63–68] Collaborations. The best fit model of Refs. [54, 55] predicts an $\mathrm {LQ_{V}}$ with a mass of ${\mathcal {O}}\left( \text {Te}\text {V} {}\right)$ decaying with 50% branching fraction to either a top quark and a neutrino (${\text {t}} {{\upnu }}$) or a bottom quark and a $\uptau$ lepton (${\text {b}} {\uptau }$), which would be expected to be visible at the LHC. The final states and kinematic variables resulting from the pair production of $\mathrm {LQ_{S}}$, each decaying to a quark and a neutrino, are the same as those considered in searches for squark pair production in *R*-parity conserving SUSY, assuming that the squark decays directly to a quark and a massless neutralino [11, 69]. The decay products of $\mathrm {LQ_{V}}$ are also found to have similar kinematic properties [11, 69]. Therefore, as the search presented in this paper is already optimized for squark pair production, it is also sensitive to LQ pair production. The LQ production with decays to a quark and a neutrino has been constrained using LHC data by both the ATLAS [70–72] and CMS [11, 73–77] Collaborations, either by reinterpreting the existing squark searches, or considering scenarios with mixed branching fractions where an LQ also decays to a quark and a charged lepton. The same signatures have been previously covered at the Fermilab Tevatron by the CDF (e.g., in Refs. [78–80]) and D0 (e.g., in Refs. [81–83]) Collaborations. Constraints have been placed by direct searches for single LQ production performed at HERA by the H1 [84] and ZEUS [85] Collaborations. Finally, searches for LQs decaying to ${\text {b}} {\uptau }$ have been performed by the ATLAS [86], CMS [87, 88], CDF [89, 90], and D0 [91] Collaborations.

After a brief description of the CMS detector in Sect. 2, the event selection and categorization as well as details of the Monte Carlo (MC) simulation are presented in Sect. 3. Section 4 describes the SM background estimation. Results and their interpretations are presented in Sects. 5 and 6, respectively. Finally, a summary is provided in Sect. 7.

## The CMS detector

The central feature of the CMS apparatus is a superconducting solenoid of 6$\,\,\text {m}$ internal diameter providing a magnetic field of 3.8$\,\,\text {T}$. Within the solenoid volume are a silicon pixel and strip tracker, a lead tungstate crystal electromagnetic calorimeter, and a brass and scintillator hadron calorimeter, each composed of a barrel and two endcap sections. Forward calorimeters extend the pseudorapidity ($\eta$) coverage provided by the barrel and endcap detectors. Muons are measured in gas-ionization detectors embedded in the steel flux-return yoke outside the solenoid. The first level of the CMS trigger system, composed of custom hardware processors, uses information from the calorimeters and muon detectors to select the most interesting events in a fixed time interval of less than 4$\,\upmu \text {s}$. The high-level trigger processor farm further decreases the event rate from around 100 $\,\,\text {kHz}$ to about 1$\,\,\text {kHz}$, before data storage. A more detailed description of the CMS detector and trigger system, together with a definition of the coordinate system used and the relevant kinematic variables, can be found in Refs. [92, 93]. The pixel tracker was upgraded before the start of the data taking period in 2017, providing one additional layer of measurements compared to the older tracker [94].

## Event selection and Monte Carlo simulation

### Event selection

Events are processed using the particle-flow (PF) algorithm [95], which aims at reconstructing and identifying each individual particle in an event, with an optimal combination of information from the elements of the CMS detector. The particles reconstructed with this algorithm are hereafter referred to as PF candidates. The physics objects and the event preselection are similar to those described in Ref. [9]; they are summarized in Table 1, and described in detail below. We select events with at least one reconstructed vertex and at least one jet, and veto events with an isolated lepton (e or $\upmu$) or an isolated charged PF candidate. The isolated charged PF candidate veto is designed to provide additional rejection against events with electrons and muons, as well as to reject hadronic $\uptau$ decays.

**Table 1:** Summary of the trigger requirements and the kinematic offline event preselection requirements on the reconstructed physics objects, for both the inclusive $M_{\mathrm {T2}}$ search and the search for disappearing tracks. Here *R* is the distance parameter of the anti-$k_{\mathrm {T}}$ algorithm. To veto leptons and tracks, the transverse mass $M_{\mathrm {T}}$ is determined using the veto object and the ${\vec p}_{\mathrm {T}}^{\text {miss}}$. The variable $p_{\mathrm {T}} ^{\text {sum}}$ is a measure of object isolation and it denotes the $p_{\mathrm {T}}$ sum of all additional PF candidates in a cone around the lepton or the track. The size of the cone is listed in the table in units of $\varDelta R \equiv \sqrt{\smash [b]{(\varDelta \phi )^2 + (\varDelta \eta )^2}}$. The lepton (track) $p_{\mathrm {T}}$ is denoted as $p_{\mathrm {T}} ^{\text {lep}}$ ($p_{\mathrm {T}} ^{\text {track}}$). Further details of the lepton selection are given in Refs. [9, 96]. The *i*th-highest $p_{\mathrm {T}}$ jet is denoted as $j_\mathrm {i}$

Trigger	2016:
	$p_{\mathrm {T}} ^\text {miss} >120\,\,\text {Ge}\text {V}$ and $H_{\mathrm {T}}^{\text {miss}} >120\,\,\text {Ge}\text {V}$, or
	$H_{\mathrm {T}} >300\,\,\text {Ge}\text {V}$ and $p_{\mathrm {T}} ^\text {miss} >110\,\,\text {Ge}\text {V}$, or
	$H_{\mathrm {T}} >900\,\,\text {Ge}\text {V}$, or jet $p_{\mathrm {T}} >450\,\,\text {Ge}\text {V}$
	2017 and 2018:
	$p_{\mathrm {T}} ^\text {miss} >120\,\,\text {Ge}\text {V}$ and $H_{\mathrm {T}}^{\text {miss}} >120\,\,\text {Ge}\text {V}$, or
	$H_{\mathrm {T}} >60\,\,\text {Ge}\text {V}$ and $p_{\mathrm {T}} ^\text {miss} >120\,\,\text {Ge}\text {V}$ and $H_{\mathrm {T}}^{\text {miss}} >120\,\,\text {Ge}\text {V}$, or
	$H_{\mathrm {T}} >500\,\,\text {Ge}\text {V}$ and $p_{\mathrm {T}} ^\text {miss} >100\,\,\text {Ge}\text {V}$ and $H_{\mathrm {T}}^{\text {miss}} >100\,\,\text {Ge}\text {V}$, or
	$H_{\mathrm {T}} >800\,\,\text {Ge}\text {V}$ and $p_{\mathrm {T}} ^\text {miss} >75\,\,\text {Ge}\text {V}$ and $H_{\mathrm {T}}^{\text {miss}} >75\,\,\text {Ge}\text {V}$, or
	$H_{\mathrm {T}} >1050\,\,\text {Ge}\text {V}$, or jet $p_{\mathrm {T}} >500\,\,\text {Ge}\text {V}$
Jet selection	$R=0.4$, $p_{\mathrm {T}} >30\,\,\text {Ge}\text {V}$, $|\eta |<2.4$
b-tagged jet selection	$p_{\mathrm {T}} >20\,\,\text {Ge}\text {V}$, $|\eta |<2.4$ and b tag
$H_{\mathrm {T}}$	$H_{\mathrm {T}} >250\,\,\text {Ge}\text {V}$
$p_{\mathrm {T}} ^\text {miss}$	$p_{\mathrm {T}} ^\text {miss} >250\,\,\text {Ge}\text {V}$ for $H_{\mathrm {T}} <1200\,\,\text {Ge}\text {V}$ or $N_{\mathrm {j}} =1$, else $p_{\mathrm {T}} ^\text {miss} >30\,\,\text {Ge}\text {V}$
	$$\varDelta \phi _{\text {min}} = \varDelta \phi \left( {\vec p}_{\mathrm {T}}^{\text {miss}}, j_{\mathrm {1,2,3,4}}\right) >0.3$$
	$$|{\vec p}_{\mathrm {T}}^{\text {miss}}-\vec {H}_{\text {T}}^{\text {miss}} |/p_{\mathrm {T}} ^\text {miss} <0.5$$
$M_{\mathrm {T2}}$ (if $N_{\mathrm {j}} \ge 2$)	Inclusive $M_{\mathrm {T2}}$ search:
	$M_{\mathrm {T2}} >200\,\,\text {Ge}\text {V}$ for $H_{\mathrm {T}} <1500\,\,\text {Ge}\text {V}$, else $M_{\mathrm {T2}} >400\,\,\text {Ge}\text {V}$
	Disappearing tracks search:
	$$M_{\mathrm {T2}} >200\,\,\text {Ge}\text {V} $$
$p_{\mathrm {T}} ^{\text {sum}}$ cone (isolation)	Veto e or $\upmu$: $\varDelta R= \min (0.2, \max (10\,\,\text {Ge}\text {V}/p_{\mathrm {T}} ^{\text {lep}},0.05))$
	Veto track: $\varDelta R=0.3$
Veto electron	$p_{\mathrm {T}} >10\,\,\text {Ge}\text {V}$, $|\eta |<2.4$, $p_{\mathrm {T}} ^{\text {sum}} < 0.1 \, p_{\mathrm {T}} ^{\text {lep}}$
Veto electron track	$p_{\mathrm {T}} >5\,\,\text {Ge}\text {V}$, $|\eta |<2.4$, $M_{\mathrm {T}} <100\,\,\text {Ge}\text {V}$, $p_{\mathrm {T}} ^{\text {sum}} < 0.2 \, p_{\mathrm {T}} ^{\text {lep}}$
Veto muon	$p_{\mathrm {T}} >10\,\,\text {Ge}\text {V}$, $|\eta |<2.4$, $p_{\mathrm {T}} ^{\text {sum}} < 0.2 \,p_{\mathrm {T}} ^{\text {lep}}$
Veto muon track	$p_{\mathrm {T}} >5\,\,\text {Ge}\text {V}$, $|\eta |<2.4$, $M_{\mathrm {T}} <100\,\,\text {Ge}\text {V}$, $p_{\mathrm {T}} ^{\text {sum}} < 0.2 \, p_{\mathrm {T}} ^{\text {lep}}$
Veto track	$p_{\mathrm {T}} >10\,\,\text {Ge}\text {V}$, $|\eta |<2.4$, $M_{\mathrm {T}} <100\,\,\text {Ge}\text {V}$, $p_{\mathrm {T}} ^{\text {sum}} < 0.1 \, p_{\mathrm {T}} ^{\text {track}}$

Jets are formed by clustering PF candidates using the anti-$k_{\mathrm {T}}$ algorithm [97, 98] and are corrected for contributions from event pileup [99] and the effects of nonuniform detector response [100, 101]. Only jets passing the selection criteria in Table 1 are used for counting and for the determination of kinematic variables. In particular, we consider jets with $p_{\mathrm {T}} >30\,\,\text {Ge}\text {V}$ and $|\eta |<2.4$, unless otherwise stated. Jets that contain the decay of a bottom-flavored hadron are identified using a deep neural network algorithm [102] with a working point chosen such that the efficiency to identify a bottom quark jet is in the range 55–70% for jet $p_{\mathrm {T}}$ between 20 and 400$\,\,\text {Ge}\text {V}$. The misidentification rate is approximately 1–2% for light-flavor or gluon jets, and 10–15% for charm jets. We count b-tagged jets with $p_{\mathrm {T}} >20\,\,\text {Ge}\text {V}$ and $|\eta |<2.4$. The minimum $p_{\mathrm {T}}$ threshold used for counting b-tagged jets is lowered to 20$\,\,\text {Ge}\text {V}$ instead of 30, as used for $N_{\mathrm {j}}$, in order to maximize the sensitivity towards BSM scenarios with bottom quarks.

The negative of the vector $p_{\mathrm {T}}$ sum of all selected jets is denoted by $\vec {H}_{\text {T}}^{\text {miss}}$, while the missing transverse momentum ${\vec p}_{\mathrm {T}}^{\text {miss}}$ is defined as the negative of the vector $p_{\mathrm {T}}$ sum of all reconstructed PF candidates. Their magnitudes are referred to as $H_{\mathrm {T}}^{\text {miss}}$ and $p_{\mathrm {T}} ^\text {miss}$, respectively. The ${\vec p}_{\mathrm {T}}^{\text {miss}}$ is further adjusted to reflect the jet energy corrections [100, 101]. Events with possible contributions from beam halo processes or anomalous noise in the calorimeter are rejected using dedicated filters [103, 104]. For events with at least two jets, we start with the pair having the largest dijet invariant mass and iteratively cluster all selected jets using an algorithm that minimizes the Lund distance measure [105, 106] until two stable pseudo-jets are obtained. The resulting pseudo-jets together with the ${\vec p}_{\mathrm {T}}^{\text {miss}}$ are used to calculate the kinematic variable $M_{\mathrm {T2}}$ [1] as:

1 $$\begin{aligned} M_{\mathrm {T2}} = \min _{{\vec p}_{\mathrm {T}}^{{\text {miss}}{ \mathrm {X}(1)}} + {\vec p}_{\mathrm {T}}^{{\text {miss}}{ \mathrm {X}(2)}} = {\vec p}_{\mathrm {T}}^{\text {miss}}} \left[ \max \left( M_{\mathrm {T}} ^{(1)} , M_{\mathrm {T}} ^{(2)} \right) \right] , \end{aligned}$$

where ${\vec p}_{\mathrm {T}}^{{\text {miss}}{ \mathrm {X}(i)}}$ ($i=1,2$) are trial vectors obtained by decomposing ${\vec p}_{\mathrm {T}}^{\text {miss}}$, and $M_{\mathrm {T}} ^{(i)}$ are the transverse masses [107] obtained by pairing either of the trial vectors with one of the two pseudo-jets. The minimization is performed over all trial momenta satisfying the ${\vec p}_{\mathrm {T}}^{\text {miss}}$ constraint. The background from multijet events (discussed in Sect. 4) is characterized by small values of $M_{\mathrm {T2}}$, while processes with significant genuine ${\vec p}_{\mathrm {T}}^{\text {miss}}$ yield larger values of $M_{\mathrm {T2}}$. More detailed discussions of the $M_{\mathrm {T2}}$ variable properties are given in Refs. [96, 108, 109].

In both the inclusive $M_{\mathrm {T2}}$ search and the search for disappearing tracks, collision events are selected using triggers with requirements on $H_{\mathrm {T}}$, $p_{\mathrm {T}} ^\text {miss}$, $H_{\mathrm {T}}^{\text {miss}}$, and jet $p_{\mathrm {T}}$. The combined trigger efficiency, as measured in an orthogonal data sample of events with an isolated electron, is found to be >97% across the full kinematic range of the search. To suppress background from multijet production, we require $M_{\mathrm {T2}} > 200\,\,\text {Ge}\text {V}$ in events with $N_{\mathrm {j}} \ge 2$. In the inclusive $M_{\mathrm {T2}}$ search, this $M_{\mathrm {T2}}$ threshold is increased to 400$\,\,\text {Ge}\text {V}$ for events with $H_{\mathrm {T}} > 1500\,\,\text {Ge}\text {V}$ to maintain multijet processes as a subdominant background in all search regions. In events with $N_{\mathrm {j}} = 1$, where $M_{\mathrm {T2}}$ is not defined, we require $p_{\mathrm {T}} ^{\text {jet}} >250\,\,\text {Ge}\text {V}$ and $p_{\mathrm {T}} ^\text {miss} >250\,\,\text {Ge}\text {V}$. As a protection against jet mismeasurement, we require the minimum difference in the azimuthal angle between the ${\vec p}_{\mathrm {T}}^{\text {miss}}$ vector and the direction of each of the four $p_{\mathrm {T}}$-leading jets, $\varDelta \phi _{\text {min}}$, to be greater than 0.3 radians, and the magnitude of the difference between ${\vec p}_{\mathrm {T}}^{\text {miss}}$ and $\vec {H}_{\text {T}}^{\text {miss}}$ to be less than half of $p_{\mathrm {T}} ^\text {miss}$. For the determination of $\varDelta \phi _{\text {min}}$, we consider jets with $|\eta |<4.7$. If fewer than four such jets are found, all are considered in the $\varDelta \phi _{\text {min}}$ calculation.

In the search for disappearing tracks, events are selected requiring in addition the presence of at least one disappearing track. These are defined as well-reconstructed isolated tracks with no measurement points in at least two of the outermost layers of the tracker and no associated energy deposits in the calorimeter. These tracks are predominantly not considered as candidates by the PF algorithm; as a result they are not included in the calculation of ${\vec p}_{\mathrm {T}}^{\text {miss}}$.

### Event categorization

#### Inclusive $M_{\mathrm {T2}}$ search

Events containing at least two jets are categorized by the values of $N_{\mathrm {j}}$, $N_{{\text {b}}}$, and $H_{\mathrm {T}}$. Each category is referred to as a topological region. Signal regions are defined by further dividing topological regions into bins of $M_{\mathrm {T2}}$. Events with only one jet are selected if the jet $p_{\mathrm {T}}$ is at least 250$\,\,\text {Ge}\text {V}$, and are classified according to the $p_{\mathrm {T}}$ of this jet and whether the event contains a b-tagged jet. The 282 search regions are summarized in Tables 12, 13, 14, 15, 16, 17, 18, 19, 20, 21, 22 and 23 in Appendix B.1. We also define *super signal regions*, covering a subset of the kinematic space of the full analysis with simpler inclusive selection criteria. The super signal regions can be used to obtain approximate interpretations of our result, as discussed in Sect. 5, where these regions are defined.

#### Search for disappearing tracks

In the following, the selected disappearing tracks are called short tracks (STs). We also define short track candidates (STCs) as disappearing tracks that are required to satisfy relaxed selection criteria on the track quality and isolation compared to an ST, but not the tight ones required for STs. Both STs and STCs are required to have no measurement points in at least two of the outermost layers of the tracker and no associated energy deposits in the calorimeter.

We select events with at least one ST and at least two jets, and we categorize them by the values of $N_{\mathrm {j}}$ and $H_{\mathrm {T}}$. Disappearing tracks are categorized according to their length and $p_{\mathrm {T}}$, in order to maximize the sensitivity to a range of lifetimes of potential BSM long-lived charged particles, and to distinguish tracks reconstructed with different precision. Two bins of $p_{\mathrm {T}}$ are defined as:

- $$15<p_{\mathrm {T}} <50\,\,\text {Ge}\text {V} $$,
- $$p_{\mathrm {T}} >50\,\,\text {Ge}\text {V} $$.

Additionally, four track length categories are defined, depending on the number of layers of the tracking detector with a measurement:

- pixel tracks (P), having at least three layers with a measurement in the pixel tracking detector, and none in the strip tracking detector,
- medium length tracks (M), having less than seven layers with a measurement, and at least one outside of the pixel tracking detector,
- long tracks (L), having at least seven layers with a measurement.

For 2017–2018 data, we further split the P tracks into two categories:

- pixel tracks having three layers with a measurement (P3),
- pixel tracks having at least four layers with a measurement (P4).

For long (L) tracks, no categorization in bins of $p_{\mathrm {T}}$ is applied.

The full track selection requirements for both STs and STCs are listed in Table 11 of Appendix A, together with the track length categories they belong to. For signal STs, the track reconstruction and selection efficiency ranges from 50 to 65%, depending on the track length and the data taking period.

The 68 search regions (28 used for the categorization of the 2016 data set, and 40 for the 2017–2018 data set) are summarized in Tables 24 and 25 in Appendix B.2.

### Monte Carlo simulation

The MC simulation is used to design the search, to help estimate SM backgrounds, and to evaluate the sensitivity to simplified models of BSM physics.

The main background samples (${\text {Z}} + \text {jets}$, ${\text {W}} + \text {jets}$, ${{\text {t}} {\bar{{{\text {t}}}}}} + \text {jets}$, and multijet), as well as BSM signal samples, are generated at leading order (LO) precision with the MadGraph 5_amc@nlo  2 (2.2.2, or 2.4.2) generator [110]. Up to four, three, or two additional partons are considered in the matrix element calculations for the generation of the ${\text {V}} + \text {jets}$  $({\text {V}} ={\text {W}},{\text {Z}})$, ${{\text {t}} {\bar{{{\text {t}}}}}} + \text {jets}$, and signal samples, respectively. Other background processes are also considered: ${{\text {t}} {\bar{{{\text {t}}}}}} {\text {V}}$ samples with up to two additional partons in the matrix element calculations are generated at LO precision with the MadGraph 5_amc@nlo  2 generator, while single top quark samples are generated at next-to-leading order (NLO) precision with the MadGraph 5_amc@nlo  2 or powheg  ($\mathrm {v}1.0$, or $\mathrm {v}2.0$) [111–115] generators. Finally, contributions from rarer processes such as diboson, triboson, and four top quark production, are also considered and found to be negligible. The expected yields of all samples are normalized using the most precise available cross section calculations, typically corresponding to NLO or next-to-NLO (NNLO) accuracy [110, 113, 115–119].

The detector response of SM samples and 2016 signal samples containing long-lived objects is modeled with the Geant4 [120] program, while the CMS fast simulation framework [121, 122] is used for other signal samples, and uncertainties are derived to account for the potential mismodeling of the event kinematics.

For all simulated samples, generators are interfaced with pythia  8.2 (8.205, 8.212, 8.226, or 8.230) [123] for fragmentation and parton showering. For samples simulated at LO (NLO) precision, the MLM [124] (FxFx [125]) prescription is used to match partons from the matrix element calculation to those from the parton showers. The CUETP8M1 [126] pythia  8.2 tune is used for the 2016 SM background and signal samples. For 2017 and 2018, the CP5 and CP2 tunes [127] are used for the SM background and signal samples, respectively. The NNPDF2.3LO (NNPDF2.3NLO) [128] parton distribution functions (PDFs) are used to generate the 2016 LO (NLO) samples, while the NNPDF3.1LO (NNPDF3.1NNLO) [129] PDFs are used for the 2017 and 2018 samples.

The output of the detector simulation is processed using the same chain of reconstruction algorithms as for collision data.

To improve on the MadGraph 5_amc@nlo modeling of the multiplicity of additional jets from initial-state radiation (ISR) in the 2016 sample, MadGraph 5_amc@nlo  t $\bar{{{\text {t}}}}$ MC events are weighted based on the number of ISR jets ($N_\mathrm {j}^\mathrm {ISR}$) so as to make the jet multiplicity agree with data. The same reweighting procedure is applied to BSM MC events. The weighting factors are obtained from a control region enriched in t $\bar{{{\text {t}}}}$, defined as events with two leptons and exactly two b-tagged jets, and vary between 0.92 for $N_\mathrm {j}^\mathrm {ISR}=1$ and 0.51 for $N_\mathrm {j}^\mathrm {ISR}\ge 6$. We take one half of the deviation from unity as the systematic uncertainty in these reweighting factors, to cover for the experimental uncertainties in their derivation and for differences between t $\bar{{{\text {t}}}}$ and BSM production. Owing to a better tuning of the MC generators, this reweighting procedure is not necessary for 2017 and 2018 MadGraph 5_amc@nlo  t $\bar{{{\text {t}}}}$ MC samples, while it is still applied to BSM MC events.

To improve the modeling of the flavor of additional jets, the simulation of t $\bar{{{\text {t}}}}$ and ${{\text {t}} {\bar{{{\text {t}}}}}} {\text {V}}$ events is corrected to account for the measured ratio of ${{\text {t}} {\bar{{{\text {t}}}}}} {\text {b}} {\bar{{{\text {b}}}}}$/${{\text {t}} {\bar{{{\text {t}}}}}} \text {jj}$ cross sections reported in Ref. [130]. Specifically, simulated t $\bar{{{\text {t}}}}$ and ${{\text {t}} {\bar{{{\text {t}}}}}} {\text {V}}$ events with two b quarks not originating from top quark decay are weighted to account for the CMS measurement of the ratio of cross sections $\sigma ({{\text {t}} {\bar{{{\text {t}}}}}} {\text {b}} {\bar{{{\text {b}}}}} )/\sigma ({{\text {t}} {\bar{{{\text {t}}}}}} \text {jj})$, which was found to be a factor of $1.7 \pm 0.5$ larger than the MC prediction [130].

## Background estimation

### Inclusive $M_{\mathrm {T2}}$ search

The backgrounds in jets-plus-$p_{\mathrm {T}} ^\text {miss}$ final states arise from three categories of SM processes.

- The lost-lepton (LL) background: events with a lepton from a W boson decay where the lepton is either out of acceptance, not reconstructed, not identified, or not isolated. This background originates mostly from ${\text {W}} + \text {jets}$ and ${{\text {t}} {\bar{{{\text {t}}}}}} + \text {jets}$ events, with smaller contributions from more rare processes, such as diboson or ${{\text {t}} {\bar{{{\text {t}}}}}} {\text {V}}$ production.
- The irreducible background: ${\text {Z}} + \text {jets}$ events, where the Z boson decays to neutrinos. This background is the most difficult to distinguish from the final states arising from potential signals. It is a major background in nearly all search regions, its importance decreasing with increasing $N_{{\text {b}}}$.
- The instrumental background: mostly multijet events with no genuine $p_{\mathrm {T}} ^\text {miss}$. These events enter a search region due to either significant jet momentum mismeasurements or sources of anomalous noise. This is a subdominant background compared to others, after events are selected, as described in Sect. 3.1.

The backgrounds are estimated from data control regions. In the presence of BSM physics, these control regions could be affected by signal contamination. Although the expected signal contamination is typically negligible, its potential impact is accounted for in the interpretation of the results, as further described in Sect. 6.

#### Estimation of the background from events with leptonic W boson decays

The LL background is estimated from control regions with exactly one lepton candidate (e or $\upmu$) selected using the same triggers and preselection criteria used for the signal regions, with the exception of the lepton veto, which is inverted. The transverse mass $M_{\mathrm {T}}$ determined using the lepton candidate and the ${\vec p}_{\mathrm {T}}^{\text {miss}}$ is required to satisfy $M_{\mathrm {T}} <100\,\,\text {Ge}\text {V}$, in order to suppress the potential signal contamination of the control regions. Selected events are binned according to the same criteria as the search regions. The background in each signal bin, $N^{\mathrm {SR}}_{\mathrm {LL}}$, is obtained by scaling the number of events in the control region, $N^{\mathrm {CR}}_{1\ell }$, using transfer factors $R^{0\ell /1\ell }_{\mathrm {MC}}$, as detailed below:

- For events with $N_{\mathrm {j}} =1$:
  2 $$\begin{aligned}&N^{\mathrm {SR}}_{\mathrm {LL}} \left( p_{\mathrm {T}} ^{\text {jet}},N_{{\text {b}}} \right) \nonumber \\&\quad = N^{\mathrm {CR}}_{1\ell } \left( p_{\mathrm {T}} ^{\text {jet}},N_{{\text {b}}} \right) \, R^{0\ell /1\ell }_{\mathrm {MC}} \left( p_{\mathrm {T}} ^{\text {jet}},N_{{\text {b}}} \right) . \end{aligned}$$
- For events with $N_{\mathrm {j}} \ge 2$:
  3 $$\begin{aligned}&N^{\mathrm {SR}}_{\mathrm {LL}} \left( \varOmega ,M_{\mathrm {T2}} \right) = N^{\mathrm {CR}}_{1\ell } \left( \varOmega ,M_{\mathrm {T2}} \right) \, \nonumber \\&\quad \times R^{0\ell /1\ell }_{\mathrm {MC}} \left( \varOmega ,M_{\mathrm {T2}} \right) \, k_{\mathrm {LL}} \left( M_{\mathrm {T2}} |\varOmega \right) , \end{aligned}$$
  where:
  4 $$\begin{aligned} \varOmega \equiv \left( H_{\mathrm {T}},N_{\mathrm {j}},N_{{\text {b}}} \right) . \end{aligned}$$

The single-lepton control regions have 1–2 times as many events as the corresponding signal regions. The factor $R^{0\ell /1\ell }_{\mathrm {MC}}$ accounts for lepton acceptance and efficiency, as well as the expected contribution from the decay of W bosons to hadrons through an intermediate $\uptau$ lepton. It is obtained from MC simulation, and corrected for the measured differences in the lepton efficiencies between data and simulation.

For events with $N_{\mathrm {j}} \ge 2$, the factor $k_{\mathrm {LL}}$ is one, except at high $M_{\mathrm {T2}}$ values, where the single-lepton control sample has insufficient data to allow $N^{\mathrm {CR}}_{1\ell }$ to be measured in each ($H_{\mathrm {T}}$, $N_{\mathrm {j}}$, $N_{{\text {b}}}$, $M_{\mathrm {T2}}$) bin. In such cases, $N^{\mathrm {CR}}_{1\ell }$ is integrated over the remaining $M_{\mathrm {T2}}$ bins of the same ($H_{\mathrm {T}}$, $N_{\mathrm {j}}$, $N_{{\text {b}}}$) region, and the distribution in $M_{\mathrm {T2}}$ across these bins is taken from simulation and applied through the factor $k_{\mathrm {LL}}$.

**Fig. 1:**
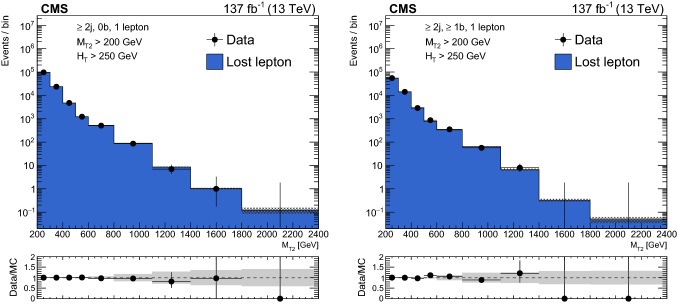
Distributions of the $M_{\mathrm {T2}}$ variable in data and simulation for the single-lepton control region, after normalizing the simulation to data in bins of $H_{\mathrm {T}}$, $N_{\mathrm {j}}$, and $N_{{\text {b}}}$, for events with no b-tagged jets (left), and events with at least one b-tagged jet (right). The hatched bands on the top panels show the MC statistical uncertainty, while the solid gray bands in the ratio plots show the systematic uncertainty in the $M_{\mathrm {T2}}$ shape. The bins have different widths, denoted by the horizontal bars

The MC modeling of $M_{\mathrm {T2}}$ is checked in data, in single-lepton events with either $N_{{\text {b}}} =0$ or $N_{{\text {b}}} \ge 1$, as shown in the left and right panels of Fig. 1, respectively. The predicted distributions in the comparison are obtained by summing all the relevant regions, after normalizing MC event yields to data and distributing events among the $M_{\mathrm {T2}}$ bins according to the expectation from simulation.

Uncertainties arising from the limited size of the control samples and from theoretical and experimental considerations are evaluated and propagated to the final estimate. The dominant uncertainty in $R^{0\ell /1\ell }_{\mathrm {MC}}$ is due to the modeling of the lepton efficiency (for electrons, muons, and hadronically decaying $\uptau$ leptons) and jet energy scale (JES), and is of order 15–20%. The uncertainty in the $M_{\mathrm {T2}}$ extrapolation via $k_{\mathrm {LL}}$, which is as large as 40%, arises primarily from the JES, the relative fractions of ${\text {W}} + \text {jets}$ and ${{\text {t}} {\bar{{{\text {t}}}}}} + \text {jets}$ events, and the choice of the renormalization ($\mu _{\mathrm {R}}$) and factorization ($\mu _{\mathrm {F}}$) scales used in the event generation.

The uncertainties in the LL background prediction are summarized in Table 2 together with their typical size ranges across the search bins.

**Table 2:** Summary of systematic uncertainties in the lost-lepton background prediction, together with their typical size ranges across the search bins

Source	Range (%)
Limited size of data control samples	5–100
Limited size of MC samples	0–50
${\text {e}}/{\upmu }$ efficiency	0–10
$\uptau$ efficiency	0–3
b tagging efficiency	0–3
Jet energy scale	0–5
$M_{\mathrm {T}} \left( \text {lepton},~{\vec p}_{\mathrm {T}}^{\text {miss}} \right)$ selection efficiency	0–3
$M_{\mathrm {T2}}$ shape uncertainty (if $k_{\mathrm {LL}}\ne 1$)	0–40
$\mu _{\mathrm {R}}$ and $\mu _{\mathrm {F}}$ variation	0–5
${{\text {t}} {\bar{{{\text {t}}}}}} {\text {b}} {\bar{{{\text {b}}}}} /{{\text {t}} {\bar{{{\text {t}}}}}} \text {jj}$ weight	0–25

#### Estimation of the background from ${\text {Z}} ({{\upnu }} \bar{{{\upnu }}})+\text {jets}$

The ${\text {Z}} \rightarrow {{\upnu }} \bar{{{\upnu }}}$ background is estimated from a ${\text {Z}} \rightarrow \ell ^{+}\ell ^{-}$ ($\ell = {\text {e}},{\upmu }$) control sample selected using dilepton triggers. The trigger efficiency, measured from a sample of events in data with large $H_{\mathrm {T}}$, is found to be greater than 97% in the selected kinematic range.

The leptons in the control sample are required to be of the same flavor and have opposite charge. The $p_{\mathrm {T}}$ of the leading and trailing leptons must be at least 100 and 30$\,\,\text {Ge}\text {V}$, respectively. Finally, the invariant mass of the lepton pair must be within 20$\,\,\text {Ge}\text {V}$ of the Z boson mass.

After requiring that the $p_{\mathrm {T}}$ of the dilepton system is at least 200$\,\,\text {Ge}\text {V}$ (corresponding to the $M_{\mathrm {T2}} >200\,\,\text {Ge}\text {V}$ requirement), the preselection requirements are applied based on kinematic variables recalculated after removing the dilepton system from the event to replicate the ${\text {Z}} \rightarrow {{\upnu }} \bar{{{\upnu }}}$ kinematic properties. For events with $N_{\mathrm {j}} = 1$, one control region is defined for each bin of jet $p_{\mathrm {T}}$. For events with at least two jets, the selected events are binned in $H_{\mathrm {T}}$, $N_{\mathrm {j}}$, and $N_{{\text {b}}}$, but not in $M_{\mathrm {T2}}$, to increase the dilepton event yield in each control region.

The contribution to each control region from flavor-symmetric processes, most importantly t $\bar{{{\text {t}}}}$ production, is estimated using different-flavor (DF) ${\text {e}} {\upmu }$ events obtained with the same selection criteria as same-flavor (SF) ${\text {e}} {\text {e}}$ and ${\upmu } {\upmu }$ events. The background in each signal bin is then obtained using transfer factors.

- For events with $N_{\mathrm {j}} =1$, according to:
  5 $$\begin{aligned}&N^{\mathrm {SR}}_{{\text {Z}} \rightarrow {{\upnu }} \bar{{{\upnu }}}} \left( p_{\mathrm {T}} ^{\text {jet}},N_{{\text {b}}} \right) = \Bigl [N^{\mathrm {CRSF}}_{\ell \ell } \left( p_{\mathrm {T}} ^{\text {jet}},N_{{\text {b}}} \right) \nonumber \\&\quad - N^{\mathrm {CRDF}}_{\ell \ell } \left( p_{\mathrm {T}} ^{\text {jet}},N_{{\text {b}}} \right) \, R^{\mathrm {SF}/\mathrm {DF}} \Bigr ] \nonumber \\&\quad \times R^{{\text {Z}} \rightarrow {{\upnu }} \bar{{{\upnu }}}/Z\rightarrow \ell ^{+}\ell ^{-}}_{\mathrm {MC}} \left( p_{\mathrm {T}} ^{\text {jet}},N_{{\text {b}}} \right) . \end{aligned}$$
- For events with $N_{\mathrm {j}} \ge 2$, according to:
  6 $$\begin{aligned}&N^{\mathrm {SR}}_{{\text {Z}} \rightarrow {{\upnu }} \bar{{{\upnu }}}} \left( \varOmega ,M_{\mathrm {T2}} \right) = \Bigl [N^{\mathrm {CRSF}}_{\ell \ell } \left( \varOmega \right) \nonumber \\&\quad - N^{\mathrm {CRDF}}_{\ell \ell } \left( \varOmega \right) \, R^{\mathrm {SF}/\mathrm {DF}} \Bigr ] \nonumber \\&\quad \times R^{{\text {Z}} \rightarrow {{\upnu }} \bar{{{\upnu }}}/Z\rightarrow \ell ^{+}\ell ^{-}}_{\mathrm {MC}} \left( \varOmega \right) \, k_{{\text {Z}} \rightarrow {{\upnu }} \bar{{{\upnu }}}}\left( M_{\mathrm {T2}} ~|~\varOmega \right) , \end{aligned}$$
  where $\varOmega$ is defined in Eq. (4).

**Fig. 2:**
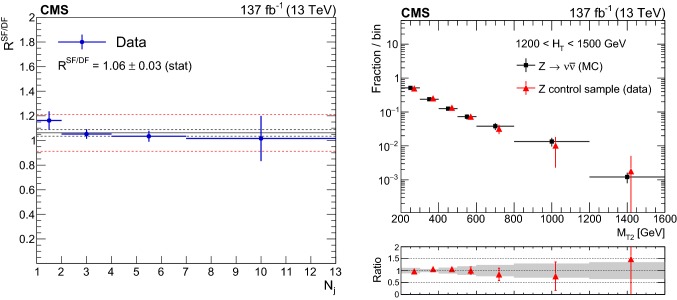
(Left) Ratio $R^{\mathrm {SF}/\mathrm {DF}}$ in data as a function of $N_{\mathrm {j}}$. The solid black line enclosed by the red dashed lines corresponds to a value of $1.06\pm 0.15$ that is observed to be stable with respect to event kinematic variables, while the two dashed black lines denote the statistical uncertainty in the $R^{\mathrm {SF}/\mathrm {DF}}$ value. (Right) The shape of the $M_{\mathrm {T2}}$ distribution in ${\text {Z}} \rightarrow {{\upnu }} \bar{{{\upnu }}}$ simulation compared to the one obtained from the ${\text {Z}} \rightarrow \ell ^{+}\ell ^{-}$ data control sample, in a region with $1200<H_{\mathrm {T}} <1500$ $\,\,\text {Ge}\text {V}$ and $N_{\mathrm {j}} \ge 2$, inclusive in $N_{{\text {b}}}$. The solid gray band on the ratio plot shows the systematic uncertainty in the $M_{\mathrm {T2}}$ shape. The bins have different widths, denoted by the horizontal bars

Here $N^{\mathrm {CRSF}}_{\ell \ell }$ and $N^{\mathrm {CRDF}}_{\ell \ell }$ are the number of SF and DF events in the control region, while $R^{{\text {Z}} \rightarrow {{\upnu }} \bar{{{\upnu }}}/{\text {Z}} \rightarrow \ell ^{+}\ell ^{-}}_{\mathrm {MC}}$ and $k_{{\text {Z}} \rightarrow {{\upnu }} \bar{{{\upnu }}}}$ are defined below. The factor $R^{\mathrm {SF}/\mathrm {DF}}$ accounts for the difference in acceptance and efficiency between SF and DF events. It is determined as the ratio of the number of SF to DF events in a t $\bar{{{\text {t}}}}$ enriched control sample, obtained with the same selection criteria as the ${\text {Z}} \rightarrow \ell ^{+}\ell ^{-}$ sample, but inverting the requirements on the $p_{\mathrm {T}}$ and the invariant mass of the lepton pair. A measured value of $R^{\mathrm {SF}/\mathrm {DF}}=1.06\pm 0.15$ is observed to be stable with respect to event kinematic variables, and is applied in all regions. Figure 2 (left) shows $R^{\mathrm {SF}/\mathrm {DF}}$ measured as a function of the number of jets.

For events with $N_{\mathrm {j}} =1$, an estimate of the ${\text {Z}} \rightarrow {{\upnu }} \bar{{{\upnu }}}$ background in each search bin is obtained from the corresponding dilepton control region via the factor $R^{{\text {Z}} \rightarrow {{\upnu }} \bar{{{\upnu }}}/{\text {Z}} \rightarrow \ell ^{+}\ell ^{-}}_{\mathrm {MC}}$, which accounts for the acceptance and efficiency to select the dilepton pair and the ratio of branching fractions for the ${\text {Z}} \rightarrow \ell ^{+}\ell ^{-}$ and ${\text {Z}} \rightarrow {{\upnu }} \bar{{{\upnu }}}$ decays. For events with at least two jets, an estimate of the ${\text {Z}} \rightarrow {{\upnu }} \bar{{{\upnu }}}$ background is obtained analogously in each ($H_{\mathrm {T}}$, $N_{\mathrm {j}}$, $N_{{\text {b}}}$) region, integrated over $M_{\mathrm {T2}}$. The factor $R^{{\text {Z}} \rightarrow {{\upnu }} \bar{{{\upnu }}}/{\text {Z}} \rightarrow \ell ^{+}\ell ^{-}}_{\mathrm {MC}}$ is obtained from simulation, including corrections for the differences in the lepton efficiencies between data and simulation.

For events with $N_{\mathrm {j}} \ge 2$, the factor $k_{{\text {Z}} \rightarrow {{\upnu }} \bar{{{\upnu }}}}$ accounts for the distribution in bins of $M_{\mathrm {T2}}$ of the estimated background in each ($H_{\mathrm {T}}$, $N_{\mathrm {j}}$, $N_{{\text {b}}}$) region. This distribution is constructed using $M_{\mathrm {T2}}$ shape templates from dilepton data and ${\text {Z}} \rightarrow {{\upnu }} \bar{{{\upnu }}}$ simulation in each ($H_{\mathrm {T}}$, $N_{\mathrm {j}}$, $N_{{\text {b}}}$) region. The templates obtained from data are used at low values of $M_{\mathrm {T2}}$, where the amount of data is sufficient. On the other hand, at high values of $M_{\mathrm {T2}}$ we use the templates from simulation.

Studies with simulated samples have demonstrated that the shape of the $M_{\mathrm {T2}}$ distribution of the function $k_{{\text {Z}} \rightarrow {{\upnu }} \bar{{{\upnu }}}}$ is independent of $N_{{\text {b}}}$ for a given $H_{\mathrm {T}}$ and $N_{\mathrm {j}}$ selection, and that the shape is also independent of $N_{\mathrm {j}}$ for $H_{\mathrm {T}} >1500\,\,\text {Ge}\text {V}$. The dilepton control sample supports this observation. Therefore, functions $k_{{\text {Z}} \rightarrow {{\upnu }} \bar{{{\upnu }}}}$ are obtained for each ($H_{\mathrm {T}}$, $N_{\mathrm {j}}$) region, integrated over $N_{{\text {b}}}$. For $H_{\mathrm {T}} >1500\,\,\text {Ge}\text {V}$, only one function $k_{{\text {Z}} \rightarrow {{\upnu }} \bar{{{\upnu }}}}$ is constructed, integrating also over $N_{\mathrm {j}}$.

The MC modeling of the $M_{\mathrm {T2}}$ variable is validated in data using control samples enriched in ${\text {Z}} \rightarrow \ell ^{+}\ell ^{-}$ events, in each bin of $H_{\mathrm {T}}$, as shown in the right panel of Fig. 2 for events with $1200<H_{\mathrm {T}} <1500\,\,\text {Ge}\text {V}$.

The largest uncertainty in the estimate of the invisible Z background in most regions results from the limited size of the dilepton control sample. The dominant uncertainty of about 5% in the ratio $R^{Z\rightarrow {{\upnu }} \bar{{{\upnu }}}/Z\rightarrow \ell ^{+}\ell ^{-}}_{\mathrm {MC}}$ reflects the uncertainty in the differences between the lepton efficiencies in data and simulation. The uncertainty in the $k_{{\text {Z}} \rightarrow {{\upnu }} \bar{{{\upnu }}}}$ factor arises from data statistical uncertainty for bins at low values of $M_{\mathrm {T2}}$, where the function $k_{{\text {Z}} \rightarrow {{\upnu }} \bar{{{\upnu }}}}$ is obtained from data, while for bins at high values of $M_{\mathrm {T2}}$, where the function $k_{{\text {Z}} \rightarrow {{\upnu }} \bar{{{\upnu }}}}$ is obtained from simulation, it is due to the uncertainties in the JES and the choice of the $\mu _{\mathrm {R}}$ and $\mu _{\mathrm {F}}$. These can result in effects as large as 40%.

The uncertainties in the ${\text {Z}} \rightarrow {{\upnu }} \bar{{{\upnu }}}$ background prediction are summarized in Table 3 together with their typical size ranges across the search bins.

**Table 3:** Summary of systematic uncertainties in the ${\text {Z}} \rightarrow {{\upnu }} \bar{{{\upnu }}}$ background prediction, together with their typical size ranges across the search bins

Source	Range (%)
Limited size of data control samples	5–100
Limited size of MC samples	0–50
Lepton efficiency	0–5
Jet energy scale	0–5
Uncertainty in $R^{\mathrm {SF}/\mathrm {DF}}$	0–5
$M_{\mathrm {T2}}$ shape uncertainty (if $k_{{\text {Z}} \rightarrow {{\upnu }} \bar{{{\upnu }}}}\ne 1$)	0–40

#### Estimation of the multijet background

The background from SM events comprised uniquely of jets produced through the strong interaction (multijet events) is estimated from control regions in data selected using triggers that require $H_{\mathrm {T}}$ to exceed thresholds ranging from 125 (180) to 900 (1050)$\,\,\text {Ge}\text {V}$ in 2016 (2017–2018) data samples. In addition, events are required to have at least two jets with $p_{\mathrm {T}} > 10\,\,\text {Ge}\text {V}$.

The rebalance and smear (R&S) method used to estimate the multijet background consists of two steps. First, multijet data events are rebalanced by adjusting the $p_{\mathrm {T}}$ of the jets such that the resulting $p_{\mathrm {T}} ^\text {miss}$ is approximately zero. This rebalancing is performed through a likelihood maximization, accounting for the jet energy resolution [100, 101]. The output of the rebalancing step is an inclusive sample of multijet events with approximately zero $p_{\mathrm {T}} ^\text {miss}$ that are used as a seed for the second step, the smearing. In the smearing step, the $p_{\mathrm {T}}$ of the rebalanced jets is smeared according to the jet response function, in order to model the instrumental effects that lead to nonzero $p_{\mathrm {T}} ^\text {miss}$. The smearing step is repeated many times for each rebalanced event. The output of each smearing step is an independent sample of events, which serves to populate the tails of kinematic distributions such as $p_{\mathrm {T}} ^\text {miss}$ and $M_{\mathrm {T2}}$, and to obtain a more precise estimate of the multijet background than would be possible using only simulation.

The method makes use of jet response templates, i.e., distributions of the ratio of reconstructed jet $p_{\mathrm {T}}$ to generator-level jet $p_{\mathrm {T}}$. The templates are derived from simulation in bins of jet $p_{\mathrm {T}}$ and $\eta$, separately for b-tagged and non-b-tagged jets. Systematic uncertainties are assessed to cover for the modeling of the core and of the tails of the jet response templates.

Of all jets in the event, a jet qualifies for use in the R&S procedure if it has $p_{\mathrm {T}} >10\,\,\text {Ge}\text {V}$, and if it is not identified as a jet from pileup [131] in the case that $p_{\mathrm {T}} <100\,\,\text {Ge}\text {V}$. All other jets are left unchanged but are still used in the calculation of ${\vec p}_{\mathrm {T}}^{\text {miss}}$ and other jet-related quantities. An event with *n* qualifying jets is rebalanced by varying the $p_{\mathrm {T}} ^\text {reb}$ of each jet, which is an estimate of the true jet $p_{\mathrm {T}}$, to maximize the likelihood function

7 $$\begin{aligned} L = \prod _{i=1}^n \text {P} \left( p_{\text {T},i}^{\text {reco}} | p_{\text {T},i}^{\text {reb}} \right) \, G\left( \frac{p_{\text {T},\text {reb,x}}^\text {miss}}{\sigma _\text {T}^{\text {soft}}}\right) \, G\left( \frac{p_{\text {T},\text {reb,y}}^\text {miss}}{\sigma _\text {T}^{\text {soft}}}\right) , \end{aligned}$$

where

8 $$\begin{aligned} G(x) \equiv \mathrm {e}^{-x^2/2}, \end{aligned}$$

and

9 $$\begin{aligned} \vec {p}_{\text {T},\text {reb}}^{\text {miss}} \equiv {\vec p}_{\mathrm {T}}^{\text {miss}}- \sum _{i=1}^n \left( \vec {p}_{\text {T},i}^\text {reb} - \vec {p}_{\text {T},i}^\text {reco} \right) . \end{aligned}$$

The term $\text {P} ( p_{\text {T},i}^{\text {reco}} | p_{\text {T},i}^{\text {reb}} )$ in Eq. (7) is the probability for a jet with $p_{\mathrm {T}}$ of $p_{\text {T},i}^{\text {reb}}$ to be assigned a $p_{\mathrm {T}}$ of $p_{\text {T},i}^{\text {reco}}$ after reconstruction. This probability is taken directly from the jet response templates. The two *G*(*x*) terms in Eq. (7) enforce an approximate balancing condition. The $\vec {p}_{\text {T},\text {reb}}^{\text {miss}}$ terms in Eq. (7) represent the ${\vec p}_{\mathrm {T}}^{\text {miss}}$ after rebalancing, and are obtained by simply propagating the changes in jet $p_{\mathrm {T}}$ from rebalancing to ${\vec p}_{\mathrm {T}}^{\text {miss}}$. For the balancing of the *x* and *y* components of the ${\vec p}_{\mathrm {T}}^{\text {miss}}$, we use $\sigma _\text {T}^{\text {soft}}=20$ $\,\,\text {Ge}\text {V}$, which is approximately the width of the distributions of the *x* and *y* components of ${\vec p}_{\mathrm {T}}^{\text {miss}}$ in minimum bias events. This parameter represents the inherent missing energy due to low-$p_{\mathrm {T}}$ jets, unclustered energy, and jets from pileup that cannot be eliminated by rebalancing. A systematic uncertainty is assessed to cover for the effects of the variation of $\sigma _\text {T}^\text {soft}$.

The rebalanced events are used as input to the smearing procedure, where the $p_{\mathrm {T}}$ of each qualifying jet is rescaled by a random factor drawn from the corresponding jet response template, and all kinematic quantities are recalculated accordingly.

The background from multijet events is estimated by applying the signal region selection requirements to the above rebalanced and smeared sample, except events are only used if $p_{\text {T},\text {reb}}^\text {miss}<100\,\,\text {Ge}\text {V}$ to remove potential contamination from electroweak sources. This additional requirement is found to be fully efficient for multijet events, in simulation. Hence, no correction is applied to the prediction.

Systematic uncertainties are summarized in Table 4 together with their typical size ranges across the search bins.

**Table 4:** Summary of systematic uncertainties in the multijet background prediction, together with their typical size ranges across the search bins

Source	Range (%)
Jet energy resolution	10–20
Tails of jet response in templates	17–25
$\sigma _\text {T}^{\text {soft}}$ modeling	1–25
$N_{\mathrm {j}}$ modeling	1–19
$N_{{\text {b}}}$ modeling	1–16

**Fig. 3:**
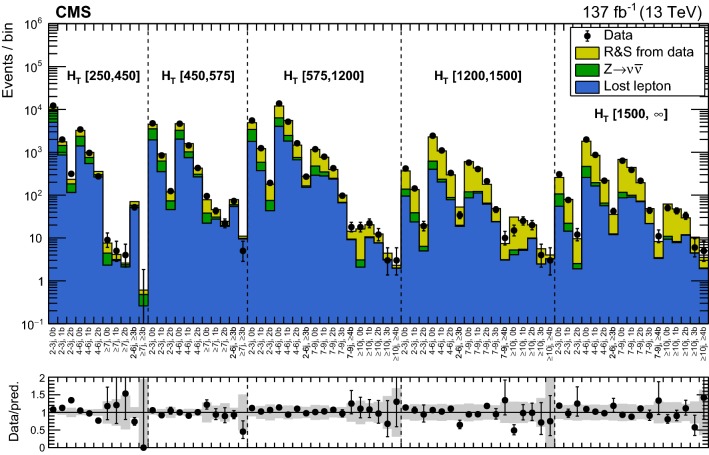
Validation of the R&S multijet background prediction in control regions in data selected with $\varDelta \phi _{\text {min}} <0.3$. Electroweak backgrounds (LL and ${\text {Z}} \rightarrow {{\upnu }} \bar{{{\upnu }}}$) are estimated from data. In regions where the amount of data is insufficient to estimate the electroweak backgrounds, the corresponding yields are taken directly from simulation. The bins on the horizontal axis correspond to the ($H_{\mathrm {T}}$, $N_{\mathrm {j}}$, $N_{{\text {b}}}$) topological regions. The gray band on the ratio plot represents the total uncertainty in the prediction

The resulting background prediction is validated in data using control regions enriched in multijet events. The results of the validation in a control region selected by inverting the $\varDelta \phi _{\text {min}}$ requirement are shown in Fig. 3. The electroweak backgrounds (LL and ${\text {Z}} \rightarrow {{\upnu }} \bar{{{\upnu }}}$) in this control region are estimated from data using transfer factors from leptonic control regions as described above. In regions where the number of events in the data leptonic control regions are insufficient, the electroweak background is taken from simulation. The observation is found to agree with the prediction, within the uncertainties.

### Search for disappearing tracks

In the search for disappearing tracks, the SM background consists of events with charged hadrons or leptons that interact in the tracker or are poorly reconstructed, as well as tracks built out of incorrect combinations of hits. The background is estimated from data, leveraging the orthogonal definition of STCs and selected STs (Sect. 3.2.2), as described by Eq. (10).

10 $$\begin{aligned} N_{\mathrm {ST}}^\text {est} = f_{\text {short}} \, N_{\mathrm {STC}}^\text {obs}, \end{aligned}$$

where $N_{\mathrm {ST}}$ is the number of selected short tracks, $N_{\mathrm {STC}}$ is the number of selected short track candidates, and $f_{\text {short}}$ is defined as:

11 $$\begin{aligned} f_{\text {short}} = N_{\mathrm {ST}}^\text {obs} / N_{\mathrm {STC}}^\text {obs}. \end{aligned}$$

The $f_{\text {short}}$ ratio is measured directly in data, in a control region of events selected using the same triggers and preselection criteria used for the signal regions, except the selection on $p_{\mathrm {T}} ^\text {miss}$ is relaxed to $p_{\mathrm {T}} ^\text {miss} >30\,\,\text {Ge}\text {V}$ for all $H_{\mathrm {T}}$ values, and the selection on $M_{\mathrm {T2}}$ is shifted to $60<M_{\mathrm {T2}} <100\,\,\text {Ge}\text {V}$. We exploit the empirical invariance of this ratio with respect to the $H_{\mathrm {T}}$ and $p_{\mathrm {T}} ^\text {miss}$ selection criteria, as observed in data control regions, to reduce the statistical uncertainty in the measurement. The $f_{\text {short}}$ ratio is therefore measured in data separately for each $N_{\mathrm {j}}$, track $p_{\mathrm {T}}$, track length category, and inclusively in $H_{\mathrm {T}}$. The $f_{\text {short}}$ values are measured separately in 2016 and 2017–2018 data, mainly to account for the upgrade of the CMS tracking detector after 2016. Since a reliable measurement in data of the $f_{\text {short}}$ ratio for long (L) tracks is not achievable because of the insufficient number of events, the value measured in data for medium (M) length tracks is used instead, after applying a correction based on simulation:

12 $$\begin{aligned} f_{\text {short}} (\text {L})_\text {data}^\text {est} = f_{\text {short}} (\text {M})_\text {data} \, f_{\text {short}} (\text {L})_{\mathrm {MC}}/f_{\text {short}} (\text {M})_{\mathrm {MC}}. \end{aligned}$$

A systematic uncertainty in the measured values of $f_{\text {short}}$ is assigned to cover for the empirically motivated assumption of its invariance with respect to $H_{\mathrm {T}}$ and $p_{\mathrm {T}} ^\text {miss}$. Its size is determined by varying the $H_{\mathrm {T}}$ and $p_{\mathrm {T}} ^\text {miss}$ selection requirements in data events with $60<M_{\mathrm {T2}} <100\,\,\text {Ge}\text {V}$. For long tracks, a conservative systematic uncertainty of 100% is assigned, as a correction based on simulation is used and there are insufficient data to study the effect of $H_{\mathrm {T}}$ and $p_{\mathrm {T}} ^\text {miss}$ variations.

The $f_{\text {short}}$ ratio is then used to predict the expected background in events with $M_{\mathrm {T2}} >100\,\,\text {Ge}\text {V}$, as described in Eq. (10).

In the presence of BSM physics, the above-defined control regions could be affected by signal contamination. Although the expected signal contamination is typically negligible, its potential impact is accounted for in the interpretation of the results, as further described in Sect. 6.

The background prediction is validated in data in an intermediate $M_{\mathrm {T2}}$ region ($100<M_{\mathrm {T2}} <200\,\,\text {Ge}\text {V}$). No excess event yield is observed. The event categorization in this validation region is identical to the signal region, allowing for a bin-by-bin validation of the background prediction.

**Fig. 4:**
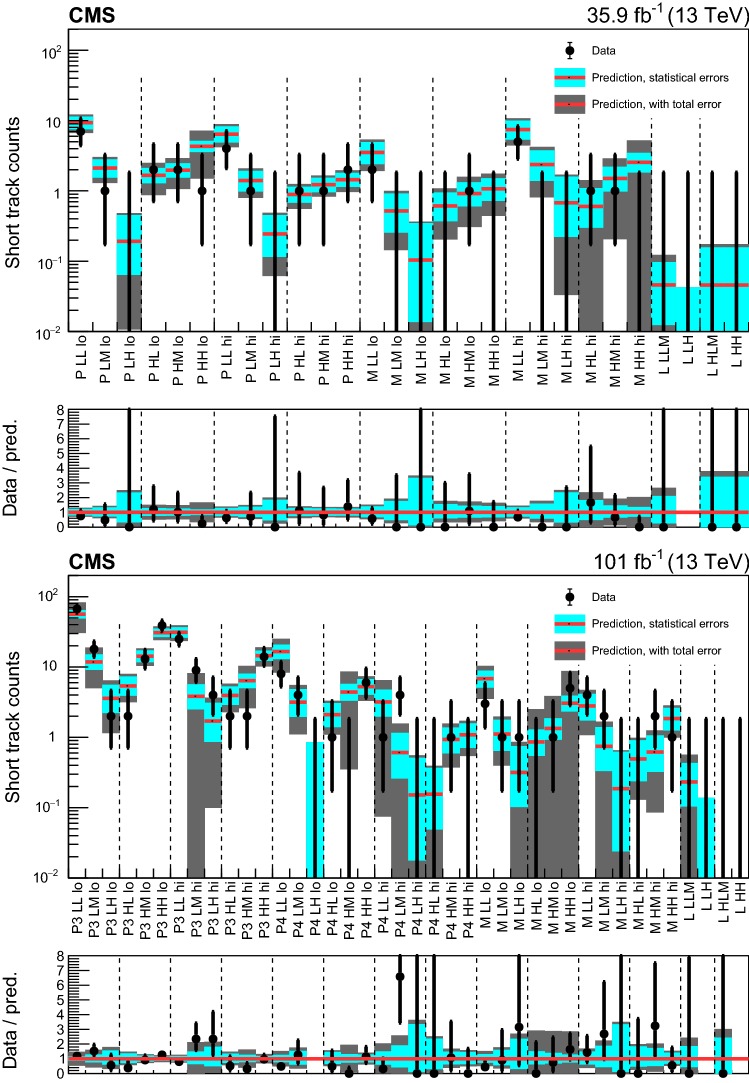
Validation of the background prediction method in (upper) 2016 and (lower) 2017–2018 data with $100<M_{\mathrm {T2}} <200\,\,\text {Ge}\text {V}$, for the disappearing tracks search. The red histograms represent the predicted backgrounds, while the black markers are the observed data counts. The cyan bands represent the statistical uncertainty in the prediction. The gray bands represent the total uncertainty in the prediction. The labels on the *x* axes are explained in Tables 24 and 25 of Appendix B.2. Regions whose predictions use the same measurement of $f_{\text {short}}$ are grouped by the vertical dashed lines. Bins with no entry in the ratio have zero predicted background

Figure 4 shows the result of the background prediction validation in 2016 data and in 2017–2018 data. We find good agreement between the observation and the background prediction in the validation region. An additional systematic uncertainty is assigned to cover for discrepancies exceeding statistical uncertainties. The uncertainties in the background prediction are summarized in Table 5 together with their typical size ranges across the search bins.

**Table 5:** Summary of systematic uncertainties in the disappearing track background prediction, together with their typical size ranges across the search bins. The systematic uncertainties arising from the assumption of kinematic invariance of $f_{\text {short}}$ and from the validation of the background prediction are always taken to be at least as large as the statistical uncertainties on the measured values of $f_{\text {short}}$ and on the background prediction in the validation region, respectively

Source	Range (%)
Limited size of data control samples	1–100
Limited size of data $f_{\text {short}}$ measurement samples	5–45
Kinematic invariance of $f_{\text {short}}$	10–80
Validation of background prediction	25–75

## Results

The data yields in the search regions are statistically compatible with the estimated backgrounds from SM processes.

### Inclusive $M_{\mathrm {T2}}$ search

**Fig. 5:**
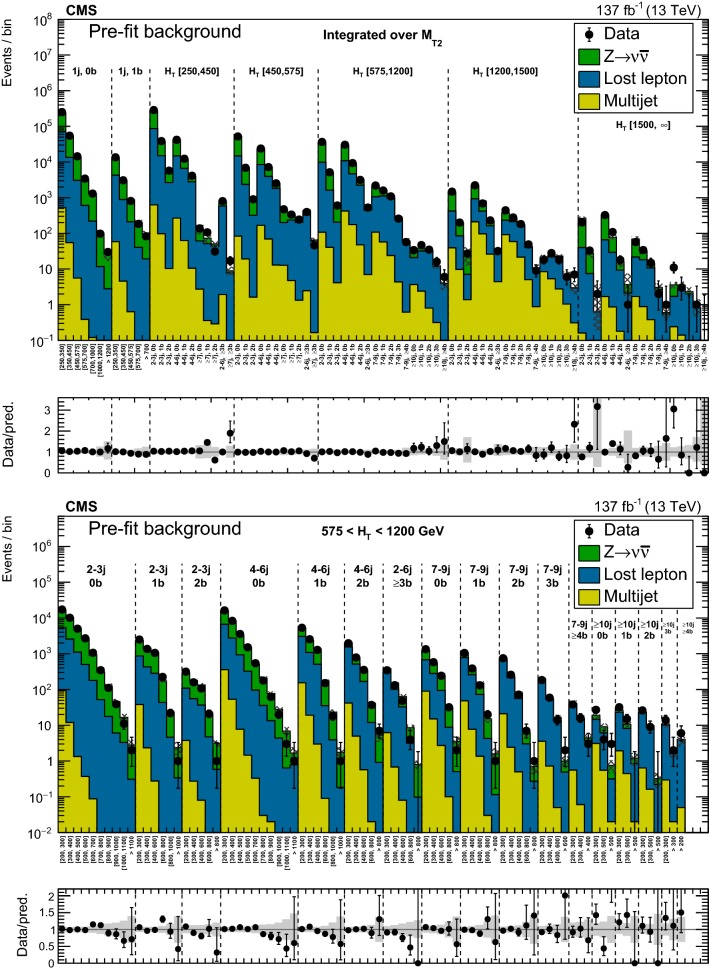
(Upper) Comparison of the estimated (pre-fit) background and observed data events in each topological region. The hatched bands represent the full uncertainty in the background estimate. The monojet regions ($N_{\mathrm {j}} = 1$) are identified by the labels “1j, 0b” and “1j, 1b”, and are binned in jet $p_{\mathrm {T}}$. The multijet regions are shown for each $H_{\mathrm {T}}$ region separately, and are labeled accordingly. The notations j, b are short for $N_{\mathrm {j}}$, $N_{{\text {b}}}$. (Lower) Same for individual $M_{\mathrm {T2}}$ search bins in the medium-$H_{\mathrm {T}}$ region. On the *x* axis, the $M_{\mathrm {T2}}$ binning is shown in units of GeV

A summary of the results of the $M_{\mathrm {T2}}$ inclusive search is shown in Fig. 5. Each bin in Fig. 5 (upper) corresponds to a single ($H_{\mathrm {T}}$, $N_{\mathrm {j}}$, $N_{{\text {b}}}$) topological region integrated over $M_{\mathrm {T2}}$. Figure 5 (lower) breaks down the background estimates and observed data yields into $M_{\mathrm {T2}}$ bins for the region $575< H_{\mathrm {T}} <1200\,\,\text {Ge}\text {V}$: each bin corresponds to a single $M_{\mathrm {T2}}$ bin, and vertical lines identify ($H_{\mathrm {T}}$, $N_{\mathrm {j}}$, $N_{{\text {b}}}$) topological regions. Distributions for the other $H_{\mathrm {T}}$ regions can be found in Figs. 23 and 24 in Appendix C.1. Background predictions and observed yields in all search regions are also summarized in Tables 12, 13, 14, 15, 16, 17, 18, 19, 20, 21, 22 and 23 in Appendix B.1. The background estimates and corresponding uncertainties rely exclusively on the inputs from control samples and simulation described in Sect. 4.1, prior to the fit to the data detailed in Sect. 6, and are referred to in the rest of the text as pre-fit background results.

To allow simpler reinterpretation, we also provide results for super signal regions, which cover subsets of the full analysis with simpler inclusive selection criteria and that can be used to obtain approximate interpretations of this search. The definitions of these regions are given in Table 6, with the predicted and observed number of events and the 95% confidence level ($\text {CL}$) upper limit on the number of signal events contributing to each region. Limits are set using a modified frequentist approach, employing the $\text {CL}_\text {s}$ criterion and relying on asymptotic approximations to calculate the distribution of the profile likelihood test-statistic used [132–135].

**Table 6:** Definitions of super signal regions, along with predictions, observed data, and the observed 95% $\text {CL}$ upper limits on the number of signal events contributing to each region ($N_{95}^{\mathrm {max}}$). The limits are shown as a range corresponding to an assumed uncertainty in the signal acceptance of 0 or 15% ($N_{95}^{\mathrm {max},0}$–$N_{95}^{\mathrm {max},15}$). A dash in the selection criteria means that no requirement is applied. All selection criteria as in the full analysis are applied. For regions with $N_{\mathrm {j}} =1$, $H_{\mathrm {T}} \equiv p_{\mathrm {T}} ^{\text {jet}}$. The mono-$\phi$ super signal region corresponds to the subset of analysis bins identified in Refs. [35, 36] as showing a significant excess in data based on the results of Ref. [9]

Region	$N_{\mathrm {j}}$	$N_{{\text {b}}}$	$H_{\mathrm {T}}$ ($\text {Ge}\text {V}$)	$M_{\mathrm {T2}}$ ($\text {Ge}\text {V}$)	Prediction	Data	$N_{95}^{\mathrm {max},0}$–$N_{95}^{\mathrm {max},15}$
2j loose	$\ge 2$	${-}$	$>1200$	$>1200$	$37\pm 14$	41	26.0${-}$27.2
2j tight	$\ge 2$	${-}$	$>1500$	$>1400$	$10.7^{+4.2}_{-4.1}$	13	11.7${-}$12.3
4j loose	$\ge 4$	${-}$	$>1200$	$>1000$	$54\pm 13$	72	41.5${-}$43.8
4j tight	$\ge 4$	${-}$	$>1500$	$>1400$	$6.4\pm 2.5$	10	10.9${-}$11.4
7j loose	$$\ge 7$$	$${-}$$	$$>1200$$	$$>600 $$	$$63^{+13}_{-12}$$	72	33.4$${-}$$35.0
7j tight	$$\ge 7$$	$${-}$$	$$>1500$$	$$>800 $$	$$14.9^{+4.3}_{-4.2}$$	14	10.1$${-}$$10.4
10j loose	$\ge 10$	${-}$	$>1200$	$>400$	$17.3\pm 4.0$	25	18.6${-}$19.5
10j tight	$\ge 10$	${-}$	$>1500$	$>600$	$3.6^{+1.2}_{-1.1}$	5	6.8${-}$7.1
2b loose	$\ge 2$	$\ge 2$	$>1200$	$>600$	$32.0\pm 4.5$	33	15.3${-}$15.9
2b tight	$\ge 2$	$\ge 2$	$>1500$	$>600$	$12.0^{+2.8}_{-2.7}$	12	9.1${-}$9.4
3b loose	$\ge 2$	$\ge 3$	$>1200$	$>400$	$17.6\pm 4.0$	16	10.0${-}$10.3
3b tight	$\ge 2$	$\ge 3$	$>1500$	$>400$	$7.5\pm 2.1$	5	5.3${-}$5.5
4b loose	$\ge 2$	$\ge 4$	$>1200$	$>400$	$2.1\pm 0.7$	2	4.2${-}$4.4
4b tight	$\ge 2$	$\ge 4$	$>1500$	$>400$	$0.8^{+0.4}_{-0.3}$	1	3.5${-}$3.6
7j 3b loose	$\ge 7$	$\ge 3$	$>1200$	$>400$	$10.9^{+3.0}_{-2.9}$	8	8.7${-}$8.9
7j 3b tight	$\ge 7$	$\ge 3$	$>1500$	$>400$	$4.6^{+2.0}_{-1.9}$	4	5.5${-}$5.7
7j 4b loose	$\ge 7$	$\ge 4$	$>1200$	$>400$	$1.7\pm 0.7$	2	4.3${-}$4.5
7j 4b tight	$\ge 7$	$\ge 4$	$>1500$	$>400$	$0.7\pm 0.4$	1	3.6${-}$3.7
10j 4b loose	$\ge 10$	$\ge 4$	$>1200$	$>400$	$0.6^{+0.5}_{-0.4}$	1	3.6${-}$3.7
10j 4b tight	$\ge 10$	$\ge 4$	$>1500$	$>400$	$0.1^{+0.5}_{-0.1}$	0	2.0${-}$2.1
Mono-$\phi$	1${-}$3	0	250${-}$450	200${-}$300	$(5.2\pm 0.3) \times 10^{5}$	$5.5\times 10^{5}$	(0.6${-}$0.8)$\times 10^{5}$
				(if $$N_{\mathrm {j}} \ge 2$$)			

### Search for disappearing tracks

**Fig. 6:**
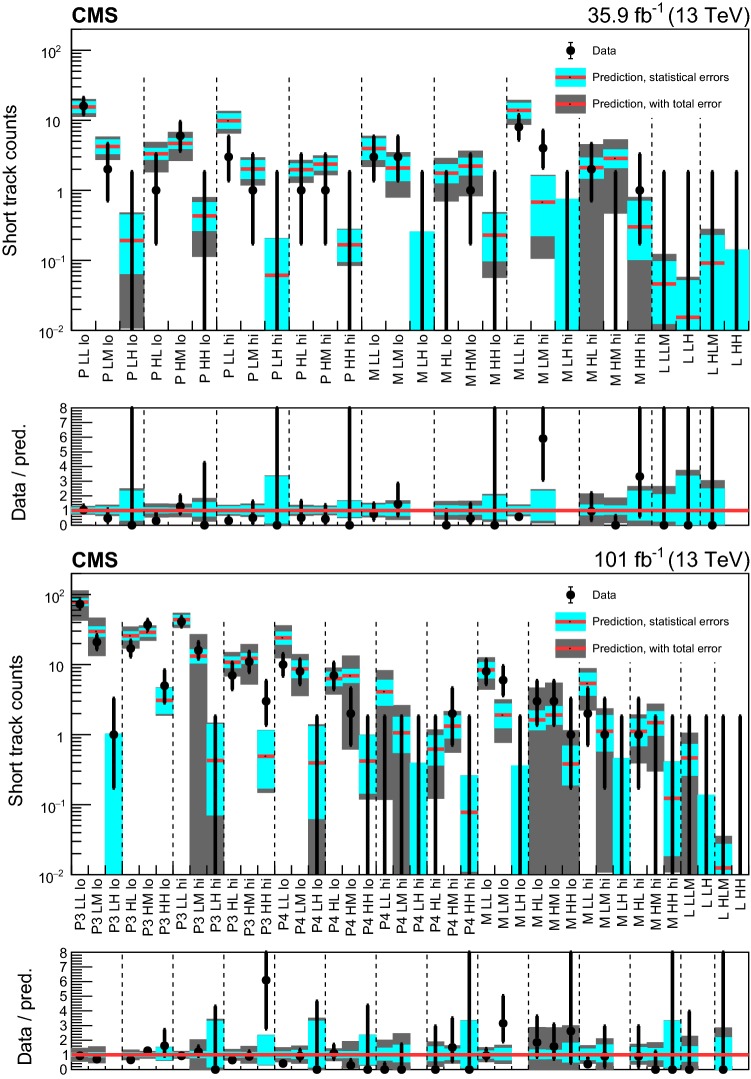
Comparison of the estimated (pre-fit) background and observed data events in (upper) each of the 2016 search regions, and in (lower) each of the 2017–2018 search regions, in the search for disappearing tracks. The red histogram represents the predicted background, while the black markers are the observed data counts. The cyan band represents the statistical uncertainty in the prediction. The gray band represents the total uncertainty. The labels on the *x* axes are explained in Tables 24 and 25 of Appendix B.2. Regions whose predictions use the same measurement of $f_{\text {short}}$ are grouped by the vertical dashed lines. Bins with no entry in the ratio have zero pre-fit predicted background

The results of the search for disappearing tracks are shown in Fig. 6. Just as in the case of the inclusive search, the background estimates and the uncertainties rely exclusively on the inputs from control samples and simulation (Sect. 4.2), prior to the fit to the data described in Sect. 6. We refer to them in the rest of the text as pre-fit background results. Background predictions and observed yields in all search regions are also summarized in Tables 24 and 25 in Appendix B.2.

## Interpretation of the results

The measurements are interpreted in the context of models of new physics. Maximum likelihood fits to the data in the signal regions are carried out under either background-only or background+signal hypotheses. The uncertainties in the modeling of the backgrounds, summarized in Sect. 4, are inputs to the fitting procedure. The likelihoods are constructed as the product of Poisson probability density functions, one for each signal region, with additional log-normal constraint terms that account for the uncertainties in the background estimates and, if considered, in the signal yields.

The background+signal fits are used to set 95% $\text {CL}$ upper limits on the cross sections for the signal models under consideration. These limits are then used, in conjunction with the theoretical cross section calculations, to exclude ranges of masses for the BSM particles of the signal models. Before the fits are performed, the signal yields are corrected to account for the expected signal contamination of the data control regions used to estimate the SM background.

For the interpretation of the results, simplified BSM physics models [21–25] are used. Simplified models are defined by sets of hypothetical particles and sequences of their production and decay. The theoretical parameters are thus reduced to a small number of masses and cross sections, providing an effective tool to characterize potential signals of BSM physics.

**Fig. 7:**
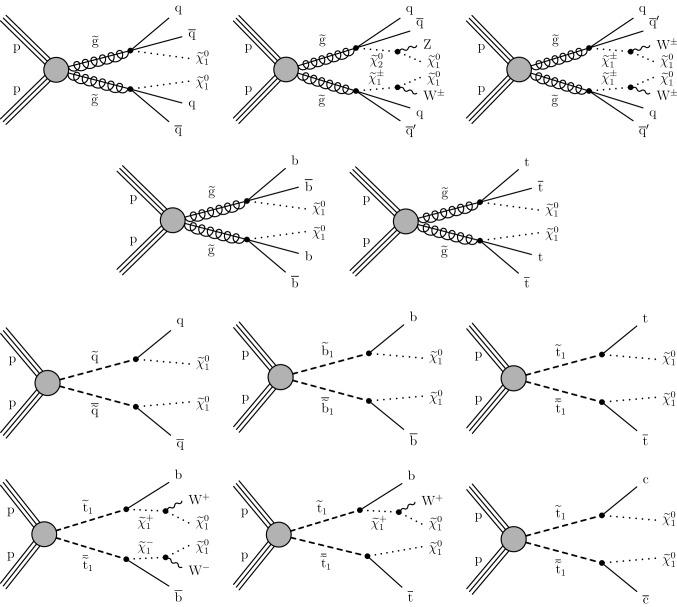
(Upper) Diagrams for three scenarios of direct gluino pair production where each gluino undergoes a three-body decay to light-flavor (u, d, s, c) quarks, with different decay modes. For mixed-decay scenarios, we assume equal branching fraction for each decay mode. (Upper middle) Diagrams for the direct gluino pair production where gluinos decay to bottom and top quarks. (Lower middle) Diagrams for the direct pair production of light-flavor, bottom, and top squark pairs. (Lower) Diagrams for three alternate scenarios of direct top squark pair production with different decay modes. For mixed-decay scenarios, we assume equal branching fraction for each decay mode

The results of the inclusive $M_{\mathrm {T2}}$ search are used to constrain each of the simplified models of SUSY shown in Fig. 7. For each scenario of gluino (squark) pair production, the simplified models assume that all SUSY particles other than those shown in the corresponding diagram are too heavy to be produced directly, and that the gluino (squark) decays promptly. The models assume that each gluino (squark) decays with a 100% branching fraction into the decay products depicted in Fig. 7. For models where the decays of the two gluinos or squarks in the same diagram differ, a 1/3 (1/2) branching fraction for each of the three (two) decay modes is assumed. In particular, for the diagram of gluino pair production where the decays of the two gluinos differ, each gluino can decay via a $\tilde{{\upchi }}_{2}^{0}$, $\tilde{{\upchi }}_{1}^{+}$, or $\tilde{{\upchi }}_{1}^{-}$. For scenarios with top squarks decaying into top quarks, the polarization of the top quark can be model dependent and a function of the top squark and neutralino mixing matrices. To maintain independence of any particular model realization, events are generated with unpolarized top quarks. Signal cross sections are calculated at approximately NNLO+NNLL (next-to-next-to-leading-logarithm) order in $\alpha _S$ [136–147]. For direct light-flavor squark pair production we assume either one single squark, or eight degenerate squarks ($\tilde{{\text {q}}}_{\mathrm{L}}{} +\tilde{{\text {q}}}_{\mathrm{R}}{}$, with $\tilde{{\text {q}}} =\tilde{{\text {u}}},~\tilde{{\text {d}}},~\tilde{{\text {s}}},~\tilde{{\text {c}}}$). For direct bottom and top squark pair production, we assume one single squark.

**Fig. 8:**
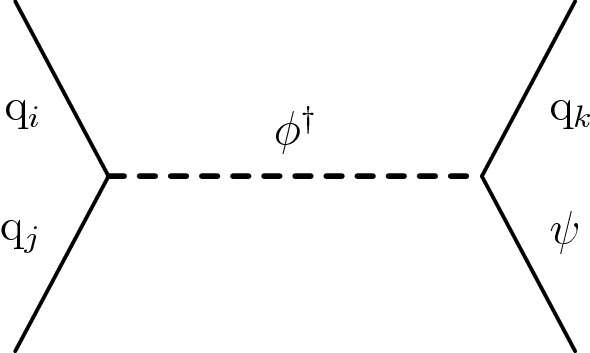
Diagram for the mono-$\phi$ model, where a colored scalar $\phi$ is resonantly produced, and it decays to an invisible massive Dirac fermion $\psi$ and an SM quark

The mono-$\phi$ model depicted in Fig. 8, that was recently proposed [35, 36] based on a reinterpretation of the results of Refs. [6–9, 37], is also probed by the inclusive $M_{\mathrm {T2}}$ search. In this case, the cross section for the signal is only calculated at LO order in $\alpha _S$.

**Fig. 9:**
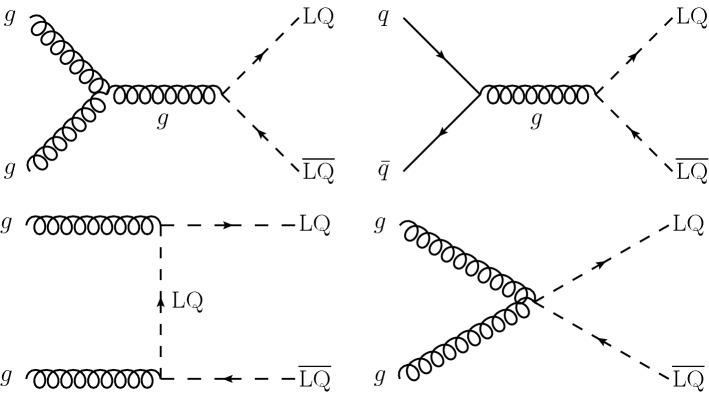
Diagrams for LQ pair production

Another interpretation of the inclusive $M_{\mathrm {T2}}$ results places cross section limits on LQ pair production (depicted in Fig. 9) as a function of the LQ mass, similarly to Ref. [11]. We consider production of either $\mathrm {LQ_{S}}$ or $\mathrm {LQ_{V}}$. In each case, we assume that only one LQ state is within mass reach of the LHC, and that the LQ decays with 100% branching fraction to a neutrino and a single type of quark: a light-flavor quark (q $=$ u, d, s, or c), a bottom quark, or a top quark. The cross sections for $\mathrm {LQ_{S}}$ ($\mathrm {LQ_{V}}$) pair production are computed to NLO (LO) order in $\alpha _S$ following Ref. [55]. The $\mathrm {LQ_{S}}$ pair production cross section depends only on the LQ mass. For $\mathrm {LQ_{V}}$, additional constraints are imposed by unitarity at high energy scales, leading to model dependent solutions and thus production cross sections. In the model of Ref. [55], developed to explain the flavor physics anomalies, the additional relevant parameter for the $\mathrm {LQ_{V}}$ pair production cross section is $\kappa$, a dimensionless coupling that is 1 in the Yang–Mills case and 0 in the minimal coupling case. We consider both values. For $\kappa =1$, the cross section for the $\mathrm {LQ_{V}}$ pair production is a factor 5–20 times larger than that of $\mathrm {LQ_{S}}$, depending on the LQ mass. In the $\mathrm {LQ_{V}}$ model, other free parameters are $g_{{\text {t}} _{\text {L}}}$ and $g_{{\text {b}} _{\text {L}}}$, the couplings of the $\mathrm {LQ_{V}}$ to ${\text {t}} {{\upnu }}$ and ${\text {b}} {\uptau }$ pairs, respectively. However, $g_{{\text {t}} _{\text {L}}}$ and $g_{{\text {b}} _{\text {L}}}$ do not affect the cross section or the kinematics for the $\mathrm {LQ_{V}}$ pair production, and we assume $g_{{\text {t}} _{\text {L}}} = g_{{\text {b}} _{\text {L}}} = 0.1$, as predicted to explain the flavor physics anomalies.

**Fig. 10:**
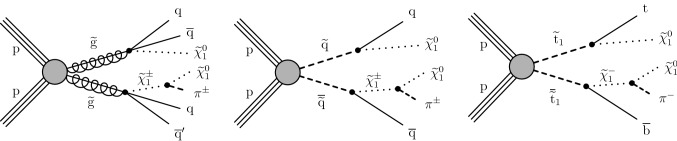
Diagrams for direct (left) gluino, (middle) light-flavor (u, d, s, c) squark, and (right) top squark pair production, where the directly produced gluinos and squarks can decay via a long-lived ${\tilde{{\upchi }}_{1}^{\pm }}$. For gluinos, we assume a 1/3 decay branching fraction to each $\tilde{{\upchi }}_{1}^{0}$, $\tilde{{\upchi }}_{1}^{+}$, and $\tilde{{\upchi }}_{1}^{-}$, and each gluino decays to light-flavor quarks. For squarks, we assume a 1/2 branching fraction for decays to $\tilde{{\upchi }}_{1}^{0}$ and to the ${\tilde{{\upchi }}_{1}^{\pm }}$ allowed by charge conservation. The mass of the ${\tilde{{\upchi }}_{1}^{\pm }}$ is larger than the mass of the $\tilde{{\upchi }}_{1}^{0}$ by hundreds of $\,\,\text {Me}\text {V}$. The ${\tilde{{\upchi }}_{1}^{\pm }}$ decays to a $\tilde{{\upchi }}_{1}^{0}$ via a pion, which is too soft to be detected

The results of the search for disappearing tracks are used to constrain simplified models of SUSY where gluinos and squarks are produced in pairs, and each one decays either directly to the lightest neutralino ($\tilde{{\upchi }}_{1}^{0}$), or first to a long-lived chargino (${\tilde{{\upchi }}_{1}^{\pm }}$) as shown in Fig. 10. All possible decays are assumed to occur with equal probability. Thus, the gluino branching fraction is 1/3 each for the decay to $\tilde{{\upchi }}_{1}^{0}$, $\tilde{{\upchi }}_{1}^{+}$, and $\tilde{{\upchi }}_{1}^{-}$, and the squark branching fraction is 1/2 to $\tilde{{\upchi }}_{1}^{0}$ and 1/2 to the ${\tilde{{\upchi }}_{1}^{\pm }}$ of opposite charge. The ${\tilde{{\upchi }}_{1}^{\pm }}$ and $\tilde{{\upchi }}_{1}^{0}$ are assumed to be wino-like, and their masses to differ by a few hundred MeV [13, 14]. Thus, the phase space for the decay of the ${\tilde{{\upchi }}_{1}^{\pm }}$ to a $\tilde{{\upchi }}_{1}^{0}$ and a charged pion is small. As a consequence, the ${\tilde{{\upchi }}_{1}^{\pm }}$ has lifetime of the order of a few nanoseconds, and the momentum of the pion originating from its decay does not exceed a few hundred MeV. Hence, the final state shows negligible dependence on small variations of the mass difference between ${\tilde{{\upchi }}_{1}^{\pm }}$ and $\tilde{{\upchi }}_{1}^{0}$. Lifetimes of the ${\tilde{{\upchi }}_{1}^{\pm }}$ are probed in the range $c\tau _{0}({\tilde{{\upchi }}_{1}^{\pm }}) =$ 1–2000$\,\text {cm}$.

Uncertainties in the signal yield for the simplified models considered are listed in Table 7. The sources of uncertainty and the methods used to evaluate their effect on the interpretation are the same as those discussed in Refs. [9, 96]. For each data sample corresponding to the different periods of data taking (2016, 2017, and 2018), uncertainties in the luminosity measurement [148–150], ISR modeling, fast simulation $p_{\mathrm {T}} ^\text {miss}$ distributions, and b tagging and lepton efficiencies are treated as correlated across search bins. Uncertainties in fast simulation $p_{\mathrm {T}} ^\text {miss}$ distributions, b tagging, and lepton efficiencies are treated as correlated also across data samples. The remaining uncertainties are taken as uncorrelated. In the search for disappearing tracks, all other tagging and lepton efficiencies are neglected. Other uncertainties associated with the modeling of disappearing tracks are treated as correlated across search bins. Specifically, an uncertainty in the signal yield is assigned, equal to one half of the track selection inefficiency: 25 (17.5)% for P (M and L) tracks in 2016, and 10% for tracks of all lengths in 2017–2018. Additionally, a 6% uncertainty in the 2017–2018 signal yield is assigned to account for inaccuracies in the fast simulation modeling of the signal acceptance.

**Table 7:** Systematic uncertainties in the signal yields for the simplified models of BSM physics. The large statistical uncertainties in the simulated signal sample come from a small number of bins with low acceptance, which are typically not among the most sensitive bins contributing to a given model benchmark point

Source	Range (%)
Integrated luminosity	2.3–2.5
Limited size of MC samples	1–100
b tagging efficiency, heavy flavors	0–40
b tagging efficiency, light flavors	0–20
Lepton efficiency	0–20
Jet energy scale	5
Fast simulation $p_{\mathrm {T}} ^\text {miss}$ modeling	0–5
ISR modeling	0–30
$\mu _{\mathrm {R}}$ and $\mu _{\mathrm {F}}$	5

### Inclusive $M_{\mathrm {T2}}$ search

**Fig. 11:**
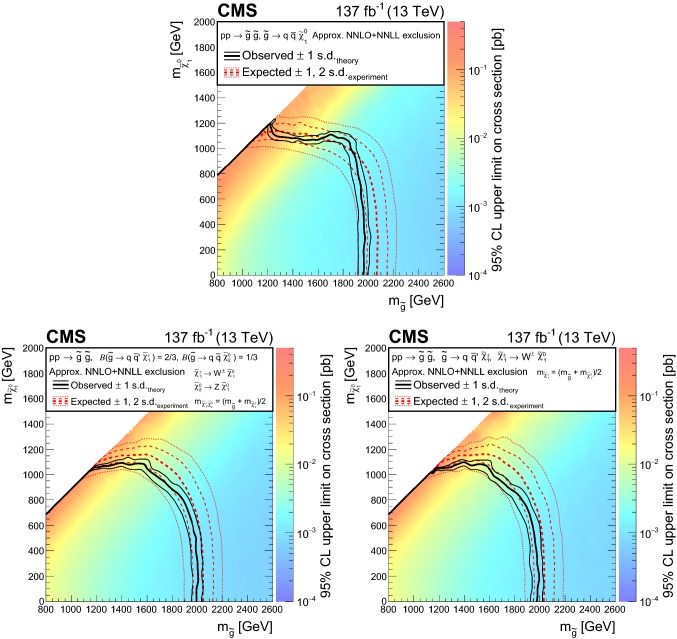
Exclusion limits at 95% $\text {CL}$ for direct gluino pair production, where (upper) ${\tilde{{\text {g}}}} \rightarrow {\text {q}} {\bar{{{\text {q}}}}} \tilde{{\upchi }}_{1}^{0}$, (lower left) ${\tilde{{\text {g}}}} \rightarrow {\text {q}} {\bar{{{\text {q}}}}} {\tilde{{\upchi }}_{2}^{0}}$ and ${\tilde{{\upchi }}_{2}^{0}}\rightarrow {\text {Z}} \tilde{{\upchi }}_{1}^{0}$, or ${\tilde{{\text {g}}}} \rightarrow {\text {q}} {\bar{{{\text {q}}}}} ' {\tilde{{\upchi }}_{1}^{\pm }}$ and ${\tilde{{\upchi }}_{1}^{\pm }} \rightarrow \hbox {W}^{\pm }\tilde{{\upchi }}_{1}^{0}$, and (lower right) ${\tilde{{\text {g}}}} \rightarrow {\text {q}} {\bar{{{\text {q}}}}} ' {\tilde{{\upchi }}_{1}^{\pm }}$ and ${\tilde{{\upchi }}_{1}^{\pm }} \rightarrow \hbox {W}^{\pm }\tilde{{\upchi }}_{1}^{0}$ (with q $=$ u, d, s, or c). For the scenarios where the gluinos decay via an intermediate $\tilde{{\upchi }}_{2}^{0}$or ${\tilde{{\upchi }}_{1}^{\pm }}$, $\tilde{{\upchi }}_{2}^{0}$and ${\tilde{{\upchi }}_{1}^{\pm }}$ are assumed to be mass-degenerate, with $m_{{\tilde{{\upchi }}_{1}^{\pm }},~{\tilde{{\upchi }}_{2}^{0}}}=0.5(m_{{\tilde{{\text {g}}}}}+m_{\tilde{{\upchi }}_{1}^{0}})$. The area enclosed by the thick black curve represents the observed exclusion region, while the dashed red lines indicate the expected limits and their ±1 and ±2 standard deviation (s.d.) ranges. The thin black lines show the effect of the theoretical uncertainties in the signal cross section. Signal cross sections are calculated at approximately NNLO+NNLL order in $\alpha _S$ [136–147], assuming 1/3 branching fraction (${\mathcal {B}}$) for each decay mode in the mixed-decay scenarios, or unity branching fraction for the indicated decay

**Fig. 12:**
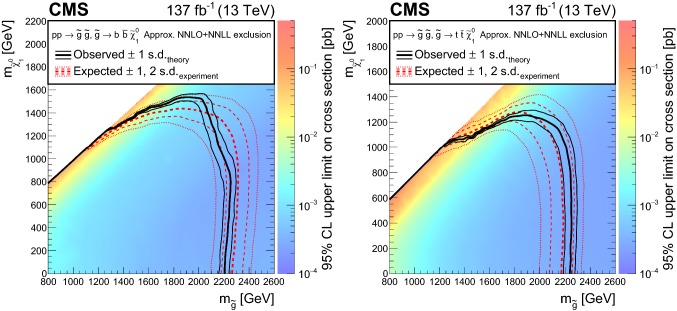
Exclusion limits at 95% $\text {CL}$ for direct gluino pair production where the gluinos decay to (left) bottom quarks and (right) top quarks. The area enclosed by the thick black curve represents the observed exclusion region, while the dashed red lines indicate the expected limits and their ±1 and ±2 standard deviation (s.d.) ranges. The thin black lines show the effect of the theoretical uncertainties in the signal cross section. Signal cross sections are calculated at approximately NNLO+NNLL order in $\alpha _S$ [136–147], assuming unity branching fraction for the indicated decay

**Fig. 13:**
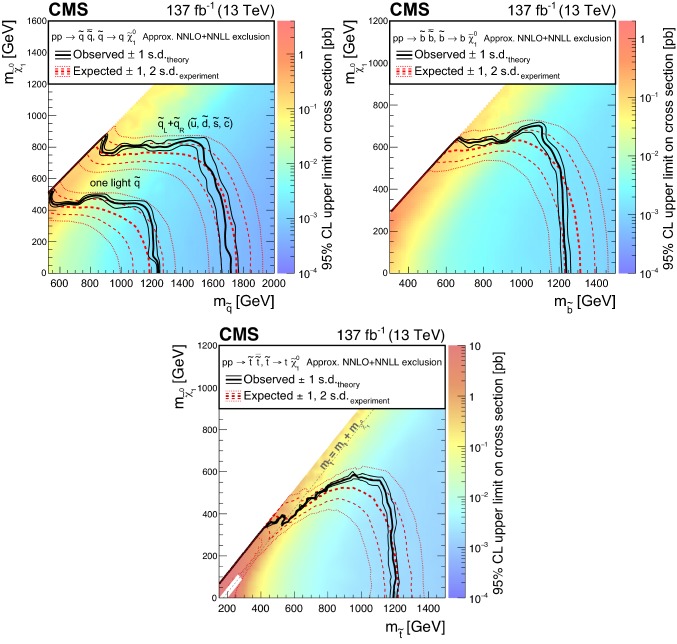
Exclusion limit at 95% $\text {CL}$ for (upper left) light-flavor squark pair production, (upper right) bottom squark pair production, and (lower) top squark pair production. The area enclosed by the thick black curve represents the observed exclusion region, while the dashed red lines indicate the expected limits and their ±1 and ±2 standard deviation (s.d.) ranges. The thin black lines show the effect of the theoretical uncertainties in the signal cross section. The white diagonal band in the top squark pair production exclusion limit corresponds to the region $|m_{\tilde{{\text {t}}}}-m_{{\text {t}}}-m_{\tilde{{\upchi }}_{1}^{0}} |< 25\,\,\text {Ge}\text {V}$ and small $m_{\tilde{{\upchi }}_{1}^{0}}$. Here the efficiency of the selection is a strong function of $m_{\tilde{{\text {t}}}}-m_{\tilde{{\upchi }}_{1}^{0}}$, and as a result the precise determination of the cross section upper limit is uncertain because of the finite granularity of the available MC samples in this region of the ($m_{\tilde{{\text {t}}}}, m_{\tilde{{\upchi }}_{1}^{0}}$) plane. In the same exclusion limit, the dashed black diagonal line corresponds to $m_{\tilde{{\text {t}}}}=m_{{\text {t}}}+m_{\tilde{{\upchi }}_{1}^{0}}$. Signal cross sections are calculated at approximately NNLO+NNLL order in $\alpha _S$ [136–147], assuming unity branching fraction for the indicated decay

**Fig. 14:**
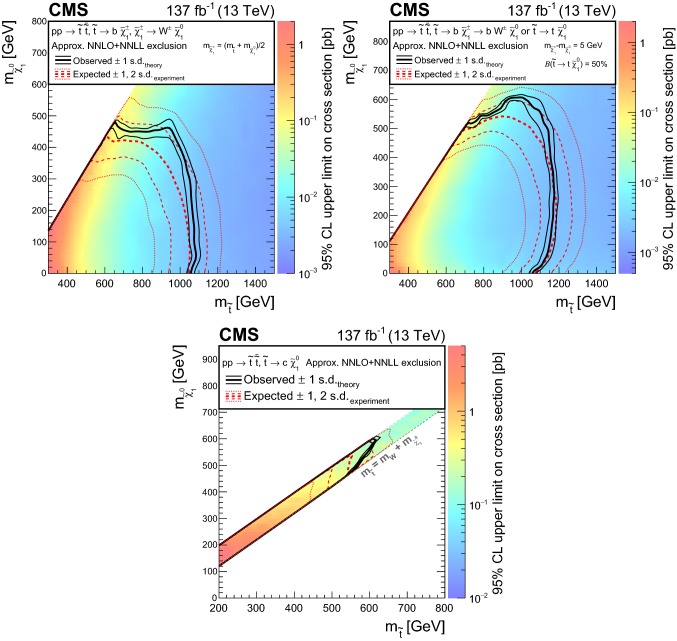
Exclusion limit at 95% $\text {CL}$ for top squark pair production for different decay modes of the top squark. (Upper left) For the scenario where ${\text {p}} {\text {p}} \rightarrow \tilde{{\text {t}}} \bar{\tilde{{\text {t}}}} \rightarrow {\text {b}} {\bar{{{\text {b}}}}} {\tilde{{\upchi }}_{1}^{\pm }} {{\upchi }}_{1}^{\mp }$, ${\tilde{{\upchi }}_{1}^{\pm }} \rightarrow {\text {W}} ^{\pm } \tilde{{\upchi }}_{1}^{0}$, the mass of the chargino is chosen to be half way in between the masses of the top squark and the neutralino. (Upper right) A mixed-decay scenario, ${\text {p}} {\text {p}} \rightarrow \tilde{{\text {t}}} \bar{\tilde{{\text {t}}}}$ with equal branching fractions for the top squark decays $\tilde{{\text {t}}} \rightarrow {\text {t}} \tilde{{\upchi }}_{1}^{0}$ and $\tilde{{\text {t}}} \rightarrow {\text {b}} \tilde{{\upchi }}_{1}^{+}$, $\tilde{{\upchi }}_{1}^{+} \rightarrow {\text {W}} ^{*+}\tilde{{\upchi }}_{1}^{0}$, is also considered, with the chargino mass chosen such that $\varDelta m\left( {\tilde{{\upchi }}_{1}^{\pm }},\tilde{{\upchi }}_{1}^{0} \right) = 5\,\,\text {Ge}\text {V}$. (Lower) Finally, we also consider a compressed spectrum scenario where ${\text {p}} {\text {p}} \rightarrow \tilde{{\text {t}}} \bar{\tilde{{\text {t}}}} \rightarrow {\text {c}} {\bar{{{\text {c}}}}} \tilde{{\upchi }}_{1}^{0} \tilde{{\upchi }}_{1}^{0}$. In this scenario, mass ranges are considered where the $\tilde{{\text {t}}} \rightarrow {\text {c}} \tilde{{\upchi }}_{1}^{0}$ branching fraction can be significant. The area enclosed by the thick black curve represents the observed exclusion region, while the dashed red lines indicate the expected limits and their ±1 and ±2 standard deviation (s.d.) ranges. The thin black lines show the effect of the theoretical uncertainties in the signal cross section. Signal cross sections are calculated at approximately NNLO+NNLL order in $\alpha _S$ [136–147], assuming 50% branching fraction (${\mathcal {B}}$) for each decay mode in the mixed-decay scenarios, or unity branching fraction for the indicated decay

Figure 11 shows the exclusion limits at 95% $\text {CL}$ for direct gluino pair production where the gluinos decay to light-flavor quarks under three different decay scenarios. Exclusion limits for direct gluino pair production where the gluinos decay to bottom and top quarks are shown in Fig. 12, and those for the direct production of squark pairs are shown in Fig. 13. Three alternate decay scenarios are also considered for the direct pair production of top squarks, and their exclusion limits are shown in Fig. 14.

Table 8 summarizes the limits on the masses of SUSY particles excluded for the simplified model scenarios considered. These results extend the constraints on gluino and squark masses by about 100–350$\,\,\text {Ge}\text {V}$ and on the $\tilde{{\upchi }}_{1}^{0}$ mass by 100–250$\,\,\text {Ge}\text {V}$ with respect to the limits in Ref. [9].

**Table 8:** Summary of the observed 95% $\text {CL}$ exclusion limits on the masses of SUSY particles for different simplified model scenarios. The highest limits on the mass of the directly produced particles and on the mass of the $\tilde{{\upchi }}_{1}^{0}$ are quoted

Simplified model	Highest limit on directly produced SUSY particle mass ($\text {Ge}\text {V}$)	Highest limit on $\tilde{{\upchi }}_{1}^{0}$ mass ($\text {Ge}\text {V}$)
*Direct gluino pair production*		
${\tilde{{\text {g}}}} \rightarrow {\text {q}} {\bar{{{\text {q}}}}} \tilde{{\upchi }}_{1}^{0}$	1970	1200
${\tilde{{\text {g}}}} \rightarrow {\text {q}} {\bar{{{\text {q}}}}} {\text {Z}} \tilde{{\upchi }}_{1}^{0}$ or ${\tilde{{\text {g}}}} \rightarrow {\text {q}} {\bar{{{\text {q}}}}} ' \hbox {W}^{\pm }\tilde{{\upchi }}_{1}^{0}$	2020	1090
${\tilde{{\text {g}}}} \rightarrow {\text {b}} {}{\bar{{{\text {b}}}}} \tilde{{\upchi }}_{1}^{0}$	2250	1525
${\tilde{{\text {g}}}} \rightarrow {{\text {t}} {\bar{{{\text {t}}}}}} \tilde{{\upchi }}_{1}^{0}$	2250	1250
*Direct squark pair production*		
Eight degenerate light squarks	1710	870
Single light squark	1250	525
Bottom squark	1240	700
Top squark	1200	580

**Fig. 15:**
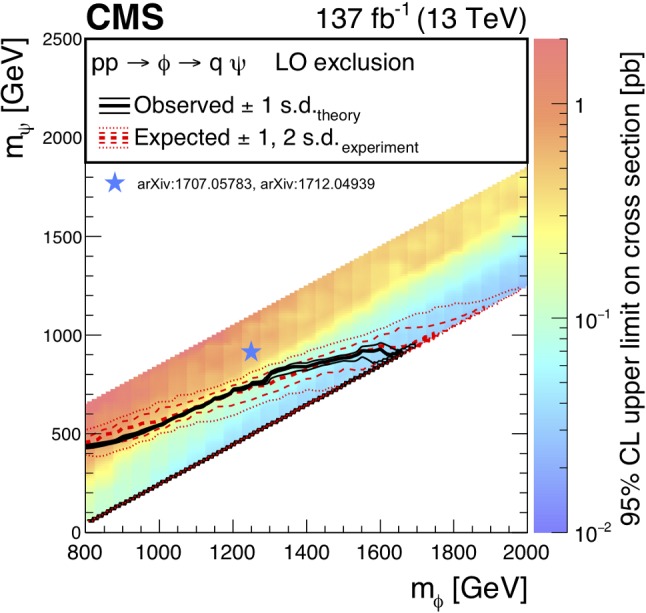
Exclusion limit at 95% $\text {CL}$ for the mono-$\phi$ model. We consider the mass range where such a model could be interesting based on a reinterpretation of previous analyses [35, 36]. The area enclosed by the thick black curve represents the observed exclusion region, while the dashed red lines indicate the expected limits and their ±1 and ±2 standard deviation (s.d.) ranges. The thin black lines show the effect of the theoretical uncertainties in the signal cross section. The blue star at $\left( m_{\phi },~m_{\psi }\right) =\left( 1250,~900\right) \,\,\text {Ge}\text {V}$ indicates the best fit mass point reported in Refs. [35, 36]. Signal cross sections are calculated at LO order in $\alpha _S$

Figure 15 shows the exclusion limits for the mono-$\phi$ model [35, 36]. Based on the LO cross section calculation, we obtain mass limits as large as 1660 and 925$\,\,\text {Ge}\text {V}$ on $m_{\phi }$ and on $m_{\psi }$, respectively. In this model, the analysis of Refs. [35, 36] reports best fit parameters $\left( m_{\phi },~m_{\psi }\right) =\left( 1250,~900\right) \,\,\text {Ge}\text {V}$ and product of the cross section and branching fraction of about 0.3$\,\,\text {pb}$. For this mass point, we find a modest (1.1 standard deviations) excess, and we set an upper limit on the product of the cross section and branching fraction of about 0.6 (0.4 expected)$\,\,\text {pb}$, equal to 4.7 (3.2) times the assumed LO theoretical cross section.

**Fig. 16:**
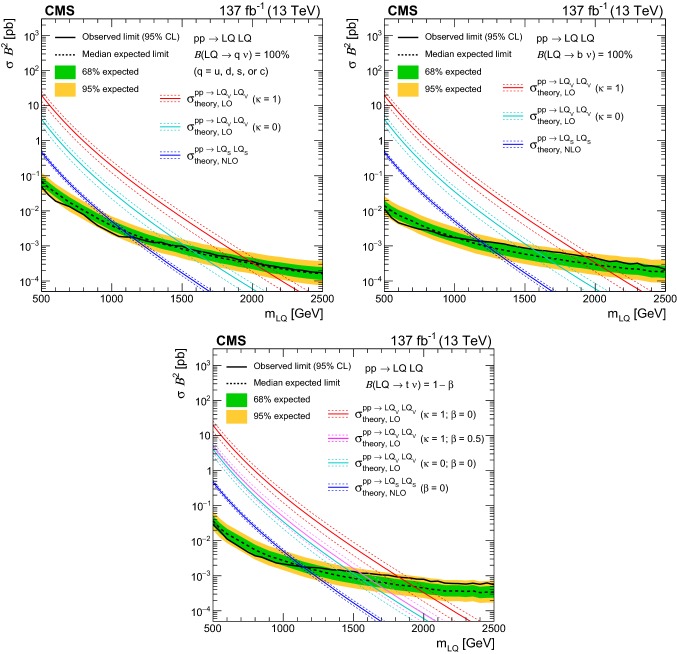
The 95% $\text {CL}$ upper limits on the production cross sections as a function of LQ mass for LQ pair production decaying with 100% branching fraction (${\mathcal {B}}$) to a neutrino and (upper left) a light quark (one of u, d, s, or c), (upper right) a bottom quark, or (lower) a top quark. The solid (dashed) black line represents the observed (median expected) exclusion. The inner green (outer yellow) band indicates the region containing 68 (95)% of the distribution of limits expected under the background-only hypothesis. The dark blue lines show the theoretical cross section for $\mathrm {LQ}_{\mathrm {S}}$ pair production with its uncertainty. The red (light blue) lines show the same for $\mathrm {LQ}_{\mathrm {V}}$ pair production assuming $\kappa = 1$ (0). (Lower) Also shown in magenta is the product of the theoretical cross section and the square of the branching fraction ($\sigma {\mathcal {B}}^{2}$), for vector LQ pair production assuming $\kappa = 1$ and a 50% branching fraction to ${\text {t}} {{{{\upnu }}}_{{\uptau }}{}}$, with the remaining 50% to ${\text {b}} {\uptau }$. Signal cross sections are calculated at NLO (LO) in $\alpha _S$ for scalar (vector) LQ pair production

The LQ limits from the $M_{\mathrm {T2}}$ search are shown in Fig. 16, where only one LQ state is assumed to be within reach of the LHC, and where each LQ is assumed to decay to a neutrino and a single type of quark.

In Refs. [54, 55], a model is proposed as a coherent explanation of the flavor physics anomalies. It is based on an $\mathrm {LQ_{V}}$ that can decay to ${\text {t}} {{\upnu }}$ and to ${\text {b}} {\uptau }$ final states, each with 50% branching fraction. In our analysis, events are selected with a charged-lepton veto, including hadronically decaying $\uptau$ leptons. Hence, only the 25% of events where both LQs decay to ${\text {t}} {{\upnu }}$ are considered to set constraints on this model, and the theoretical prediction for this branching fraction is shown as a separate curve in Fig. 16 (lower).

Table 9 summarizes the limits on the masses of the LQs excluded for the considered scenarios. These results extend the constraints on LQ masses by up to about 200$\,\,\text {Ge}\text {V}$ with respect to the limits of Ref. [11], providing the most stringent constraint to date in models of LQ pair production.

**Table 9:** Summary of the observed 95% $\text {CL}$ exclusion limits on the masses of LQs for the considered scenarios. The columns show scalar or vector LQ with the choice of $\kappa$, while the rows show the LQ decay channel. For mixed-decay scenarios, the assumed branching fractions (${\mathcal {B}}$) are indicated

	$\mathrm {LQ_{S}}$	$\mathrm {LQ_{V}},~\kappa =1$	$\mathrm {LQ_{V}},~\kappa =0$
	Mass ($\text {Ge}\text {V}$)	Mass ($\text {Ge}\text {V}$)	Mass ($\text {Ge}\text {V}$)
$\mathrm {LQ}\rightarrow {\text {q}} {{\upnu }}$ (q $=$ u, d, s, or c)	1140	1980	1560
$\mathrm {LQ}\rightarrow {\text {b}} {{\upnu }}$	1185	1925	1560
$\mathrm {LQ}\rightarrow {\text {t}} {{\upnu }}$	1140	1825	1475
$$\mathrm {LQ}\rightarrow \left\{ \begin{array}{l} {\text {t}} {{\upnu }} ~\left( {\mathcal {B}}=50\%\right) \\ {\text {b}} {\uptau } ~\left( {\mathcal {B}}=50\%\right) \end{array}\right. $$	–	1550	1225

The 95% $\text {CL}$ upper limits on signal cross sections obtained using the most sensitive super signal regions of Table 6 are typically less stringent by a factor of $\sim 1.5{-}3$ compared to those obtained in the fully binned analysis. This difference in performance arises from the larger signal acceptance of the full analysis, as well as from the more favorable signal-to-background ratio achieved in its individual bins, compared to the super signal regions.

### Search for disappearing tracks

**Fig. 17:**
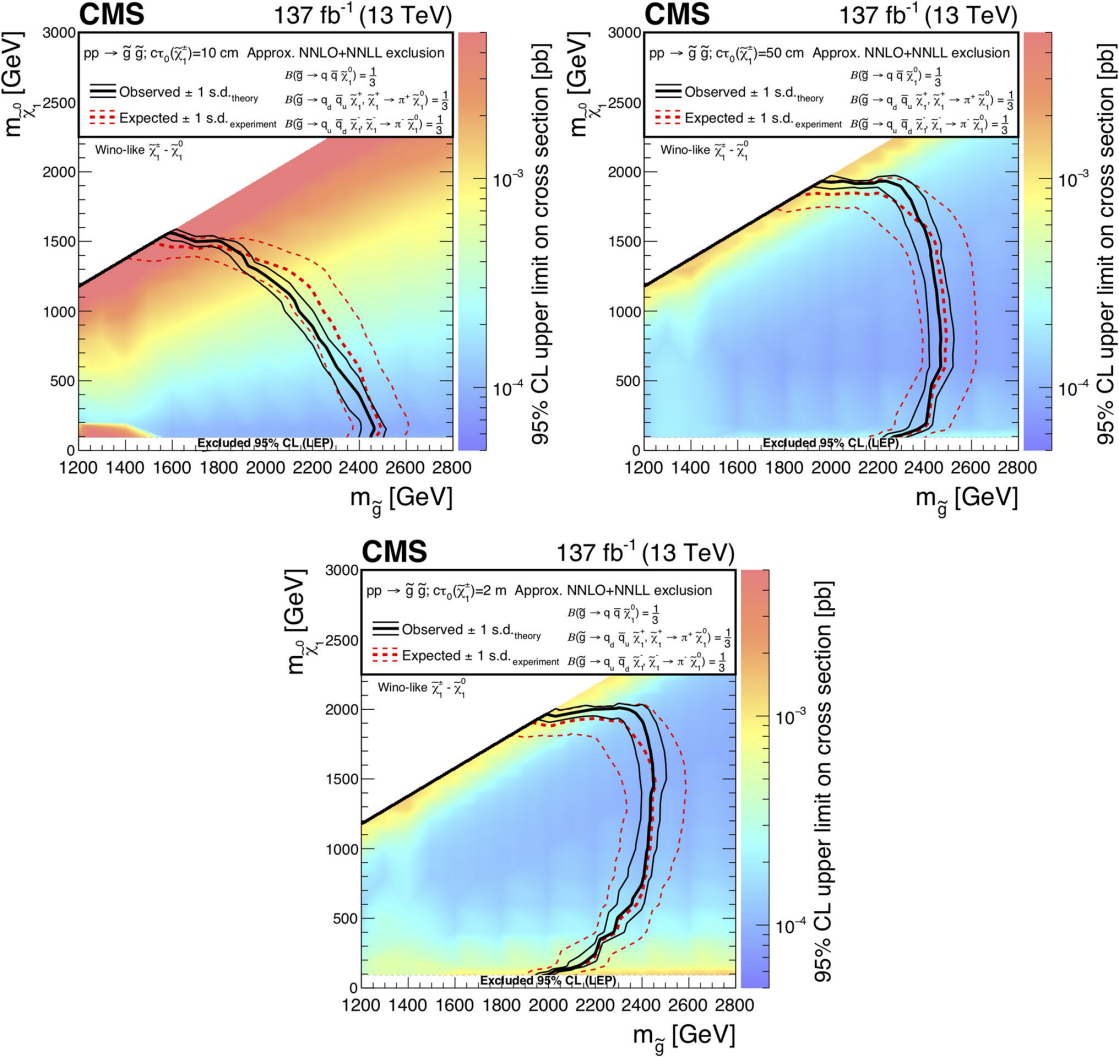
Exclusion limits at 95% $\text {CL}$ for direct gluino pair production where the gluinos decay to light-flavor (u, d, s, c) quarks, with $c\tau _{0}({\tilde{{\upchi }}_{1}^{\pm }}) =$ (upper left) 10$\,\text {cm}$, (upper right) 50$\,\text {cm}$, and (lower) 200$\,\text {cm}$. The area enclosed by the thick black curve represents the observed exclusion region, while the dashed red lines indicate the expected limits and their ±1 standard deviation (s.d.) ranges. The thin black lines show the effect of the theoretical uncertainties in the signal cross section. The white band for masses of the $\tilde{{\upchi }}_{1}^{0}$ below 91.9$\,\,\text {Ge}\text {V}$ represents the region of the mass plane excluded at the CERN LEP [151]. Signal cross sections are calculated at approximately NNLO+NNLL order in $\alpha _S$ [136–147], assuming decay branching fractions (${\mathcal {B}}$) as indicated in the figure

**Fig. 18:**
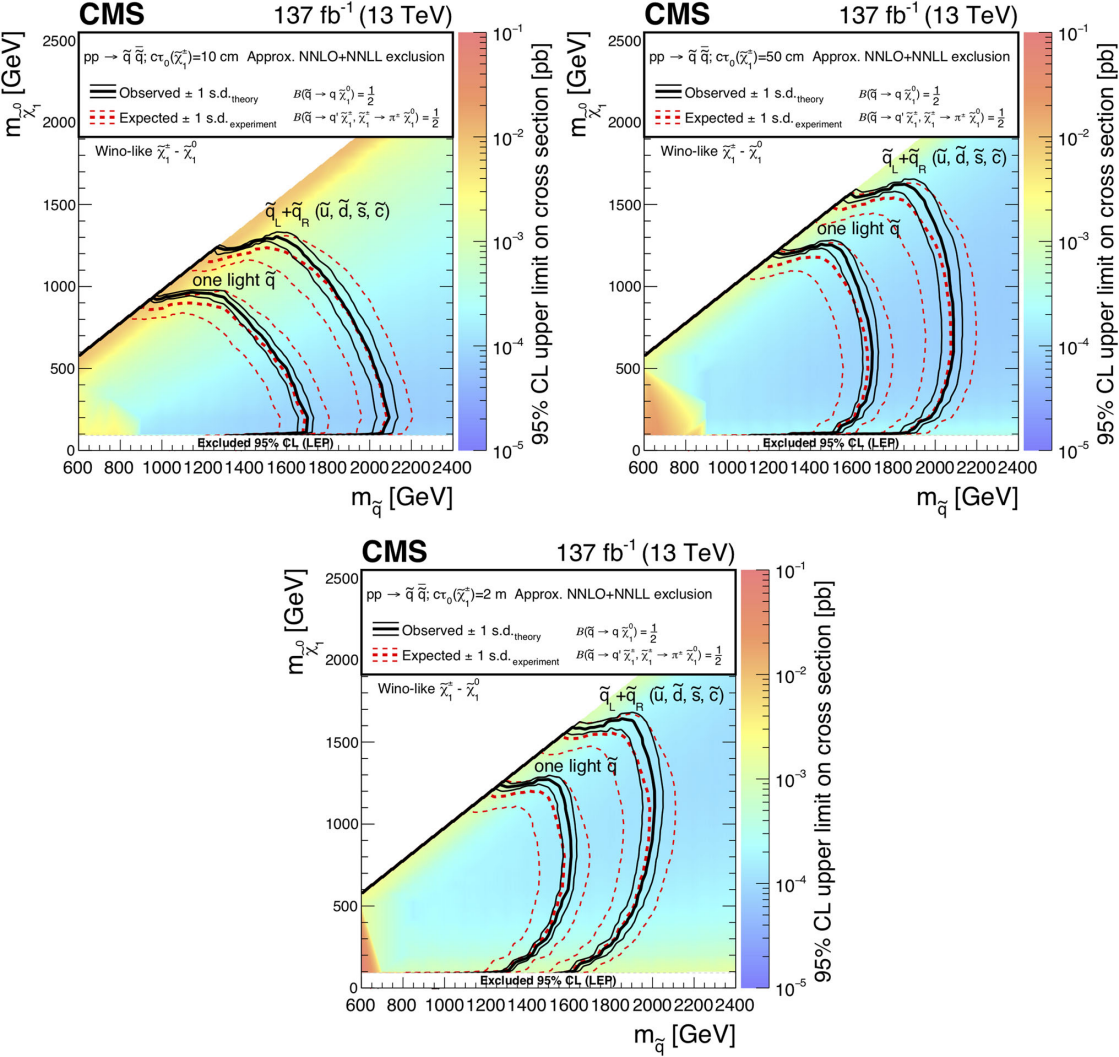
Exclusion limits at 95% $\text {CL}$ for light squark pair production with $c\tau _{0}({\tilde{{\upchi }}_{1}^{\pm }}) =$ (upper left) 10$\,\text {cm}$, (upper right) 50$\,\text {cm}$, and (lower) 200$\,\text {cm}$. The area enclosed by the thick black curve represents the observed exclusion region, while the dashed red lines indicate the expected limits and their ±1 standard deviation (s.d.) ranges. The thin black lines show the effect of the theoretical uncertainties in the signal cross section. The white band for masses of the $\tilde{{\upchi }}_{1}^{0}$ below 91.9$\,\,\text {Ge}\text {V}$ represents the region of the mass plane excluded at the CERN LEP [151]. Signal cross sections are calculated at approximately NNLO+NNLL order in $\alpha _S$ [136–147], assuming decay branching fractions (${\mathcal {B}}$) as indicated in the figure

**Fig. 19:**
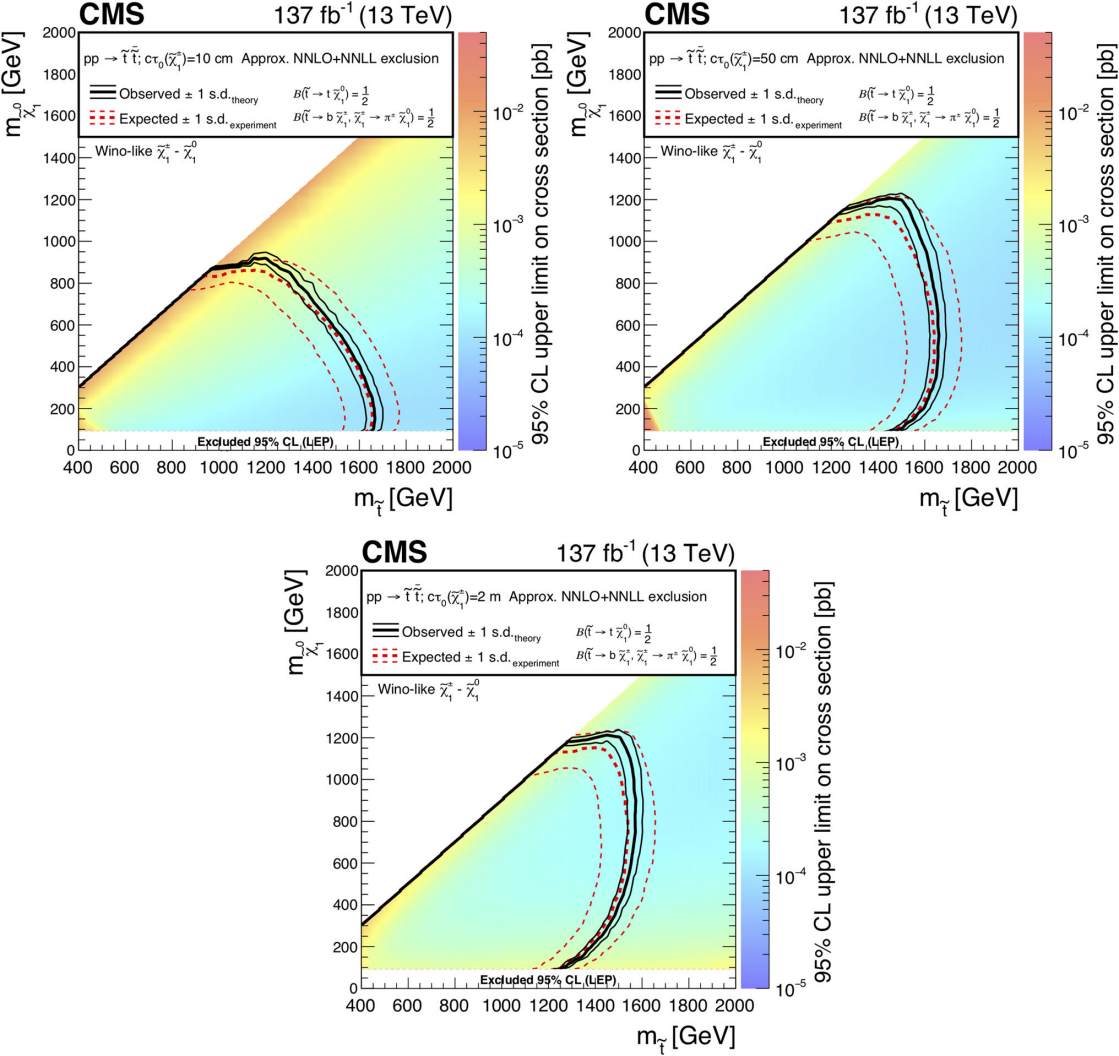
Exclusion limits at 95% $\text {CL}$ for top squark pair production with $c\tau _{0}({\tilde{{\upchi }}_{1}^{\pm }}) =$ (upper left) 10$\,\text {cm}$, (upper right) 50$\,\text {cm}$, and (lower) 200$\,\text {cm}$. The area enclosed by the thick black curve represents the observed exclusion region, while the dashed red lines indicate the expected limits and their ±1 standard deviation (s.d.) ranges. The thin black lines show the effect of the theoretical uncertainties in the signal cross section. The white band for masses of the $\tilde{{\upchi }}_{1}^{0}$ below 91.9$\,\,\text {Ge}\text {V}$ represents the region of the mass plane excluded at the CERN LEP [151]. Signal cross sections are calculated at approximately NNLO+NNLL order in $\alpha _S$ [136–147], assuming decay branching fractions (${\mathcal {B}}$) as indicated in the figure

Figure 17 shows the exclusion limits at 95% $\text {CL}$ for direct gluino pair production where the gluinos decay to light-flavor (u, d, s, c) quarks, with $c\tau _{0}({\tilde{{\upchi }}_{1}^{\pm }}) = 10$, 50, and 200$\,\text {cm}$. Exclusion limits for the direct production of light-flavor and top squark pairs are shown in Figs. 18 and 19, respectively, also for $c\tau _{0}({\tilde{{\upchi }}_{1}^{\pm }}) = 10$, 50, and 200$\,\text {cm}$.

Exclusion limits from the disappearing track search tend to be strongest in longer $c\tau _{0}({\tilde{{\upchi }}_{1}^{\pm }})$ models, when $m_{\tilde{{\upchi }}_{1}^{0}}$ is near the mass of the gluino or squark, and in shorter $c\tau _{0}({\tilde{{\upchi }}_{1}^{\pm }})$ models, when a large mass splitting generates a large boost for the ${\tilde{{\upchi }}_{1}^{\pm }}$, and in models characterized by large jet multiplicities. Models with these properties tend to populate the background depleted disappearing track regions with high $N_{\mathrm {j}}$ and longer tracks. In the massless ${\tilde{{\upchi }}_{1}^{\pm }}$ and $\tilde{{\upchi }}_{1}^{0}$ limit, the ${\tilde{{\upchi }}_{1}^{\pm }}$ receives a large Lorentz boost. Therefore, it tends not to decay inside the tracking detector, with a consequent reduction in the signal acceptance and in the analysis sensitivity.

When a ${\tilde{{\upchi }}_{1}^{\pm }}$ decays within the volume of the tracking detector, it is not counted as a PF candidate and, being almost mass degenerate with the $\tilde{{\upchi }}_{1}^{0}$, its decay products provide negligible visible energy in the detector. To a good approximation, as confirmed in simulation, the limits presented in Sect. 6.1 from the inclusive $M_{\mathrm {T2}}$ search should apply also to these models with an intermediate ${\tilde{{\upchi }}_{1}^{\pm }}$.

For SUSY models with long-lived ${\tilde{{\upchi }}_{1}^{\pm }}$, the search for disappearing tracks significantly extends the sensitivity of the inclusive $M_{\mathrm {T2}}$ search. Table 10 summarizes the limits on the masses of the SUSY particles excluded for the simplified model scenarios considered.

**Table 10:** Summary of the observed 95% $\text {CL}$ exclusion limits on the masses of SUSY particles for different simplified model scenarios, where the produced particles decay with equal probability to $\tilde{{\upchi }}_{1}^{+}$, $\tilde{{\upchi }}_{1}^{-}$, and $\tilde{{\upchi }}_{1}^{0}$, and the ${\tilde{{\upchi }}_{1}^{\pm }}$ are long lived. The highest limits on the mass of the directly produced particles and on the mass of the $\tilde{{\upchi }}_{1}^{0}$ are quoted

Simplified	Highest limit on directly produced	Highest limit on
model	SUSY particle mass ($\text {Ge}\text {V}$)	$\tilde{{\upchi }}_{1}^{0}$ mass ($\text {Ge}\text {V}$)
Direct gluino pair production:		
${\tilde{{\text {g}}}} \rightarrow {\text {q}} {\bar{{{\text {q}}}}} \tilde{{\upchi }}_{1}^{0}$ or ${\tilde{{\text {g}}}} \rightarrow {\text {q}} {\bar{{{\text {q}}}}} ' {\tilde{{\upchi }}_{1}^{\pm }}$	2460	2000
Direct squark pair production:		
Eight degenerate light squarks	2090	1650
Single light squark	1700	1275
Top squark	1660	1210

**Fig. 20:**
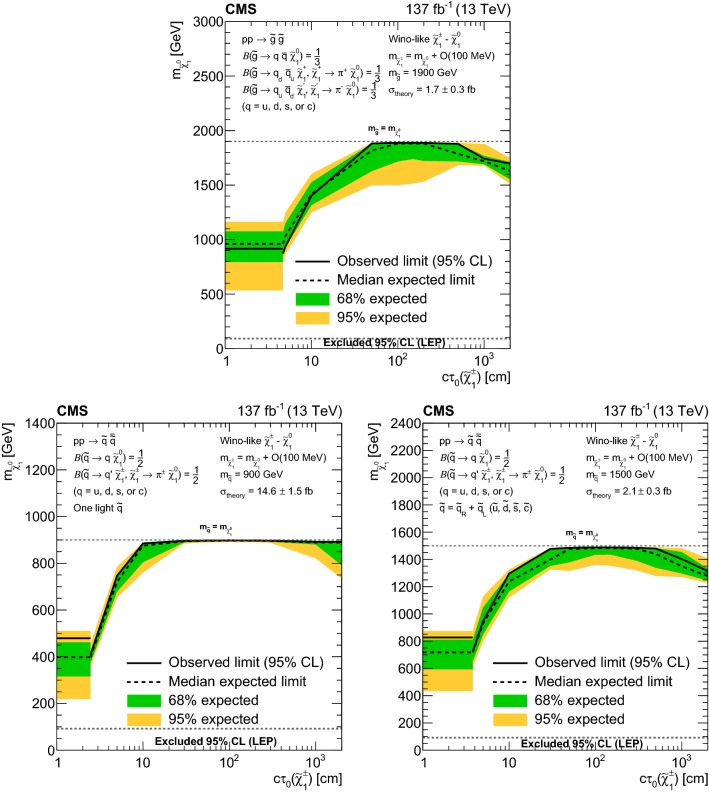
Exclusion limits at 95% $\text {CL}$ on the $\tilde{{\upchi }}_{1}^{0}$ mass, with $m_{{\tilde{{\upchi }}_{1}^{\pm }}}=m_{\tilde{{\upchi }}_{1}^{0}}+{\mathcal {O}}(100\,\,\text {Me}\text {V})$, as a function of the ${\tilde{{\upchi }}_{1}^{\pm }}$ proper decay length, for (upper) direct gluino and (lower) direct light-flavor (u, d, s, c) squark pair production, as obtained for representative gluino and squark masses. The gluinos decay to light-flavor quarks. For direct squark pair production, we assume either (lower left) one–fold or (lower right) eight–fold squark degeneracy. The area enclosed by the solid (dashed) black curve represents the observed (median expected) exclusion region, while the inner green (outer yellow) band indicates the region containing 68 (95)% of the distribution of limits expected under the background-only hypothesis. At short decay lengths, horizontal exclusion lines are obtained from the inclusive $M_{\mathrm {T2}}$ search, as this is not affected by track reconstruction inefficiencies, which may arise when the ${\tilde{{\upchi }}_{1}^{\pm }}$ decays before the CMS tracker, and therefore shows better sensitivity to scenarios with very small $c\tau _{0}({\tilde{{\upchi }}_{1}^{\pm }})$ compared to the disappearing track search, based on median expected limits. The horizontal dashed lines at (upper) $m_{{\tilde{{\text {g}}}}}=m_{\tilde{{\upchi }}_{1}^{0}}$ and (lower) $m_{\tilde{{\text {q}}}}=m_{\tilde{{\upchi }}_{1}^{0}}$ bound the mass range in which the decays are kinematically allowed. If all kinematically allowed $\tilde{{\upchi }}_{1}^{0}$ masses ($m_{\tilde{{\upchi }}_{1}^{0}} \le m_{{\tilde{{\text {g}}}}}$, or $m_{\tilde{{\upchi }}_{1}^{0}} \le m_{\tilde{{\text {q}}}}$) are excluded, the curves, including 68 and 95% expected, tend to overlap. The band at masses of the $\tilde{{\upchi }}_{1}^{0}$ below 91.9$\,\,\text {Ge}\text {V}$ represents the region of the mass plane excluded at the CERN LEP [151]. Signal cross sections are calculated at approximately NNLO+NNLL order in $\alpha _S$ [136–147], assuming decay branching fractions (${\mathcal {B}}$) as indicated in the figure

**Fig. 21:**
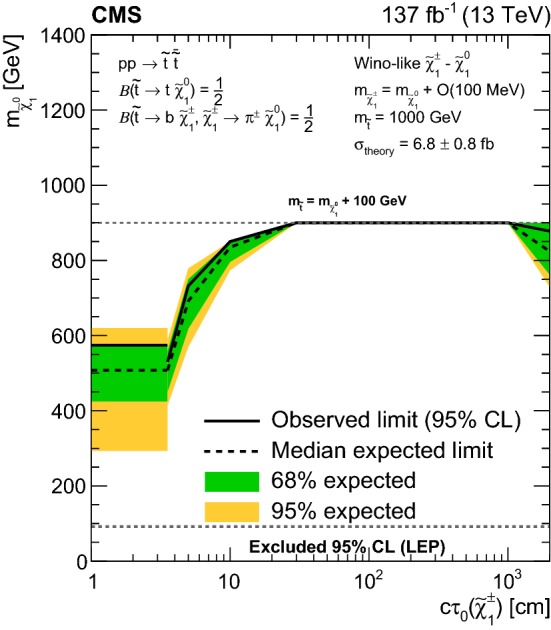
Exclusion limits at 95% $\text {CL}$ on the $\tilde{{\upchi }}_{1}^{0}$ mass, with $m_{{\tilde{{\upchi }}_{1}^{\pm }}}=m_{\tilde{{\upchi }}_{1}^{0}}+{\mathcal {O}}(100\,\,\text {Me}\text {V})$, as a function of the ${\tilde{{\upchi }}_{1}^{\pm }}$ proper decay length, for direct top squark pair production, as obtained for a representative top squark mass. The area enclosed by the solid (dashed) black curve represents the observed (median expected) exclusion region, while the inner green (outer yellow) band indicates the region containing 68 (95)% of the distribution of limits expected under the background-only hypothesis. At short decay lengths, horizontal exclusion lines are obtained from the inclusive $M_{\mathrm {T2}}$ search, as this is not affected by track reconstruction inefficiencies, which may arise when the ${\tilde{{\upchi }}_{1}^{\pm }}$ decays before the CMS tracker, and therefore shows better sensitivity to scenarios with very small $c\tau _{0}({\tilde{{\upchi }}_{1}^{\pm }})$ compared to the disappearing track search, based on median expected limits. The horizontal dashed line at $m_{\tilde{{\text {t}}}}=m_{\tilde{{\upchi }}_{1}^{0}}+100\,\,\text {Ge}\text {V}$ indicates the minimum simulated mass difference between top squark and $\tilde{{\upchi }}_{1}^{0}$, chosen such that the decay of top quarks to on-shell ${\text {W}}$ bosons is allowed. If all kinematically allowed $\tilde{{\upchi }}_{1}^{0}$ masses ($m_{\tilde{{\upchi }}_{1}^{0}} \le m_{\tilde{{\text {t}}}}-100\,\,\text {Ge}\text {V}$) are excluded, the curves, including 68 and 95% expected, tend to overlap. The band at masses of the $\tilde{{\upchi }}_{1}^{0}$ below 91.9$\,\,\text {Ge}\text {V}$ represents the region of the mass plane excluded at the CERN LEP [151]. Signal cross sections are calculated at approximately NNLO+NNLL order in $\alpha _S$ [136–147], assuming decay branching fractions (${\mathcal {B}}$) as indicated in the figure

Two-dimensional constraints are also placed on the ${\tilde{{\upchi }}_{1}^{\pm }}$ mass as a function of its proper decay length, as shown in Figs. 20 and 21, for the pair production of gluinos and light-flavor and top squarks, respectively. In particular, Figs. 20 and 21 show the excluded ${\tilde{{\upchi }}_{1}^{\pm }}$ mass as a function of its proper decay length for representative gluino, light-flavor or top squark masses. For short ${\tilde{{\upchi }}_{1}^{\pm }}$ lifetimes, the inclusive $M_{\mathrm {T2}}$ search is more sensitive than the dedicated search for disappearing tracks, based on expected exclusion limits. As already mentioned above, the inclusive $M_{\mathrm {T2}}$ search is not sensitive to the presence of an intermediate long-lived ${\tilde{{\upchi }}_{1}^{\pm }}$ in the parent SUSY particle decay chain, especially when the ${\tilde{{\upchi }}_{1}^{\pm }}$ lifetime is short, such that the ${\tilde{{\upchi }}_{1}^{\pm }}$ cannot be reconstructed as a stable PF candidate. Furthermore, the signal acceptance of the inclusive $M_{\mathrm {T2}}$ search is not affected by the track reconstruction inefficiencies which may arise when the ${\tilde{{\upchi }}_{1}^{\pm }}$ decays before the CMS tracker, for very short ${\tilde{{\upchi }}_{1}^{\pm }}$ lifetimes.

**Fig. 22:**
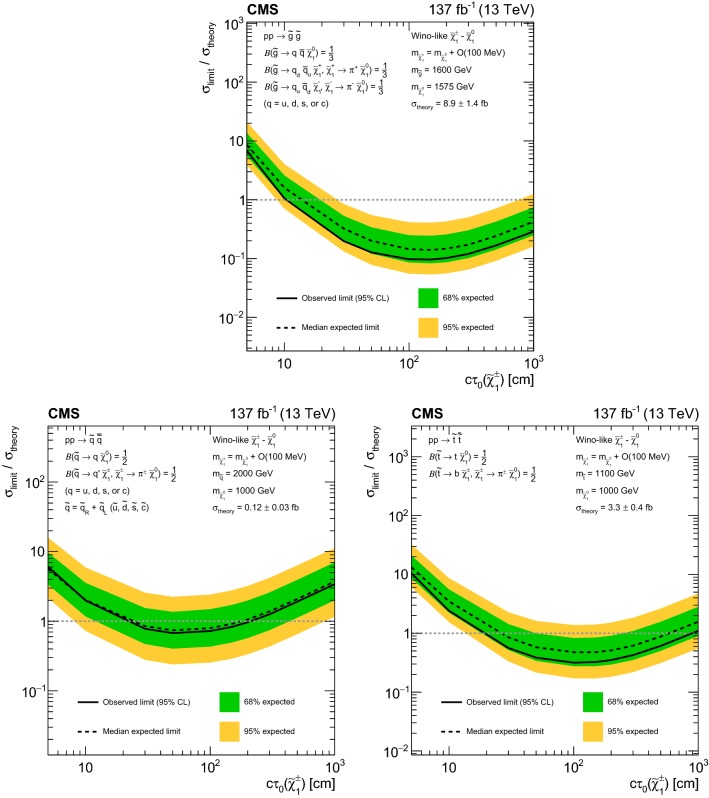
Exclusion limits at 95% $\text {CL}$ on $\sigma /\sigma _{\mathrm {theory}}$ as a function of the ${\tilde{{\upchi }}_{1}^{\pm }}$ decay length, for a choice of signal models of (upper) direct gluino pair production where the gluinos decay to light-flavor (u, d, s, c) quarks, (lower left) direct light-flavor squark pair production, and (lower right) direct top squark pair production, as obtained from the search for disappearing tracks. The area enclosed by the solid (dashed) black curve below the horizontal dashed line at $\sigma /\sigma _{\mathrm {theory}}=1$ represents the observed (median expected) exclusion region, while the inner green (outer yellow) band indicates the region containing 68 (95)% of the distribution of limits expected under the background-only hypothesis. Signal cross sections are calculated at approximately NNLO+NNLL order in $\alpha _S$ [136–147], assuming decay branching fractions (${\mathcal {B}}$) as indicated in the figure

**Fig. 23:**
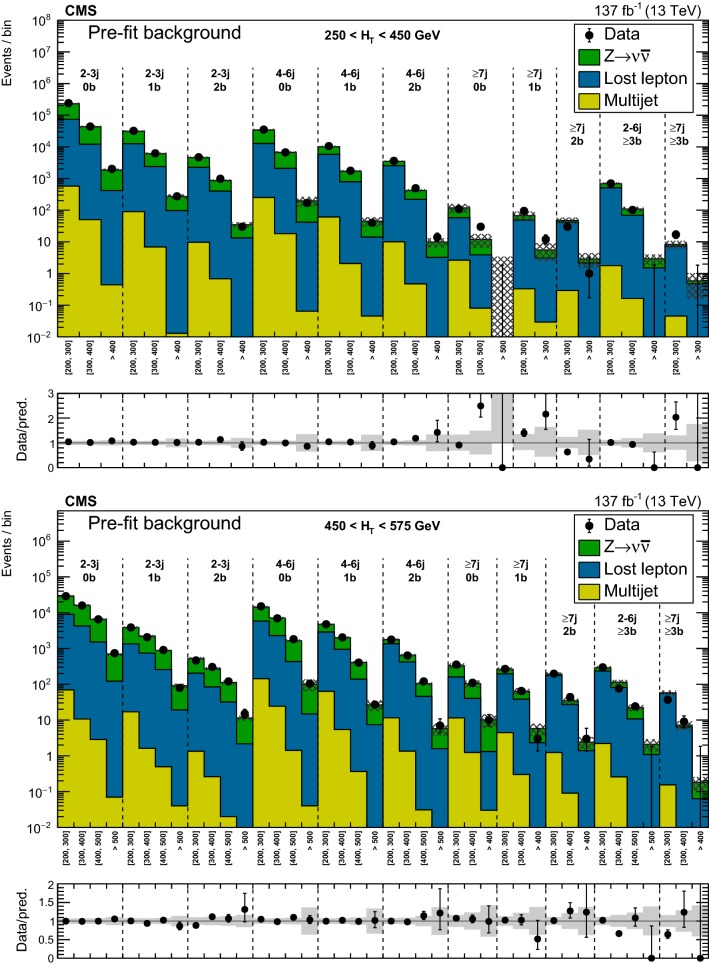
(Upper) Comparison of the estimated background and observed data events in each signal bin in the very-low-$H_{\mathrm {T}}$ region. The hatched bands represent the full uncertainty in the background estimate. The notations j, b indicate $N_{\mathrm {j}}$, $N_{{\text {b}}}$ labeling. (Lower) Same for the low-$H_{\mathrm {T}}$ region. On the *x* axis, the $M_{\mathrm {T2}}$ binning is shown in units of GeV

**Fig. 24:**
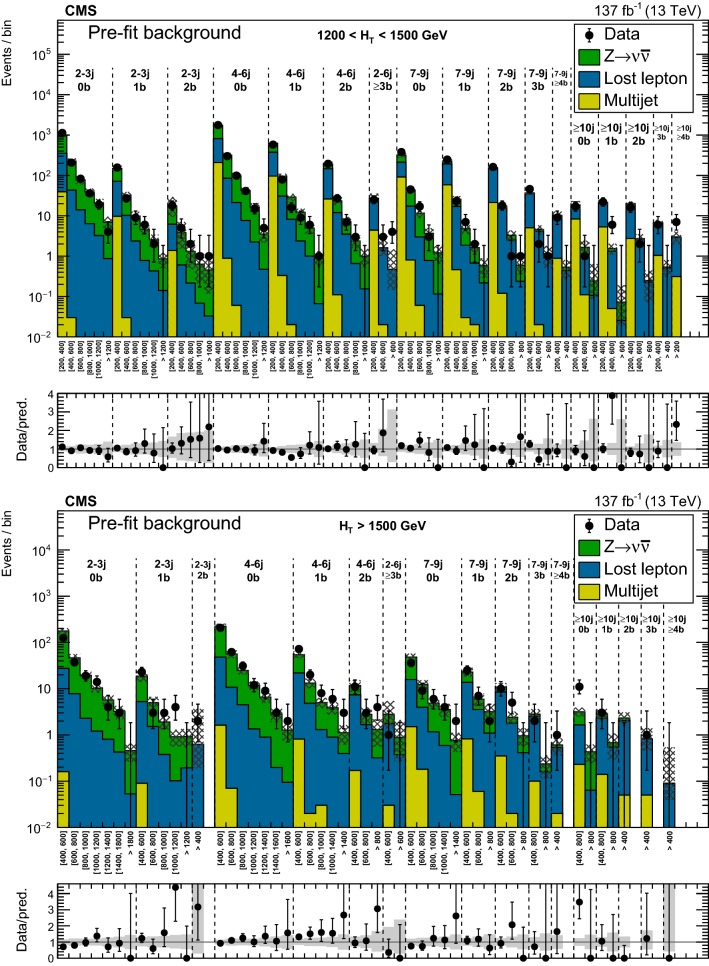
(Upper) Comparison of the estimated background and observed data events in each signal bin in the high-$H_{\mathrm {T}}$ region. The hatched bands represent the full uncertainty in the background estimate. The notations j, b indicate $N_{\mathrm {j}}$, $N_{{\text {b}}}$ labeling. (Lower) Same for the extreme-$H_{\mathrm {T}}$ region. On the *x* axis, the $M_{\mathrm {T2}}$ binning is shown in units of GeV

Figure 22 shows exclusion limits on $\sigma /\sigma _{\mathrm {theory}}$ as a function of $c\tau _{0}({\tilde{{\upchi }}_{1}^{\pm }})$, for a choice of signal models where gluinos and squarks can decay via a long-lived ${\tilde{{\upchi }}_{1}^{\pm }}$, as obtained from the search for disappearing tracks. Scenarios where the mass spectrum of SUSY particles is compressed are especially constrained across a wide range of $c\tau _{0}({\tilde{{\upchi }}_{1}^{\pm }})$. The exclusion limits are typically stronger at intermediate $c\tau _{0}({\tilde{{\upchi }}_{1}^{\pm }})$, as a larger fraction of ${\tilde{{\upchi }}_{1}^{\pm }}$ decay within the CMS tracker and can therefore be identified as disappearing tracks.

## Summary

This paper presents the results of two related searches for phenomena beyond the standard model using events with jets and large values of the kinematic variable $M_{\mathrm {T2}}$. The first is an inclusive search, while the second requires in addition disappearing tracks. The measurements are based on a data sample of proton–proton collisions at $\sqrt{s} =13\,\text {Te}\text {V}$ collected in 2016–2018 with the CMS detector, and corresponding to an integrated luminosity of 137$\,\text {fb}^{-1}$. No significant deviations from the standard model expectations are observed. Limits on pair-produced gluinos and squarks are established in the context of supersymmetry models conserving *R*-parity. The inclusive $M_{\mathrm {T2}}$ search probes gluino masses up to 2250$\,\,\text {Ge}\text {V}$ and the lightest neutralino $\tilde{{\upchi }}_{1}^{0}$ masses up to 1525$\,\,\text {Ge}\text {V}$, as well as light-flavor, bottom, and top squark masses up to 1710, 1240, and 1200$\,\,\text {Ge}\text {V}$, respectively, and $\tilde{{\upchi }}_{1}^{0}$ masses up to 870, 700, and 580$\,\,\text {Ge}\text {V}$ in each respective scenario. In models with a long-lived chargino ${\tilde{{\upchi }}_{1}^{\pm }}$, where the gluinos and squarks decay with equal probability to $\tilde{{\upchi }}_{1}^{0}$, $\tilde{{\upchi }}_{1}^{+}$, and $\tilde{{\upchi }}_{1}^{-}$, the search looking in addition for disappearing tracks probes gluino masses up to 2460$\,\,\text {Ge}\text {V}$ and $\tilde{{\upchi }}_{1}^{0}$ masses up to 2000$\,\,\text {Ge}\text {V}$, as well as light-flavor (top) squark masses up to 2090 (1660)$\,\,\text {Ge}\text {V}$ and $\tilde{{\upchi }}_{1}^{0}$ masses up to 1650 (1210)$\,\,\text {Ge}\text {V}$.

A resonantly produced colored scalar state $\phi$ decaying to a massive Dirac fermion $\psi$ and a quark has recently been proposed as an explanation of an excess in data identified in regions with low jet multiplicities, based on previous results by the ATLAS and CMS Collaborations. From the inclusive $M_{\mathrm {T2}}$ search, mass limits as high as 1660 and 925$\,\,\text {Ge}\text {V}$ are obtained for $\phi$ and $\psi$, respectively, and an upper limit on the product of the cross section and branching fraction of about 0.6$\,\,\text {pb}$ with a local significance of 1.1 standard deviations is observed for the previously reported best fit point $\left( m_{\phi },m_{\psi }\right) =\left( 1250,900\right) \,\,\text {Ge}\text {V}$. The inclusive $M_{\mathrm {T2}}$ search is also used to constrain models of scalar and vector leptoquark (LQ) pair production with the LQ decaying to a neutrino and a top, bottom, or light-flavor quark. A vector LQ decaying with equal branching fraction to ${\text {t}} {{\upnu }}$ and ${\text {b}} {\uptau }$ has been proposed as part of an explanation of recent flavor anomalies. In such a model, LQ masses below 1550$\,\,\text {Ge}\text {V}$ are excluded assuming the Yang–Mills case with coupling $\kappa =1$, or 1225$\,\,\text {Ge}\text {V}$ in the minimal coupling case $\kappa =0$. The results presented in this paper extend the mass limits of the previous version of the CMS inclusive $M_{\mathrm {T2}}$ search, using a subset of the present data, by hundreds of $\,\,\text {Ge}\text {V}$. In most of the cases, the results obtained are the most stringent constraints to date.

## Data Availability

This manuscript has no associated data or the data will not be deposited. [Authors’ comment: Release and preservation of data used by the CMS Collaboration as the basis for publications is guided by the CMS policy as written in its document “CMS data preservation, re-use and open access policy” (https://cms-docdb.cern.ch/cgi-bin/PublicDocDB/RetrieveFile?docid=6032&filename=CMSDataPolicyV1.2.pdf&version=2).]

## Disappearing track selection

The detailed selection of disappearing tracks (STs and STCs, as defined in Sect. 3.2.2) is summarized in Table 11.

**Table 11:** Selection requirements for STs and STCs. For the subset of medium (M) length tracks that have just four tracking layers with a measurement, the minimum required number of layers of the pixel tracking detector with a measurement is three ($\dagger$). The selected tracks are required to not overlap with identified leptons. For this selection, all electrons and muons are considered, either identified as PF candidates or not. The selected tracks are as well required to not be identified as PF candidates, and to not overlap with other tracks with $p_{\mathrm {T}} >15\,\,\text {Ge}\text {V}$, even if those tracks are not associated with PF candidates. The factor by which the selection requirement is relaxed in order to select short track candidates is also reported. If no factor is reported, the requirement is not relaxed for the selection of short track candidates

Observable	Selection	Track length	STC factor
$p_{\mathrm {T}}$ ($\text {Ge}\text {V}$)	>15	All	
$|\eta |$	$<2.4$ and not $1.38< |\eta | < 1.6$	All	
$\sigma (p_{\mathrm {T}})$ / $p_{\mathrm {T}} ^2$ ($\text {Ge}\text {V}$ $^{-1}$)	$<0.2$; $<0.02$; $<0.005$	P; M; L	3
$d_{\mathrm {xy}}$ (from primary vertex) [cm]	$<0.02$ ( $<0.01$ )	P ( M, L )	3
$d_{\mathrm {z}}$ (from primary vertex) [cm]	$<0.05$	All	3
Neutral isolation ($\varDelta R < 0.05$) ($\text {Ge}\text {V}$)	$<10$	All	6
Neutral isolation / $p_{\mathrm {T}}$	$<0.1$	All	6
Isolation ($\varDelta R < 0.3$) ($\text {Ge}\text {V}$)	$<10$	All	6
Isolation / $p_{\mathrm {T}}$	$<0.2$	All	6
Number of pixel layers	$\ge 3$ ( $\ge 2$ )	P, M$^{\dagger }$ ( M, L )	
Number of tracker layers	$\ge 3$; $<7$; $\ge 7$	P; M; L	
Number of lost inner hits	$=$0	All	
Number of lost outer hits	$\ge 2$	M, L	
Is a PF candidate?	No	All	
PF lepton veto ($\varDelta R < 0.1$)	Yes	All	
Lepton veto ($\varDelta R < 0.2$)	Yes	All	
Track veto ($\varDelta R < 0.1$)	Yes	All	
Bad calorimeter module veto	Yes	All	
$M_{\mathrm {T}}$ (track, ${\vec p}_{\mathrm {T}}^{\text {miss}}$) ($\text {Ge}\text {V}$)	>100, if $p_{\mathrm {T}} < 150\,\,\text {Ge}\text {V}$	L	

## Definition of search regions and yields

### Inclusive $M_{\mathrm {T2}}$ search: search regions and yields

The 282 exclusive search regions defined for the inclusive $M_{\mathrm {T2}}$ search, as described in Sect. 3.2.1, are summarized in Tables 12, 13, 14, 15, 16, 17, 18, 19, 20, 21, 22 and 23, together with the pre-fit background predictions and the observed yields.

**Table 12:** Predictions and observations for the 12 search regions with $N_{\mathrm {j}} = 1$. For each of the background predictions, the first uncertainty listed is statistical (from the limited size of data control samples and Monte Carlo samples), and the second is systematic

$N_{\mathrm {j}}$, $N_{{\text {b}}}$	$p_{\mathrm {T}} ^{\text {jet}}$ ($\text {Ge}\text {V}$)	Lost lepton	${\text {Z}} \rightarrow {{\upnu }} \bar{{{\upnu }}}$	Multijet	Total background	Data
1j, 0b	250–350	$70\,700\pm 400\pm 4100$	$167\,000\pm 1000\pm 11\,000$	$530\pm 20\pm 160$	$238\,000\pm 1000\pm 14\,000$	251 941
	350–450	$13\,440\pm 130\pm 790$	$40\,100\pm 500\pm 3100$	$55\pm 5\pm 16$	$53\,600\pm 500\pm 3700$	54 870
	450–575	$3050\pm 50\pm 180$	$10\,850^{+230}_{-220}\pm 690$	$5.6\pm 1.1\pm 1.6$	$13\,910\pm 230\pm 840$	14 473
	575–700	$603^{+20}_{-19}\pm 38$	$2590^{+110}_{-100}\pm 160$	$0.38\pm 0.06\pm 0.11$	$3200\pm 110\pm 190$	3432
	700–1000	$220\pm 13\pm 16$	$1076^{+70}_{-66}\pm 66$	$0.12\pm 0.03\pm 0.03$	$1295^{+71}_{-67}\pm 79$	1304
	1000–1200	$11.7^{+4.1}_{-3.2}\pm 0.9$	$86^{+23}_{-19}\pm 6$	$<0.01$	$98^{+24}_{-19}\pm 7$	98
	$\ge 1200$	$2.8^{+2.7}_{-1.5}\pm 0.6$	$23^{+12}_{-8}\pm 2$	$<0.01$	$26^{+13}_{-9}\pm 2$	30
1j, $\ge 1$b	250–350	$4210\pm 110\pm 260$	$9030\pm 230\pm 630$	$58\pm 10\pm 17$	$13\,310^{+260}_{-250}\pm 820$	13 549
	350–450	$878\pm 38\pm 56$	$2180^{+110}_{-100}\pm 170$	$4.6\pm 0.4\pm 1.3$	$3060\pm 110\pm 220$	3078
	450–575	$211^{+16}_{-15}\pm 13$	$651^{+57}_{-53}\pm 44$	$0.63\pm 0.18\pm 0.18$	$863^{+59}_{-55}\pm 53$	810
	575–700	$40.3^{+6.0}_{-5.5}\pm 2.5$	$164^{+30}_{-26}\pm 11$	$0.04\pm 0.02\pm 0.02$	$205^{+31}_{-26}\pm 13$	184
	$\ge 700$	$19.2^{+5.7}_{-4.6}\pm 1.3$	$74^{+21}_{-16}\pm 7$	$<0.01$	$94^{+21}_{-17}\pm 7$	83

**Table 13:** Predictions and observations for the 30 search regions with $250 \le H_{\mathrm {T}} < 450\,\,\text {Ge}\text {V}$. For each of the background predictions, the first uncertainty listed is statistical (from the limited size of data control samples and Monte Carlo samples), and the second is systematic

$N_{\mathrm {j}}$, $N_{{\text {b}}}$	$M_{\mathrm {T2}}$ ($\text {Ge}\text {V}$)	Lost lepton	${\text {Z}} \rightarrow {{\upnu }} \bar{{{\upnu }}}$	Multijet	Total background	Data
$250 \le H_{\mathrm {T}} < 450\,\,\text {Ge}\text {V}$:						
2-3j, 0b	200–300	$73\,700\pm 500\pm 5000$	$156\,000\pm 1000\pm 12\,000$	$580\pm 20\pm 140$	$231\,000\pm 1000\pm 16\,000$	240 867
	300–400	$12\,030\pm 200\pm 820$	$31\,300\pm 200\pm 2500$	$50\pm 5\pm 10$	$43\,400\pm 300\pm 3200$	44 074
	$\ge 400$	$417^{+51}_{-47}\pm 28$	$1450\pm 10\pm 140$	$0.44\pm 0.09\pm 0.09$	$1870\pm 50\pm 160$	2022
2-3j, 1b	200–300	$12\,450\pm 170\pm 820$	$18\,700\pm 300\pm 1500$	$90\pm 8\pm 21$	$31\,300\pm 300\pm 2200$	32 120
	300–400	$2380\pm 80\pm 160$	$3750\pm 60\pm 310$	$6.9\pm 1.0\pm 1.5$	$6130\pm 100\pm 430$	6258
	$\ge 400$	$97\pm 8\pm 39$	$174\pm 3\pm 17$	$0.01\pm 0.01\pm 0.00$	$271^{+9}_{-8}\pm 45$	275
2-3j, 2b	200–300	$2240\pm 70\pm 150$	$2340^{+110}_{-100}\pm 200$	$9.7\pm 1.1\pm 2.3$	$4600^{+130}_{-120}\pm 320$	4709
	300–400	$398^{+34}_{-32}\pm 27$	$469^{+21}_{-20}\pm 39$	$0.68\pm 0.17\pm 0.15$	$868^{+40}_{-38}\pm 61$	984
	$\ge 400$	$13.3\pm 2.3\pm 5.4$	$21.7^{+1.0}_{-0.9}\pm 2.2$	$<0.01$	$35.0\pm 2.5\pm 6.0$	30
2-6j, $\ge 3$b	200–300	$507^{+32}_{-31}\pm 38$	$179^{+35}_{-30}\pm 27$	$1.77\pm 0.46\pm 0.46$	$688^{+47}_{-43}\pm 54$	699
	300–400	$69\pm 6\pm 15$	$40.0^{+7.8}_{-6.6}\pm 6.0$	$0.16\pm 0.12\pm 0.04$	$109^{+10}_{-9}\pm 16$	102
	$\ge 400$	$1.50\pm 0.80\pm 0.61$	$1.43^{+0.28}_{-0.24}\pm 0.25$	$<0.01$	$2.92^{+0.85}_{-0.83}\pm 0.67$	0
4-6j, 0b	200–300	$12\,500\pm 180\pm 800$	$21\,600\pm 300\pm 1800$	$250\pm 17\pm 58$	$34\,400\pm 400\pm 2400$	35 187
	300–400	$2070\pm 80\pm 130$	$4660\pm 70\pm 410$	$18.2\pm 3.6\pm 3.8$	$6750\pm 110\pm 510$	6725
	$\ge 400$	$42\pm 5\pm 17$	$155\pm 2\pm 64$	$0.06\pm 0.03\pm 0.01$	$197\pm 5\pm 67$	170
4-6j, 1b	200–300	$5750\pm 100\pm 380$	$4300\pm 150\pm 360$	$61\pm 7\pm 15$	$10\,120\pm 180\pm 680$	10 564
	300–400	$784^{+43}_{-42}\pm 52$	$928^{+32}_{-31}\pm 84$	$2.07\pm 0.29\pm 0.45$	$1710\pm 50\pm 120$	1769
	$\ge 400$	$14.0\pm 2.5\pm 5.7$	$31\pm 1\pm 13$	$0.04\pm 0.02\pm 0.01$	$45\pm 3\pm 14$	40
4-6j, 2b	200–300	$2550^{+70}_{-60}\pm 170$	$921^{+68}_{-63}\pm 87$	$10.0\pm 1.5\pm 2.2$	$3480\pm 90\pm 230$	3621
	300–400	$220^{+23}_{-21}\pm 15$	$198^{+15}_{-14}\pm 20$	$0.47\pm 0.15\pm 0.11$	$419^{+27}_{-25}\pm 31$	496
	$\ge 400$	$3.2\pm 0.8\pm 1.3$	$6.6\pm 0.5\pm 2.7$	$<0.01$	$9.8\pm 0.9\pm 3.1$	14
$\ge 7$j, 0b	200–300	$55^{+15}_{-13}\pm 4$	$61^{+23}_{-17}\pm 26$	$2.64\pm 0.39\pm 0.57$	$119^{+28}_{-22}\pm 27$	108
	300–500	$3.8^{+2.1}_{-2.0}\pm 0.8$	$8.1^{+3.1}_{-2.3}\pm 4.3$	$0.08\pm 0.04\pm 0.02$	$12.0^{+3.7}_{-3.1}\pm 4.4$	30
	$\ge 500$	$0.0^{+3.2}_{-0.0}\pm 0.0$	$0.0^{+1.2}_{-0.0}\pm 0.0$	$<0.01$	$0.0^{+3.4}_{-0.0}\pm 0.0$	0
$\ge 7$j, 1b	200–300	$48.0^{+9.1}_{-8.2}\pm 3.5$	$19^{+19}_{-11}\pm 10$	$0.33\pm 0.14\pm 0.09$	$68^{+21}_{-13}\pm 11$	95
	$\ge 300$	$3.0\pm 1.4\pm 1.2$	$2.5^{+2.4}_{-1.3}\pm 1.7$	$0.03\pm 0.02\pm 0.01$	$5.6^{+2.8}_{-1.9}\pm 2.1$	12
$\ge 7$j, 2b	200–300	$41.3^{+7.7}_{-7.0}\pm 3.1$	$6.0^{+5.8}_{-3.2}\pm 3.7$	$0.29\pm 0.14\pm 0.06$	$47.6^{+9.7}_{-7.7}\pm 5.0$	30
	$\ge 300$	$2.15^{+0.78}_{-0.76}\pm 0.87$	$0.74^{+0.72}_{-0.40}\pm 0.57$	$<0.01$	$2.9^{+1.1}_{-0.9}\pm 1.1$	1
$\ge 7$j, $\ge 3$b	200–300	$7.3^{+1.7}_{-1.5}\pm 0.9$	$1.0^{+1.0}_{-0.6}\pm 1.1$	$0.04\pm 0.04\pm 0.01$	$8.4^{+1.9}_{-1.6}\pm 1.5$	17
	$\ge 300$	$0.47\pm 0.35\pm 0.20$	$0.12^{+0.11}_{-0.06}\pm 0.14$	$<0.01$	$0.59^{+0.37}_{-0.35}\pm 0.24$	0

**Table 14:** Predictions and observations for the 28 search regions with $450 \le H_{\mathrm {T}} < 575\,\,\text {Ge}\text {V}$, and $2 \le N_{\mathrm {j}} \le 3$, $2 \le N_{\mathrm {j}} \le 6$ and $N_{{\text {b}}} \ge 3$, or $4 \le N_{\mathrm {j}} \le 6$. For each of the background predictions, the first uncertainty listed is statistical (from the limited size of data control samples and Monte Carlo samples), and the second is systematic

$N_{\mathrm {j}}$, $N_{{\text {b}}}$	$M_{\mathrm {T2}}$ ($\text {Ge}\text {V}$)	Lost lepton	${\text {Z}} \rightarrow {{\upnu }} \bar{{{\upnu }}}$	Multijet	Total background	Data
$450 \le H_{\mathrm {T}} < 575\,\,\text {Ge}\text {V}$:						
2-3j, 0b	200–300	$8860\pm 110\pm 640$	$20\,100\pm 200\pm 1300$	$69\pm 13\pm 16$	$29\,100\pm 300\pm 1900$	28 956
	300–400	$4230\pm 80\pm 300$	$11\,770\pm 140\pm 790$	$10.6\pm 0.8\pm 2.4$	$16\,000\pm 200\pm 1000$	15 876
	400–500	$1510\pm 60\pm 110$	$5020\pm 60\pm 360$	$2.86\pm 0.62\pm 0.60$	$6540\pm 80\pm 440$	6527
	$\ge 500$	$121^{+24}_{-21}\pm 9$	$580\pm 7\pm 63$	$0.07\pm 0.03\pm 0.02$	$701^{+25}_{-22}\pm 68$	740
2-3j, 1b	200–300	$1326\pm 43\pm 88$	$2500\pm 80\pm 170$	$17.0\pm 8.4\pm 3.8$	$3840^{+100}_{-90}\pm 240$	3859
	300–400	$737\pm 35\pm 49$	$1464^{+49}_{-48}\pm 99$	$1.62\pm 0.20\pm 0.43$	$2200\pm 60\pm 140$	2065
	400–500	$259^{+25}_{-23}\pm 19$	$626^{+21}_{-20}\pm 45$	$0.49\pm 0.10\pm 0.12$	$885^{+32}_{-31}\pm 58$	907
	$\ge 500$	$19.1^{+2.8}_{-2.7}\pm 7.8$	$72.4\pm 2.4\pm 7.9$	$0.04\pm 0.02\pm 0.02$	$92\pm 4\pm 11$	79
2-3j, 2b	200–300	$201\pm 15\pm 13$	$322^{+31}_{-28}\pm 25$	$1.34\pm 0.62\pm 0.47$	$524^{+35}_{-32}\pm 35$	463
	300–400	$83.8^{+9.6}_{-9.1}\pm 9.1$	$188^{+18}_{-17}\pm 15$	$0.26\pm 0.07\pm 0.07$	$272^{+21}_{-19}\pm 20$	304
	400–500	$31.8^{+4.1}_{-4.0}\pm 6.7$	$80.4^{+7.7}_{-7.1}\pm 6.6$	$0.02\pm 0.01\pm 0.01$	$112^{+9}_{-8}\pm 10$	120
	$\ge 500$	$2.16^{+0.67}_{-0.66}\pm 0.88$	$9.3^{+0.9}_{-0.8}\pm 1.1$	$<0.01$	$11.4\pm 1.1\pm 1.4$	15
2-6j, $\ge 3$b	200–300	$232^{+17}_{-16}\pm 15$	$57^{+17}_{-13}\pm 7$	$2.20\pm 0.70\pm 0.80$	$291^{+24}_{-21}\pm 19$	297
	300–400	$81^{+12}_{-11}\pm 6$	$33.6^{+9.9}_{-7.8}\pm 4.3$	$0.26\pm 0.08\pm 0.08$	$115^{+16}_{-14}\pm 8$	76
	400–500	$10.7^{+2.1}_{-2.0}\pm 2.3$	$11.4^{+3.4}_{-2.7}\pm 1.5$	$<0.01$	$22.1^{+4.0}_{-3.4}\pm 2.8$	24
	$\ge 500$	$1.08\pm 0.58\pm 0.44$	$1.03^{+0.30}_{-0.24}\pm 0.17$	$<0.01$	$2.11^{+0.65}_{-0.62}\pm 0.48$	0
4-6j, 0b	200–300	$5660\pm 90\pm 370$	$8560\pm 170\pm 600$	$143\pm 7\pm 35$	$14\,360\pm 190\pm 890$	15 047
	300–400	$2250\pm 60\pm 150$	$4790^{+100}_{-90}\pm 350$	$24.3\pm 2.6\pm 6.2$	$7060\pm 110\pm 460$	6939
	400–500	$428^{+32}_{-30}\pm 28$	$1220\pm 20\pm 110$	$1.42\pm 0.21\pm 0.52$	$1650\pm 40\pm 130$	1817
	$\ge 500$	$14.8\pm 2.2\pm 6.0$	$86\pm 2\pm 35$	$0.04\pm 0.02\pm 0.01$	$101\pm 3\pm 36$	104
4-6j, 1b	200–300	$2810\pm 60\pm 190$	$1880\pm 80\pm 130$	$63\pm 15\pm 19$	$4750\pm 100\pm 300$	4736
	300–400	$937\pm 36\pm 63$	$1054^{+45}_{-43}\pm 78$	$5.4\pm 0.4\pm 1.4$	$2000\pm 60\pm 130$	2039
	400–500	$138^{+17}_{-16}\pm 10$	$269\pm 11\pm 25$	$0.36\pm 0.10\pm 0.10$	$407^{+20}_{-19}\pm 31$	403
	$\ge 500$	$7.5\pm 2.2\pm 3.0$	$19.1\pm 0.8\pm 7.9$	$0.01\pm 0.01\pm 0.00$	$26.5\pm 2.3\pm 8.5$	27
4-6j, 2b	200–300	$1343^{+38}_{-37}\pm 89$	$414^{+39}_{-35}\pm 33$	$11.5\pm 1.0\pm 3.3$	$1770\pm 50\pm 110$	1767
	300–400	$418^{+24}_{-23}\pm 29$	$232^{+22}_{-20}\pm 19$	$1.35\pm 0.35\pm 0.39$	$651^{+32}_{-31}\pm 43$	636
	400–500	$45.6^{+3.9}_{-3.8}\pm 9.6$	$59.1^{+5.5}_{-5.1}\pm 5.9$	$0.03\pm 0.02\pm 0.01$	$105^{+7}_{-6}\pm 12$	120
	$\ge 500$	$1.59\pm 0.89\pm 0.65$	$4.2\pm 0.4\pm 1.7$	$<0.01$	$5.8\pm 1.0\pm 1.9$	7

**Table 15:** Predictions and observations for the 12 search regions with $450 \le H_{\mathrm {T}} < 575\,\,\text {Ge}\text {V}$ and $N_{\mathrm {j}} \ge 7$. For each of the background predictions, the first uncertainty listed is statistical (from the limited size of data control samples and Monte Carlo samples), and the second is systematic

$N_{\mathrm {j}}$, $N_{{\text {b}}}$	$M_{\mathrm {T2}}$ ($\text {Ge}\text {V}$)	Lost lepton	${\text {Z}} \rightarrow {{\upnu }} \bar{{{\upnu }}}$	Multijet	Total background	Data
$450 \le H_{\mathrm {T}} < 575\,\,\text {Ge}\text {V}$:						
$\ge 7$j, 0b	200–300	$149^{+17}_{-16}\pm 13$	$169^{+31}_{-27}\pm 34$	$11.5\pm 0.8\pm 3.0$	$329^{+36}_{-31}\pm 38$	354
	300–400	$38.9^{+5.8}_{-5.6}\pm 8.2$	$64^{+12}_{-10}\pm 17$	$1.24\pm 0.42\pm 0.32$	$104^{+13}_{-12}\pm 20$	110
	$\ge 400$	$1.28\pm 0.82\pm 0.52$	$8.8^{+1.6}_{-1.4}\pm 3.8$	$0.03\pm 0.02\pm 0.01$	$10.1^{+1.8}_{-1.6}\pm 3.8$	10
$\ge 7$j, 1b	200–300	$191^{+13}_{-12}\pm 15$	$67^{+19}_{-15}\pm 15$	$4.4\pm 0.5\pm 1.2$	$262^{+23}_{-19}\pm 23$	268
	300–400	$37.8^{+3.4}_{-3.3}\pm 8.0$	$25.3^{+7.2}_{-5.7}\pm 7.3$	$0.30\pm 0.07\pm 0.08$	$63^{+8}_{-7}\pm 11$	65
	$\ge 400$	$2.31\pm 0.69\pm 0.94$	$3.5^{+1.0}_{-0.8}\pm 1.5$	$0.01\pm 0.01\pm 0.00$	$5.8^{+1.2}_{-1.0}\pm 1.8$	3
$\ge 7$j, 2b	200–300	$173^{+12}_{-11}\pm 13$	$19.9^{+5.7}_{-4.5}\pm 5.2$	$1.24\pm 0.18\pm 0.33$	$194^{+13}_{-12}\pm 15$	197
	300–400	$26.8\pm 2.6\pm 5.7$	$7.6^{+2.2}_{-1.7}\pm 2.4$	$0.09\pm 0.04\pm 0.03$	$34.6^{+3.4}_{-3.1}\pm 6.3$	44
	$\ge 400$	$1.40\pm 0.44\pm 0.57$	$1.02^{+0.29}_{-0.23}\pm 0.46$	$<0.01$	$2.42^{+0.53}_{-0.49}\pm 0.73$	3
$\ge 7$j, $\ge 3$b	200–300	$55.4^{+4.8}_{-4.7}\pm 7.3$	$2.3^{+0.7}_{-0.5}\pm 1.1$	$0.15\pm 0.06\pm 0.06$	$57.8^{+4.8}_{-4.7}\pm 7.4$	37
	300–400	$6.4\pm 1.2\pm 1.5$	$0.86^{+0.25}_{-0.20}\pm 0.46$	$0.01\pm 0.01\pm 0.00$	$7.3\pm 1.2\pm 1.6$	9
	$\ge 400$	$0.06\pm 0.01\pm 0.03$	$0.12\pm 0.03\pm 0.06$	$<0.01$	$0.18^{+0.04}_{-0.03}\pm 0.07$	0

**Table 16:** Predictions and observations for the 21 search regions with $575 \le H_{\mathrm {T}} < 1200\,\,\text {Ge}\text {V}$ and $2 \le N_{\mathrm {j}} \le 3$. For each of the background predictions, the first uncertainty listed is statistical (from the limited size of data control samples and Monte Carlo samples), and the second is systematic

$N_{\mathrm {j}}$, $N_{{\text {b}}}$	$M_{\mathrm {T2}}$ ($\text {Ge}\text {V}$)	Lost lepton	${\text {Z}} \rightarrow {{\upnu }} \bar{{{\upnu }}}$	Multijet	Total background	Data
$575 \le H_{\mathrm {T}} < 1200\,\,\text {Ge}\text {V}$:						
2-3j, 0b	200–300	$5270\pm 60\pm 370$	$11\,550\pm 160\pm 790$	$93\pm 20\pm 30$	$16\,900\pm 200\pm 1100$	17 256
	300–400	$2560\pm 50\pm 180$	$7770^{+110}_{-100}\pm 540$	$11.9\pm 1.3\pm 4.4$	$10\,340^{+120}_{-110}\pm 680$	10 145
	400–500	$1101^{+32}_{-31}\pm 77$	$3900\pm 50\pm 280$	$1.33\pm 0.24\pm 0.41$	$5000\pm 60\pm 340$	5021
	500–600	$502^{+24}_{-23}\pm 35$	$2250\pm 30\pm 170$	$0.37\pm 0.07\pm 0.12$	$2760\pm 40\pm 200$	2706
	600–700	$180^{+16}_{-15}\pm 13$	$746\pm 10\pm 73$	$0.09\pm 0.03\pm 0.03$	$926^{+19}_{-18}\pm 80$	1066
	700–800	$52.1^{+7.3}_{-6.5}\pm 5.5$	$256\pm 3\pm 36$	$0.01\pm 0.01\pm 0.00$	$308^{+8}_{-7}\pm 38$	347
	800–900	$17.7^{+2.6}_{-2.3}\pm 2.2$	$107\pm 1\pm 20$	$<0.01$	$125\pm 3\pm 21$	111
	900–1000	$6.0\pm 0.9\pm 1.3$	$39.4\pm 0.5\pm 8.5$	$0.01\pm 0.01\pm 0.00$	$45.4^{+1.1}_{-1.0}\pm 8.7$	39
	1000–1100	$3.3^{+1.1}_{-1.0}\pm 1.0$	$13.3\pm 0.2\pm 3.9$	$<0.01$	$16.6\pm 1.1\pm 4.1$	11
	$\ge 1100$	$0.31^{+0.09}_{-0.08}\pm 0.12$	$2.5\pm 0.0\pm 1.1$	$<0.01$	$2.8\pm 0.1\pm 1.1$	2
2-3j, 1b	200–300	$826^{+27}_{-26}\pm 54$	$1480^{+60}_{-50}\pm 100$	$38\pm 15\pm 12$	$2340\pm 60\pm 140$	2499
	300–400	$426^{+21}_{-20}\pm 28$	$994^{+38}_{-37}\pm 69$	$2.33\pm 0.26\pm 0.84$	$1422^{+43}_{-42}\pm 90$	1366
	400–600	$282^{+18}_{-17}\pm 20$	$788^{+30}_{-29}\pm 55$	$0.27\pm 0.06\pm 0.10$	$1071^{+35}_{-34}\pm 69$	1057
	600–800	$43.5^{+3.2}_{-3.1}\pm 6.5$	$129\pm 5\pm 12$	$<0.01$	$172\pm 6\pm 15$	225
	800–1000	$4.6\pm 0.7\pm 1.3$	$18.8\pm 0.7\pm 3.3$	$<0.01$	$23.4\pm 1.0\pm 3.6$	22
	$\ge 1000$	$0.34\pm 0.08\pm 0.14$	$2.05\pm 0.08\pm 0.90$	$<0.01$	$2.38\pm 0.11\pm 0.91$	1
2-3j, 2b	200–300	$105.1^{+9.2}_{-8.7}\pm 7.6$	$181^{+20}_{-18}\pm 15$	$3.8\pm 0.5\pm 1.3$	$290^{+22}_{-20}\pm 20$	316
	300–400	$55.0^{+6.7}_{-6.3}\pm 7.5$	$122^{+14}_{-12}\pm 10$	$0.27\pm 0.06\pm 0.10$	$177^{+15}_{-14}\pm 14$	159
	400–600	$36.5^{+4.6}_{-4.3}\pm 5.5$	$97^{+11}_{-10}\pm 8$	$0.08\pm 0.03\pm 0.03$	$133^{+12}_{-11}\pm 11$	107
	600–800	$4.7\pm 0.8\pm 1.3$	$15.8^{+1.8}_{-1.6}\pm 1.6$	$<0.01$	$20.6^{+1.9}_{-1.8}\pm 2.2$	21
	$\ge 800$	$0.59\pm 0.19\pm 0.24$	$2.56^{+0.29}_{-0.26}\pm 0.45$	$<0.01$	$3.14^{+0.35}_{-0.32}\pm 0.52$	1

**Table 17:** Predictions and observations for the 26 search regions with $575 \le H_{\mathrm {T}} < 1200\,\,\text {Ge}\text {V}$, and $2\le N_{\mathrm {j}} \le 6$ and $N_{{\text {b}}} \ge 3$, or $4\le N_{\mathrm {j}} \le 6$. For each of the background predictions, the first uncertainty listed is statistical (from the limited size of data control samples and Monte Carlo samples), and the second is systematic

$N_{\mathrm {j}}$, $N_{{\text {b}}}$	$M_{\mathrm {T2}}$ ($\text {Ge}\text {V}$)	Lost lepton	${\text {Z}} \rightarrow {{\upnu }} \bar{{{\upnu }}}$	Multijet	Total background	Data
$575 \le H_{\mathrm {T}} < 1200\,\,\text {Ge}\text {V}$:						
2-6j, $\ge 3$b	200–300	$299^{+17}_{-16}\pm 22$	$73^{+15}_{-13}\pm 10$	$6.2\pm 0.4\pm 2.1$	$379^{+22}_{-21}\pm 28$	345
	300–400	$100\pm 10\pm 7$	$43.5^{+8.8}_{-7.4}\pm 6.2$	$0.68\pm 0.09\pm 0.24$	$144^{+14}_{-12}\pm 11$	132
	400–600	$32.5^{+6.3}_{-5.6}\pm 2.5$	$31.2^{+6.3}_{-5.3}\pm 4.4$	$0.08\pm 0.03\pm 0.03$	$63.8^{+8.9}_{-7.7}\pm 5.8$	48
	600–800	$3.16^{+0.95}_{-0.90}\pm 0.68$	$5.4^{+1.1}_{-0.9}\pm 0.8$	$<0.01$	$8.6^{+1.4}_{-1.3}\pm 1.1$	4
	$\ge 800$	$0.10\pm 0.03\pm 0.04$	$0.71^{+0.14}_{-0.12}\pm 0.15$	$<0.01$	$0.81^{+0.15}_{-0.12}\pm 0.16$	0
4-6j, 0b	200–300	$6280\pm 70\pm 420$	$9470\pm 160\pm 650$	$360\pm 20\pm 110$	$16\,100\pm 180\pm 1000$	16 292
	300–400	$2700\pm 50\pm 180$	$5410\pm 90\pm 380$	$53\pm 1\pm 17$	$8160\pm 100\pm 520$	8330
	400–500	$927^{+28}_{-27}\pm 62$	$2420\pm 40\pm 180$	$7.7\pm 0.4\pm 2.4$	$3350\pm 50\pm 230$	3576
	500–600	$324^{+17}_{-16}\pm 22$	$1171^{+20}_{-19}\pm 100$	$1.46\pm 0.12\pm 0.46$	$1500\pm 30\pm 110$	1516
	600–700	$95.4^{+9.4}_{-8.7}\pm 6.4$	$413\pm 7\pm 47$	$0.33\pm 0.06\pm 0.10$	$509^{+12}_{-11}\pm 50$	543
	700–800	$35.6^{+5.0}_{-4.5}\pm 3.6$	$171\pm 3\pm 27$	$0.03\pm 0.02\pm 0.01$	$206^{+6}_{-5}\pm 27$	178
	800–900	$13.4^{+2.0}_{-1.8}\pm 1.6$	$64\pm 1\pm 11$	$0.02\pm 0.01\pm 0.01$	$77\pm 2\pm 11$	62
	900–1000	$4.39^{+0.78}_{-0.73}\pm 0.93$	$23.6\pm 0.4\pm 5.3$	$<0.01$	$28.0^{+0.9}_{-0.8}\pm 5.4$	20
	1000–1100	$0.64\pm 0.16\pm 0.20$	$6.3\pm 0.1\pm 2.0$	$<0.01$	$6.9\pm 0.2\pm 2.0$	3
	$\ge 1100$	$0.78\pm 0.58\pm 0.32$	$0.89^{+0.02}_{-0.01}\pm 0.40$	$<0.01$	$1.68\pm 0.58\pm 0.52$	1
4-6j, 1b	200–300	$2900\pm 50\pm 200$	$2220^{+80}_{-70}\pm 150$	$154\pm 16\pm 50$	$5270\pm 90\pm 330$	5335
	300–400	$1066\pm 29\pm 74$	$1267^{+44}_{-42}\pm 89$	$19.2\pm 0.9\pm 6.2$	$2350\pm 50\pm 150$	2547
	400–600	$504^{+22}_{-21}\pm 35$	$840^{+29}_{-28}\pm 61$	$2.98\pm 0.21\pm 0.93$	$1347^{+36}_{-35}\pm 88$	1284
	600–800	$35.3^{+5.9}_{-5.2}\pm 2.6$	$138\pm 5\pm 14$	$0.09\pm 0.03\pm 0.03$	$174^{+8}_{-7}\pm 16$	151
	800–1000	$3.89^{+0.83}_{-0.77}\pm 0.82$	$19.3^{+0.7}_{-0.6}\pm 4.3$	$0.01\pm 0.01\pm 0.00$	$23.2^{+1.1}_{-1.0}\pm 4.5$	18
	$\ge 1000$	$0.18\pm 0.07\pm 0.07$	$1.57\pm 0.05\pm 0.65$	$<0.01$	$1.75\pm 0.09\pm 0.65$	1
4-6j, 2b	200–300	$1500\pm 30\pm 100$	$473^{+36}_{-33}\pm 36$	$42\pm 2\pm 13$	$2020\pm 50\pm 130$	1968
	300–400	$508\pm 20\pm 35$	$270^{+20}_{-19}\pm 21$	$4.9\pm 0.3\pm 1.6$	$783^{+29}_{-28}\pm 50$	788
	400–600	$167\pm 12\pm 12$	$179^{+14}_{-13}\pm 14$	$0.57\pm 0.08\pm 0.18$	$346^{+18}_{-17}\pm 23$	354
	600–800	$11.9^{+1.3}_{-1.2}\pm 2.5$	$29.5^{+2.2}_{-2.1}\pm 3.5$	$0.02\pm 0.01\pm 0.01$	$41.4^{+2.6}_{-2.4}\pm 4.6$	37
	$\ge 800$	$0.91\pm 0.23\pm 0.37$	$4.4\pm 0.3\pm 1.8$	$<0.01$	$5.4\pm 0.4\pm 1.9$	7

**Table 18:** Predictions and observations for the 34 search regions with $575 \le H_{\mathrm {T}} < 1200\,\,\text {Ge}\text {V}$, and $7\le N_{\mathrm {j}} \le 9$, or $N_{\mathrm {j}} \ge 10$. For each of the background predictions, the first uncertainty listed is statistical (from the limited size of data control samples and Monte Carlo samples), and the second is systematic

$N_{\mathrm {j}}$, $N_{{\text {b}}}$	$M_{\mathrm {T2}}$ ($\text {Ge}\text {V}$)	Lost lepton	${\text {Z}} \rightarrow {{\upnu }} \bar{{{\upnu }}}$	Multijet	Total background	Data
$575 \le H_{\mathrm {T}} < 1200\,\,\text {Ge}\text {V}$:						
7-9j, 0b	200–300	$589^{+27}_{-26}\pm 39$	$573^{+47}_{-43}\pm 64$	$90\pm 10\pm 28$	$1252^{+55}_{-52}\pm 93$	1340
	300–400	$265^{+19}_{-18}\pm 18$	$279^{+23}_{-21}\pm 42$	$14.9\pm 0.5\pm 4.7$	$559^{+29}_{-28}\pm 51$	581
	400–600	$92^{+10}_{-9}\pm 6$	$159^{+13}_{-12}\pm 28$	$2.72\pm 0.18\pm 0.85$	$253^{+16}_{-15}\pm 30$	243
	600–800	$8.6\pm 1.2\pm 1.8$	$22.8^{+1.9}_{-1.7}\pm 6.4$	$0.10\pm 0.03\pm 0.03$	$31.6^{+2.2}_{-2.1}\pm 6.8$	32
	$\ge 800$	$0.51\pm 0.16\pm 0.21$	$3.0\pm 0.2\pm 1.3$	$<0.01$	$3.5\pm 0.3\pm 1.3$	2
7-9j, 1b	200–300	$733\pm 21\pm 52$	$278^{+28}_{-25}\pm 33$	$48\pm 3\pm 16$	$1059^{+35}_{-33}\pm 73$	1052
	300–400	$252^{+13}_{-12}\pm 18$	$135^{+14}_{-12}\pm 21$	$7.7\pm 0.4\pm 2.5$	$395^{+19}_{-17}\pm 32$	387
	400–600	$71.3^{+6.9}_{-6.5}\pm 5.2$	$77^{+8}_{-7}\pm 14$	$1.36\pm 0.13\pm 0.45$	$150\pm 10\pm 16$	131
	600–800	$4.26^{+0.73}_{-0.71}\pm 0.90$	$11.0^{+1.1}_{-1.0}\pm 3.1$	$0.03\pm 0.02\pm 0.01$	$15.3^{+1.3}_{-1.2}\pm 3.3$	20
	$\ge 800$	$0.11\pm 0.04\pm 0.05$	$1.48^{+0.15}_{-0.13}\pm 0.63$	$<0.01$	$1.60^{+0.15}_{-0.14}\pm 0.63$	1
7-9j, 2b	200–300	$675\pm 20\pm 51$	$82^{+8}_{-7}\pm 10$	$20.9\pm 3.0\pm 6.7$	$777^{+22}_{-21}\pm 56$	750
	300–400	$211\pm 11\pm 16$	$39.8^{+4.0}_{-3.6}\pm 6.4$	$2.42\pm 0.19\pm 0.79$	$253^{+12}_{-11}\pm 19$	259
	400–600	$55.4^{+5.5}_{-5.2}\pm 4.2$	$22.7^{+2.3}_{-2.1}\pm 4.2$	$0.50\pm 0.07\pm 0.16$	$78.6^{+5.9}_{-5.6}\pm 6.6$	72
	600–800	$3.00^{+0.63}_{-0.62}\pm 0.64$	$3.25^{+0.32}_{-0.30}\pm 0.93$	$0.01\pm 0.01\pm 0.01$	$6.3\pm 0.7\pm 1.2$	7
	$\ge 800$	$0.27\pm 0.20\pm 0.11$	$0.44\pm 0.04\pm 0.19$	$<0.01$	$0.71\pm 0.20\pm 0.22$	1
7-9j, 3b	200–300	$185\pm 8\pm 18$	$11.3^{+1.1}_{-1.0}\pm 1.9$	$3.6\pm 0.2\pm 1.2$	$200\pm 8\pm 18$	184
	300–400	$52.0\pm 3.8\pm 5.0$	$5.5\pm 0.5\pm 1.2$	$0.72\pm 0.12\pm 0.26$	$58.3^{+3.9}_{-3.8}\pm 5.3$	59
	400–600	$13.6\pm 1.8\pm 1.3$	$3.13^{+0.31}_{-0.29}\pm 0.82$	$0.05\pm 0.02\pm 0.02$	$16.8\pm 1.8\pm 1.6$	14
	$\ge 600$	$0.49\pm 0.21\pm 0.20$	$0.51\pm 0.05\pm 0.21$	$<0.01$	$1.00\pm 0.21\pm 0.29$	2
7-9j, $\ge 4$b	200–300	$38.8\pm 3.1\pm 7.4$	$2.01^{+0.20}_{-0.18}\pm 0.71$	$0.55\pm 0.08\pm 0.19$	$41.3^{+3.2}_{-3.1}\pm 7.4$	38
	300–400	$14.5^{+2.0}_{-1.9}\pm 2.8$	$0.98^{+0.10}_{-0.09}\pm 0.43$	$0.06\pm 0.02\pm 0.02$	$15.6^{+2.0}_{-1.9}\pm 2.8$	16
	$\ge 400$	$3.75^{+0.98}_{-0.97}\pm 0.70$	$0.65\pm 0.06\pm 0.35$	$<0.01$	$4.40^{+0.98}_{-0.97}\pm 0.79$	3
$\ge 10$j, 0b	200–300	$11.5\pm 1.6\pm 1.0$	$4.4^{+0.4}_{-0.3}\pm 2.3$	$3.1\pm 0.8\pm 1.1$	$19.0\pm 1.8\pm 2.8$	27
	300–500	$5.6\pm 1.0\pm 0.5$	$3.0\pm 0.2\pm 1.7$	$0.55\pm 0.08\pm 0.20$	$9.1\pm 1.0\pm 1.8$	4
	$\ge 500$	$0.30\pm 0.11\pm 0.12$	$0.44^{+0.04}_{-0.03}\pm 0.24$	$0.02\pm 0.01\pm 0.01$	$0.76\pm 0.11\pm 0.27$	3
$\ge 10$j, 1b	200–300	$21.0\pm 1.8\pm 1.6$	$3.5\pm 0.3\pm 1.9$	$1.92\pm 0.18\pm 0.72$	$26.4\pm 1.8\pm 2.7$	32
	300–500	$7.7\pm 1.0\pm 0.6$	$2.4\pm 0.2\pm 1.4$	$0.45\pm 0.07\pm 0.17$	$10.5\pm 1.1\pm 1.6$	15
	$\ge 500$	$0.83^{+0.42}_{-0.41}\pm 0.07$	$0.36^{+0.04}_{-0.03}\pm 0.20$	$0.02\pm 0.01\pm 0.01$	$1.20^{+0.42}_{-0.41}\pm 0.22$	0
$\ge 10$j, 2b	200–300	$21.8\pm 1.8\pm 1.6$	$1.05\pm 0.10\pm 0.66$	$0.64\pm 0.08\pm 0.24$	$23.5\pm 1.8\pm 1.8$	26
	300–500	$8.8\pm 1.2\pm 0.6$	$0.69^{+0.07}_{-0.06}\pm 0.45$	$0.16\pm 0.04\pm 0.06$	$9.6^{+1.3}_{-1.2}\pm 0.8$	9
	$\ge 500$	$0.22\pm 0.13\pm 0.02$	$0.10\pm 0.01\pm 0.06$	$<0.01$	$0.32\pm 0.13\pm 0.07$	0
$\ge 10$j, 3b	200–300	$9.9\pm 1.3\pm 1.2$	$0.25\pm 0.02\pm 0.20$	$0.29\pm 0.05\pm 0.12$	$10.4\pm 1.3\pm 1.2$	14
	$\ge 300$	$1.59\pm 0.50\pm 0.18$	$0.19\pm 0.02\pm 0.16$	$0.02\pm 0.01\pm 0.01$	$1.80\pm 0.50\pm 0.25$	2
$\ge 10$j, $\ge 4$b	$\ge 200$	$3.9\pm 1.2\pm 0.8$	$0.00^{+0.17}_{-0.00}\pm 0.00$	$0.05\pm 0.02\pm 0.02$	$4.0\pm 1.2\pm 0.8$	6

**Table 19:** Predictions and observations for the 17 search regions with $1200 \le H_{\mathrm {T}} < 1500\,\,\text {Ge}\text {V}$ and $2\le N_{\mathrm {j}} \le 3$. For each of the background predictions, the first uncertainty listed is statistical (from the limited size of data control samples and Monte Carlo samples), and the second is systematic

$N_{\mathrm {j}}$, $N_{{\text {b}}}$	$M_{\mathrm {T2}}$ ($\text {Ge}\text {V}$)	Lost lepton	${\text {Z}} \rightarrow {{\upnu }} \bar{{{\upnu }}}$	Multijet	Total background	Data
$1200 \le H_{\mathrm {T}} < 1500\,\,\text {Ge}\text {V}$:						
2-3j, 0b	200–400	$315\pm 15\pm 21$	$656^{+51}_{-47}\pm 73$	$39\pm 16\pm 12$	$1009^{+55}_{-52}\pm 85$	1128
	400–600	$43.0^{+5.2}_{-4.7}\pm 4.9$	$185^{+14}_{-13}\pm 30$	$0.03\pm 0.02\pm 0.01$	$228^{+15}_{-14}\pm 31$	207
	600–800	$14.1^{+2.1}_{-2.0}\pm 1.7$	$64\pm 5\pm 17$	$<0.01$	$78\pm 5\pm 17$	83
	800–1000	$6.4^{+1.1}_{-1.0}\pm 1.3$	$32.5^{+2.5}_{-2.3}\pm 7.6$	$<0.01$	$38.9^{+2.7}_{-2.5}\pm 7.8$	36
	1000–1200	$3.23^{+0.61}_{-0.59}\pm 0.99$	$17.5\pm 1.3\pm 5.2$	$<0.01$	$20.7^{+1.5}_{-1.4}\pm 5.3$	19
	$\ge 1200$	$0.87^{+0.14}_{-0.13}\pm 0.35$	$6.0^{+0.5}_{-0.4}\pm 2.6$	$<0.01$	$6.9\pm 0.5\pm 2.6$	4
2-3j, 1b	200–400	$61.5^{+7.2}_{-6.5}\pm 4.2$	$78^{+19}_{-16}\pm 10$	$9.7\pm 0.7\pm 3.0$	$149^{+21}_{-17}\pm 12$	157
	400–600	$10.1\pm 1.4\pm 1.0$	$21.9^{+5.4}_{-4.4}\pm 3.8$	$0.03\pm 0.02\pm 0.01$	$32.0^{+5.6}_{-4.6}\pm 4.1$	27
	600–800	$2.36^{+0.36}_{-0.35}\pm 0.41$	$7.5^{+1.9}_{-1.5}\pm 2.0$	$<0.01$	$9.8^{+1.9}_{-1.6}\pm 2.1$	9
	800–1000	$0.78^{+0.16}_{-0.15}\pm 0.19$	$3.84^{+0.95}_{-0.78}\pm 0.93$	$<0.01$	$4.62^{+0.97}_{-0.79}\pm 0.96$	6
	1000–1200	$0.43\pm 0.08\pm 0.14$	$2.13^{+0.53}_{-0.43}\pm 0.64$	$<0.01$	$2.56^{+0.54}_{-0.44}\pm 0.66$	2
	$\ge 1200$	$0.14^{+0.05}_{-0.04}\pm 0.06$	$0.71^{+0.18}_{-0.14}\pm 0.31$	$<0.01$	$0.86^{+0.18}_{-0.15}\pm 0.31$	0
2-3j, 2b	200–400	$4.8^{+2.0}_{-1.6}\pm 0.3$	$11^{+11}_{-6}\pm 2$	$1.38\pm 0.13\pm 0.43$	$18^{+11}_{-6}\pm 2$	18
	400–600	$0.61^{+0.30}_{-0.25}\pm 0.07$	$3.2^{+3.1}_{-1.7}\pm 0.7$	$<0.01$	$3.8^{+3.1}_{-1.8}\pm 0.7$	5
	600–800	$0.21^{+0.11}_{-0.09}\pm 0.04$	$1.1^{+1.1}_{-0.6}\pm 0.4$	$<0.01$	$1.3^{+1.1}_{-0.6}\pm 0.4$	2
	800–1000	$0.07^{+0.04}_{-0.03}\pm 0.02$	$0.56^{+0.55}_{-0.31}\pm 0.18$	$<0.01$	$0.63^{+0.55}_{-0.31}\pm 0.18$	1
	$\ge 1000$	$0.03\pm 0.02\pm 0.01$	$0.42^{+0.41}_{-0.23}\pm 0.18$	$<0.01$	$0.46^{+0.41}_{-0.23}\pm 0.18$	1

**Table 20:** Predictions and observations for the 20 search regions with $1200 \le H_{\mathrm {T}} < 1500\,\,\text {Ge}\text {V}$, and $2\le N_{\mathrm {j}} \le 6$ and $N_{{\text {b}}} \ge 3$, or $4\le N_{\mathrm {j}} \le 6$. For each of the background predictions, the first uncertainty listed is statistical (from the limited size of data control samples and Monte Carlo samples), and the second is systematic

$N_{\mathrm {j}}$, $N_{{\text {b}}}$	$M_{\mathrm {T2}}$ ($\text {Ge}\text {V}$)	Lost lepton	${\text {Z}} \rightarrow {{\upnu }} \bar{{{\upnu }}}$	Multijet	Total background	Data
$1200 \le H_{\mathrm {T}} < 1500\,\,\text {Ge}\text {V}$:						
2-6j, $\ge 3$b	200–400	$22.6^{+4.7}_{-4.2}\pm 1.8$	$0.0^{+6.6}_{-0.0}\pm 0.0$	$4.4\pm 0.2\pm 1.5$	$27.0^{+8.1}_{-4.2}\pm 2.4$	25
	400–600	$1.58^{+0.51}_{-0.48}\pm 0.34$	$0.0^{+1.6}_{-0.0}\pm 0.0$	$0.02\pm 0.01\pm 0.01$	$1.6^{+1.7}_{-0.5}\pm 0.3$	3
	$\ge 600$	$0.47^{+0.27}_{-0.26}\pm 0.19$	$0.00^{+0.94}_{-0.00}\pm 0.00$	$<0.01$	$0.47^{+0.98}_{-0.26}\pm 0.19$	4
4-6j, 0b	200–400	$606^{+21}_{-20}\pm 41$	$909^{+63}_{-59}\pm 90$	$208\pm 12\pm 64$	$1720^{+70}_{-60}\pm 130$	1768
	400–600	$84.3^{+7.4}_{-6.9}\pm 5.8$	$234^{+16}_{-15}\pm 34$	$0.88\pm 0.09\pm 0.27$	$319^{+18}_{-17}\pm 36$	301
	600–800	$21.1^{+3.2}_{-2.9}\pm 2.3$	$75\pm 5\pm 17$	$0.06\pm 0.02\pm 0.02$	$96\pm 6\pm 17$	99
	800–1000	$7.6^{+1.2}_{-1.1}\pm 1.1$	$35.2^{+2.4}_{-2.3}\pm 8.0$	$0.01\pm 0.01\pm 0.00$	$42.7^{+2.7}_{-2.5}\pm 8.2$	41
	1000–1200	$2.23^{+0.36}_{-0.33}\pm 0.61$	$14.1^{+1.0}_{-0.9}\pm 4.2$	$<0.01$	$16.3\pm 1.0\pm 4.2$	15
	$\ge 1200$	$0.47^{+0.10}_{-0.09}\pm 0.19$	$3.0\pm 0.2\pm 1.3$	$<0.01$	$3.5\pm 0.2\pm 1.3$	5
4-6j, 1b	200–400	$278^{+15}_{-14}\pm 20$	$254^{+33}_{-30}\pm 28$	$97\pm 2\pm 30$	$629^{+36}_{-33}\pm 50$	579
	400–600	$30.3^{+4.0}_{-3.7}\pm 2.7$	$65^{+9}_{-8}\pm 10$	$0.33\pm 0.06\pm 0.10$	$96^{+9}_{-8}\pm 11$	79
	600–800	$8.2^{+1.4}_{-1.3}\pm 1.0$	$21.0^{+2.8}_{-2.5}\pm 4.8$	$0.02\pm 0.01\pm 0.01$	$29.2^{+3.1}_{-2.8}\pm 5.0$	16
	800–1000	$2.36^{+0.56}_{-0.54}\pm 0.50$	$9.8^{+1.3}_{-1.1}\pm 2.3$	$0.01\pm 0.01\pm 0.00$	$12.2^{+1.4}_{-1.3}\pm 2.4$	9
	1000–1200	$1.00\pm 0.24\pm 0.31$	$4.0\pm 0.5\pm 1.2$	$<0.01$	$5.0^{+0.6}_{-0.5}\pm 1.2$	6
	$\ge 1200$	$0.07\pm 0.02\pm 0.03$	$0.86^{+0.11}_{-0.10}\pm 0.37$	$<0.01$	$0.92^{+0.11}_{-0.10}\pm 0.37$	1
4-6j, 2b	200–400	$120.4^{+9.1}_{-8.7}\pm 9.8$	$45^{+18}_{-13}\pm 5$	$26.0\pm 0.6\pm 8.1$	$191^{+20}_{-16}\pm 15$	194
	400–600	$11.9\pm 1.4\pm 1.5$	$11.5^{+4.6}_{-3.4}\pm 1.8$	$0.11\pm 0.03\pm 0.04$	$23.4^{+4.8}_{-3.7}\pm 2.6$	27
	600–800	$3.49\pm 0.83\pm 0.75$	$3.7^{+1.5}_{-1.1}\pm 1.0$	$<0.01$	$7.2^{+1.7}_{-1.4}\pm 1.3$	7
	800–1000	$0.66\pm 0.16\pm 0.20$	$1.73^{+0.69}_{-0.51}\pm 0.48$	$<0.01$	$2.38^{+0.71}_{-0.54}\pm 0.53$	3
	$\ge 1000$	$0.15\pm 0.04\pm 0.06$	$0.84^{+0.34}_{-0.25}\pm 0.36$	$<0.01$	$1.00^{+0.34}_{-0.25}\pm 0.36$	0

**Table 21:** Predictions and observations for the 31 search regions with $1200 \le H_{\mathrm {T}} < 1500\,\,\text {Ge}\text {V}$, and $7\le N_{\mathrm {j}} \le 9$, or $N_{\mathrm {j}} \ge 10$. For each of the background predictions, the first uncertainty listed is statistical (from the limited size of data control samples and Monte Carlo samples), and the second is systematic

$N_{\mathrm {j}}$, $N_{{\text {b}}}$	$M_{\mathrm {T2}}$ ($\text {Ge}\text {V}$)	Lost lepton	${\text {Z}} \rightarrow {{\upnu }} \bar{{{\upnu }}}$	Multijet	Total background	Data
$1200 \le H_{\mathrm {T}} < 1500\,\,\text {Ge}\text {V}$:						
7-9j, 0b	200–400	$120.4^{+9.8}_{-9.2}\pm 9.0$	$108^{+26}_{-21}\pm 21$	$91\pm 3\pm 29$	$319^{+28}_{-24}\pm 38$	379
	400–600	$16.5^{+1.9}_{-1.8}\pm 2.0$	$25.8^{+6.3}_{-5.1}\pm 5.7$	$0.80\pm 0.09\pm 0.25$	$43.1^{+6.5}_{-5.4}\pm 6.3$	45
	600–800	$2.94\pm 0.42\pm 0.63$	$8.6^{+2.1}_{-1.7}\pm 2.1$	$0.06\pm 0.02\pm 0.02$	$11.6^{+2.1}_{-1.8}\pm 2.2$	17
	800–1000	$0.77^{+0.14}_{-0.13}\pm 0.24$	$2.90^{+0.70}_{-0.58}\pm 1.00$	$0.01\pm 0.01\pm 0.00$	$3.7^{+0.7}_{-0.6}\pm 1.0$	3
	$\ge 1000$	$0.11\pm 0.03\pm 0.05$	$1.09^{+0.26}_{-0.22}\pm 0.50$	$<0.01$	$1.21^{+0.27}_{-0.22}\pm 0.50$	0
7-9j, 1b	200–400	$133.8^{+8.0}_{-7.7}\pm 9.8$	$36^{+13}_{-10}\pm 8$	$58\pm 2\pm 18$	$228^{+15}_{-13}\pm 23$	247
	400–600	$16.6^{+2.9}_{-2.7}\pm 1.3$	$8.7^{+3.2}_{-2.4}\pm 2.1$	$0.46\pm 0.07\pm 0.14$	$25.8^{+4.3}_{-3.6}\pm 2.7$	23
	600–800	$1.83^{+0.43}_{-0.41}\pm 0.28$	$2.9^{+1.1}_{-0.8}\pm 0.8$	$0.03\pm 0.02\pm 0.01$	$4.8^{+1.1}_{-0.9}\pm 0.8$	7
	800–1000	$0.65^{+0.24}_{-0.23}\pm 0.18$	$0.95^{+0.34}_{-0.26}\pm 0.34$	$0.02\pm 0.01\pm 0.01$	$1.62^{+0.42}_{-0.35}\pm 0.39$	2
	$\ge 1000$	$0.22\pm 0.19\pm 0.09$	$0.36^{+0.13}_{-0.10}\pm 0.17$	$<0.01$	$0.58^{+0.23}_{-0.21}\pm 0.19$	0
7-9j, 2b	200–400	$124.0^{+7.6}_{-7.4}\pm 9.1$	$9.9^{+3.6}_{-2.7}\pm 2.5$	$21.4\pm 0.5\pm 6.9$	$155\pm 8\pm 12$	162
	400–600	$15.0^{+2.8}_{-2.6}\pm 1.3$	$2.41^{+0.87}_{-0.66}\pm 0.67$	$0.12\pm 0.03\pm 0.04$	$17.5^{+3.0}_{-2.7}\pm 1.5$	18
	600–800	$2.47^{+0.78}_{-0.76}\pm 0.53$	$0.81^{+0.29}_{-0.22}\pm 0.26$	$0.01\pm 0.01\pm 0.00$	$3.29^{+0.83}_{-0.79}\pm 0.60$	1
	$\ge 800$	$0.24\pm 0.11\pm 0.10$	$0.36^{+0.13}_{-0.10}\pm 0.16$	$<0.01$	$0.60^{+0.17}_{-0.15}\pm 0.19$	1
7-9j, 3b	200–400	$30.0\pm 2.6\pm 3.2$	$1.89^{+0.68}_{-0.52}\pm 0.64$	$5.0\pm 0.3\pm 1.8$	$36.9^{+2.7}_{-2.6}\pm 3.8$	46
	400–600	$4.1^{+1.1}_{-1.0}\pm 0.6$	$0.45^{+0.16}_{-0.12}\pm 0.18$	$0.02\pm 0.01\pm 0.01$	$4.6^{+1.1}_{-1.0}\pm 0.6$	2
	$\ge 600$	$0.92^{+0.50}_{-0.49}\pm 0.38$	$0.23^{+0.08}_{-0.06}\pm 0.11$	$<0.01$	$1.15\pm 0.50\pm 0.40$	1
7-9j, $\ge 4$b	200–400	$9.1\pm 1.6\pm 1.8$	$0.26^{+0.10}_{-0.07}\pm 0.23$	$0.88\pm 0.10\pm 0.32$	$10.3\pm 1.6\pm 1.9$	9
	$\ge 400$	$0.44^{+0.24}_{-0.23}\pm 0.08$	$0.10^{+0.04}_{-0.03}\pm 0.09$	$<0.01$	$0.53\pm 0.24\pm 0.12$	0
$\ge 10$j, 0b	200–400	$7.7^{+1.2}_{-1.1}\pm 0.8$	$2.7^{+0.6}_{-0.5}\pm 2.8$	$8.3\pm 0.9\pm 3.0$	$18.7^{+1.6}_{-1.5}\pm 4.1$	17
	400–600	$1.00\pm 0.32\pm 0.22$	$0.56^{+0.13}_{-0.11}\pm 0.62$	$0.11\pm 0.03\pm 0.04$	$1.66^{+0.35}_{-0.34}\pm 0.66$	1
	$\ge 600$	$0.10^{+0.35}_{-0.04}\pm 0.04$	$0.14^{+0.08}_{-0.03}\pm 0.14$	$0.01\pm 0.01\pm 0.00$	$0.24^{+0.36}_{-0.05}\pm 0.15$	0
$\ge 10$j, 1b	200–400	$15.2\pm 1.8\pm 1.4$	$1.1^{+0.4}_{-0.3}\pm 1.2$	$5.3\pm 0.2\pm 1.9$	$21.6^{+1.9}_{-1.8}\pm 2.7$	22
	400–600	$1.27^{+0.38}_{-0.36}\pm 0.11$	$0.22^{+0.08}_{-0.06}\pm 0.26$	$0.05\pm 0.02\pm 0.02$	$1.55^{+0.39}_{-0.37}\pm 0.29$	6
	$\ge 600$	$0.03\pm 0.02\pm 0.01$	$0.05^{+0.10}_{-0.01}\pm 0.05$	$<0.01$	$0.07^{+0.11}_{-0.02}\pm 0.05$	0
$\ge 10$j, 2b	200–400	$16.9\pm 1.8\pm 1.5$	$0.44^{+0.16}_{-0.12}\pm 0.50$	$2.7\pm 0.2\pm 1.0$	$20.1\pm 1.8\pm 1.9$	16
	400–600	$2.62^{+0.71}_{-0.68}\pm 0.30$	$0.09\pm 0.03\pm 0.11$	$0.01\pm 0.01\pm 0.00$	$2.73^{+0.71}_{-0.68}\pm 0.32$	2
	$\ge 600$	$0.23\pm 0.15\pm 0.10$	$0.02^{+0.08}_{-0.01}\pm 0.02$	$<0.01$	$0.25^{+0.17}_{-0.15}\pm 0.10$	0
$\ge 10$j, 3b	200–400	$5.58^{+0.86}_{-0.85}\pm 0.61$	$0.12^{+0.11}_{-0.03}\pm 0.16$	$1.04\pm 0.10\pm 0.42$	$6.74^{+0.87}_{-0.86}\pm 0.76$	6
	$\ge 400$	$0.51\pm 0.22\pm 0.06$	$0.03^{+0.11}_{-0.01}\pm 0.04$	$<0.01$	$0.54^{+0.25}_{-0.22}\pm 0.08$	0
$\ge 10$j, $\ge 4$b	$\ge 200$	$2.59\pm 0.82\pm 0.62$	$0.10^{+0.13}_{-0.03}\pm 0.13$	$0.31\pm 0.06\pm 0.13$	$3.00^{+0.83}_{-0.82}\pm 0.65$	7

**Table 22:** Predictions and observations for the 30 search regions with $H_{\mathrm {T}} \ge 1500\,\,\text {Ge}\text {V}$, and $2 \le N_{\mathrm {j}} \le 3$, $2 \le N_{\mathrm {j}} \le 6$ and $N_{{\text {b}}} \ge 3$, or $4 \le N_{\mathrm {j}} \le 6$. For each of the background predictions, the first uncertainty listed is statistical (from the limited size of data control samples and Monte Carlo samples), and the second is systematic

$N_{\mathrm {j}}$, $N_{{\text {b}}}$	$M_{\mathrm {T2}}$ ($\text {Ge}\text {V}$)	Lost lepton	${\text {Z}} \rightarrow {{\upnu }} \bar{{{\upnu }}}$	Multijet	Total background	Data
$H_{\mathrm {T}} \ge 1500\,\,\text {Ge}\text {V}$:						
2-3j, 0b	400–600	$27.2^{+4.4}_{-3.9}\pm 2.5$	$150^{+14}_{-13}\pm 19$	$0.16\pm 0.04\pm 0.05$	$177^{+15}_{-13}\pm 20$	125
	600–800	$7.8^{+1.4}_{-1.2}\pm 0.8$	$38.7^{+3.6}_{-3.3}\pm 8.4$	$<0.01$	$46.5^{+3.9}_{-3.6}\pm 8.6$	37
	800–1000	$2.29^{+0.39}_{-0.34}\pm 0.35$	$17.2^{+1.6}_{-1.5}\pm 3.4$	$<0.01$	$19.5^{+1.7}_{-1.5}\pm 3.4$	19
	1000–1200	$1.20^{+0.21}_{-0.19}\pm 0.26$	$9.0\pm 0.8\pm 1.8$	$<0.01$	$10.2^{+0.9}_{-0.8}\pm 1.9$	14
	1200–1400	$0.80^{+0.16}_{-0.14}\pm 0.22$	$4.9^{+0.5}_{-0.4}\pm 1.3$	$<0.01$	$5.7^{+0.5}_{-0.4}\pm 1.4$	4
	1400–1800	$0.43^{+0.09}_{-0.08}\pm 0.15$	$2.80^{+0.26}_{-0.24}\pm 0.98$	$<0.01$	$3.23^{+0.28}_{-0.26}\pm 0.99$	3
	$\ge 1800$	$0.05\pm 0.02\pm 0.02$	$0.41^{+0.04}_{-0.03}\pm 0.19$	$<0.01$	$0.46\pm 0.04\pm 0.19$	0
2-3j, 1b	400–600	$5.2^{+1.1}_{-1.0}\pm 0.6$	$13.4^{+4.9}_{-3.7}\pm 1.9$	$0.09\pm 0.03\pm 0.03$	$18.7^{+5.0}_{-3.8}\pm 2.1$	23
	600–800	$1.52^{+0.43}_{-0.41}\pm 0.27$	$3.5^{+1.3}_{-1.0}\pm 1.0$	$<0.01$	$5.0^{+1.3}_{-1.0}\pm 1.0$	3
	800–1000	$0.38\pm 0.09\pm 0.10$	$1.53^{+0.55}_{-0.42}\pm 0.35$	$<0.01$	$1.90^{+0.56}_{-0.43}\pm 0.37$	3
	1000–1200	$0.10\pm 0.03\pm 0.03$	$0.81^{+0.29}_{-0.22}\pm 0.24$	$<0.01$	$0.91^{+0.29}_{-0.22}\pm 0.24$	4
	$\ge 1200$	$0.19\pm 0.06\pm 0.08$	$0.73^{+0.26}_{-0.20}\pm 0.31$	$<0.01$	$0.92^{+0.27}_{-0.21}\pm 0.32$	0
2-3j, 2b	$\ge 400$	$0.63^{+0.49}_{-0.36}\pm 0.26$	$0.0^{+3.0}_{-0.0}\pm 0.0$	$<0.01$	$0.6^{+3.0}_{-0.4}\pm 0.3$	2
2-6j, $\ge 3$b	400–600	$1.72^{+0.73}_{-0.68}\pm 0.42$	$1.1^{+2.4}_{-0.9}\pm 0.3$	$0.03\pm 0.02\pm 0.01$	$2.8^{+2.5}_{-1.1}\pm 0.6$	1
	$\ge 600$	$0.37^{+0.19}_{-0.18}\pm 0.16$	$0.5^{+1.2}_{-0.4}\pm 0.2$	$<0.01$	$0.9^{+1.2}_{-0.5}\pm 0.2$	0
4-6j, 0b	400–600	$46.4^{+5.6}_{-5.1}\pm 3.6$	$176^{+15}_{-14}\pm 23$	$1.62\pm 0.13\pm 0.46$	$224^{+16}_{-15}\pm 24$	207
	600–800	$10.6^{+2.3}_{-1.9}\pm 1.2$	$45.5^{+4.0}_{-3.7}\pm 9.9$	$0.07\pm 0.03\pm 0.02$	$56^{+5}_{-4}\pm 10$	62
	800–1000	$4.5^{+1.1}_{-1.0}\pm 0.5$	$20.3^{+1.8}_{-1.6}\pm 3.9$	$<0.01$	$24.8^{+2.1}_{-1.9}\pm 4.1$	31
	1000–1200	$1.35^{+0.30}_{-0.26}\pm 0.24$	$10.6\pm 0.9\pm 2.1$	$<0.01$	$11.9^{+1.0}_{-0.9}\pm 2.2$	12
	1200–1400	$0.89^{+0.27}_{-0.25}\pm 0.23$	$5.7\pm 0.5\pm 1.5$	$<0.01$	$6.6^{+0.6}_{-0.5}\pm 1.6$	9
	1400–1600	$0.20\pm 0.05\pm 0.07$	$2.64^{+0.23}_{-0.21}\pm 0.92$	$<0.01$	$2.84^{+0.24}_{-0.22}\pm 0.92$	3
	$\ge 1600$	$0.09\pm 0.03\pm 0.04$	$1.18\pm 0.10\pm 0.51$	$<0.01$	$1.27^{+0.11}_{-0.10}\pm 0.51$	2
4-6j, 1b	400–600	$21.0^{+3.7}_{-3.3}\pm 2.0$	$32.6^{+7.0}_{-5.8}\pm 5.5$	$0.81\pm 0.09\pm 0.23$	$54.5^{+7.9}_{-6.7}\pm 6.3$	72
	600–800	$4.79^{+0.91}_{-0.83}\pm 0.62$	$8.4^{+1.8}_{-1.5}\pm 2.3$	$0.02\pm 0.01\pm 0.01$	$13.2^{+2.0}_{-1.7}\pm 2.5$	20
	800–1000	$1.27^{+0.26}_{-0.24}\pm 0.27$	$3.71^{+0.79}_{-0.66}\pm 0.92$	$0.03\pm 0.02\pm 0.01$	$5.01^{+0.84}_{-0.71}\pm 0.97$	8
	1000–1400	$0.89^{+0.21}_{-0.20}\pm 0.28$	$3.00^{+0.64}_{-0.54}\pm 0.93$	$<0.01$	$3.89^{+0.68}_{-0.57}\pm 0.98$	6
	$\ge 1400$	$0.40^{+0.34}_{-0.33}\pm 0.16$	$0.72^{+0.15}_{-0.13}\pm 0.31$	$<0.01$	$1.12^{+0.37}_{-0.36}\pm 0.36$	3
4-6j, 2b	400–600	$7.2^{+1.2}_{-1.1}\pm 1.1$	$4.3^{+2.9}_{-1.9}\pm 1.4$	$0.17\pm 0.04\pm 0.05$	$11.7^{+3.2}_{-2.2}\pm 1.9$	11
	600–800	$1.66^{+0.41}_{-0.40}\pm 0.46$	$1.12^{+0.76}_{-0.48}\pm 0.55$	$0.01\pm 0.01\pm 0.00$	$2.79^{+0.86}_{-0.63}\pm 0.73$	3
	$\ge 800$	$0.32\pm 0.13\pm 0.13$	$0.99^{+0.67}_{-0.43}\pm 0.52$	$<0.01$	$1.31^{+0.68}_{-0.45}\pm 0.54$	4

**Table 23:** Predictions and observations for the 21 search regions with $H_{\mathrm {T}} \ge 1500\,\,\text {Ge}\text {V}$, and $7\le N_{\mathrm {j}} \le 9$, or $N_{\mathrm {j}} \ge 10$. For each of the background predictions, the first uncertainty listed is statistical (from the limited size of data control samples and Monte Carlo samples), and the second is systematic

$N_{\mathrm {j}}$, $N_{{\text {b}}}$	$M_{\mathrm {T2}}$ ($\text {Ge}\text {V}$)	Lost lepton	${\text {Z}} \rightarrow {{\upnu }} \bar{{{\upnu }}}$	Multijet	Total background	Data
$H_{\mathrm {T}} \ge 1500\,\,\text {Ge}\text {V}$:						
7-9j, 0b	400–600	$14.3^{+1.8}_{-1.7}\pm 1.7$	$32.3^{+7.5}_{-6.2}\pm 4.3$	$1.50\pm 0.13\pm 0.44$	$48.1^{+7.7}_{-6.4}\pm 5.0$	36
	600–800	$3.77^{+0.56}_{-0.55}\pm 0.69$	$8.3^{+1.9}_{-1.6}\pm 2.2$	$0.18\pm 0.04\pm 0.05$	$12.3^{+2.0}_{-1.7}\pm 2.3$	9
	800–1000	$1.16^{+0.18}_{-0.17}\pm 0.30$	$3.70^{+0.86}_{-0.71}\pm 0.83$	$0.01\pm 0.01\pm 0.00$	$4.86^{+0.88}_{-0.73}\pm 0.90$	6
	1000–1400	$0.58\pm 0.11\pm 0.19$	$2.96^{+0.69}_{-0.57}\pm 0.86$	$0.01\pm 0.01\pm 0.00$	$3.55^{+0.69}_{-0.58}\pm 0.89$	4
	$\ge 1400$	$0.05\pm 0.01\pm 0.02$	$0.71^{+0.17}_{-0.14}\pm 0.30$	$<0.01$	$0.76^{+0.17}_{-0.14}\pm 0.30$	2
7-9j, 1b	400–600	$12.8^{+2.5}_{-2.3}\pm 1.6$	$9.2^{+4.2}_{-3.0}\pm 1.4$	$0.82\pm 0.09\pm 0.24$	$22.9^{+4.9}_{-3.8}\pm 2.3$	25
	600–800	$3.49^{+0.94}_{-0.89}\pm 0.76$	$2.4^{+1.1}_{-0.8}\pm 1.0$	$0.06\pm 0.02\pm 0.02$	$5.9^{+1.4}_{-1.2}\pm 1.2$	7
	$\ge 800$	$1.09^{+0.34}_{-0.32}\pm 0.45$	$2.10^{+0.96}_{-0.69}\pm 0.93$	$<0.01$	$3.2^{+1.0}_{-0.8}\pm 1.0$	2
7-9j, 2b	400–600	$8.1^{+1.8}_{-1.6}\pm 1.0$	$2.4^{+1.1}_{-0.8}\pm 0.4$	$0.35\pm 0.06\pm 0.10$	$10.9^{+2.1}_{-1.8}\pm 1.2$	10
	600–800	$1.78^{+0.54}_{-0.52}\pm 0.40$	$0.62^{+0.28}_{-0.20}\pm 0.25$	$0.02\pm 0.01\pm 0.01$	$2.41^{+0.61}_{-0.56}\pm 0.49$	5
	$\ge 800$	$0.40^{+0.19}_{-0.18}\pm 0.17$	$0.55^{+0.25}_{-0.18}\pm 0.25$	$0.01\pm 0.01\pm 0.00$	$0.96^{+0.31}_{-0.26}\pm 0.30$	0
7-9j, 3b	400–800	$2.40^{+0.74}_{-0.72}\pm 0.29$	$0.32^{+0.15}_{-0.10}\pm 0.12$	$0.10\pm 0.03\pm 0.03$	$2.82^{+0.76}_{-0.72}\pm 0.32$	2
	$\ge 800$	$0.16\pm 0.09\pm 0.07$	$0.08^{+0.04}_{-0.03}\pm 0.04$	$<0.01$	$0.24\pm 0.09\pm 0.08$	0
7-9j, $\ge 4$b	$\ge 400$	$0.52^{+0.23}_{-0.22}\pm 0.08$	$0.07^{+0.03}_{-0.02}\pm 0.06$	$0.02\pm 0.01\pm 0.01$	$0.61^{+0.23}_{-0.22}\pm 0.10$	1
$\ge 10$j, 0b	400–800	$1.41\pm 0.38\pm 0.33$	$1.52^{+0.35}_{-0.29}\pm 0.34$	$0.23\pm 0.05\pm 0.08$	$3.17^{+0.52}_{-0.48}\pm 0.49$	11
	$\ge 800$	$0.05\pm 0.02\pm 0.02$	$0.37^{+0.09}_{-0.07}\pm 0.17$	$0.01\pm 0.01\pm 0.00$	$0.43^{+0.09}_{-0.08}\pm 0.17$	0
$\ge 10$j, 1b	400–800	$2.16^{+0.71}_{-0.69}\pm 0.25$	$0.56^{+0.25}_{-0.18}\pm 0.16$	$0.14\pm 0.04\pm 0.05$	$2.85^{+0.76}_{-0.71}\pm 0.31$	3
	$\ge 800$	$0.55\pm 0.30\pm 0.22$	$0.13^{+0.06}_{-0.04}\pm 0.07$	$<0.01$	$0.68^{+0.31}_{-0.30}\pm 0.23$	0
$\ge 10$j, 2b	$\ge 400$	$1.98^{+0.69}_{-0.67}\pm 0.24$	$0.30^{+0.14}_{-0.10}\pm 0.12$	$0.05\pm 0.02\pm 0.02$	$2.33^{+0.70}_{-0.68}\pm 0.28$	0
$\ge 10$j, 3b	$\ge 400$	$0.77\pm 0.35\pm 0.09$	$0.00^{+0.45}_{-0.00}\pm 0.00$	$0.05\pm 0.03\pm 0.02$	$0.82^{+0.57}_{-0.35}\pm 0.09$	1
$\ge 10$j, $\ge 4$b	$\ge 400$	$0.09\pm 0.05\pm 0.01$	$0.00^{+0.45}_{-0.00}\pm 0.00$	$<0.01$	$0.09^{+0.45}_{-0.05}\pm 0.01$	0

### Search for disappearing tracks: search regions and yields

The 68 search regions defined for the disappearing track search, as described in Sect. 3.2.2, are summarized in Tables 24, 25 and 26, together with the pre-fit background predictions and the observed yields.

**Table 24:** Summary of the 28 signal regions of the search for disappearing tracks, for the 2016 data set, together with the corresponding background predictions and observations. For the background predictions, the first uncertainty listed is statistical (from the limited size of control samples), and the second is systematic. The systematic uncertainty is not shown when it is negligible

Track length	$N_{\mathrm {j}}$	$H_{\mathrm {T}}$ range ($\text {Ge}\text {V}$)	Track $p_{\mathrm {T}}$ ($\text {Ge}\text {V}$)	Label	Background	Data
P	2–3	[ 250, 450 )	[ 15, 50 )	P LL lo	$15.5^{+3.0}_{-2.7}\pm 3.2$	16
			[ 50, $\infty$ )	P LL hi	$9.8^{+2.6}_{-2.2}\pm 2.5$	3
		[ 450, 1200 )	[ 15, 50 )	P LM lo	$4.2^{+1.0}_{-0.9}\pm 1.2$	2
			[ 50, $\infty$ )	P LM hi	$2.02^{+0.66}_{-0.55}\pm 0.63$	1
		[ 1200, $\infty$ )	[ 15, 50 )	P LH lo	$0.19^{+0.26}_{-0.13}\pm 0.13$	0
			[ 50, $\infty$ )	P LH hi	$0.06^{+0.14}_{-0.05}\pm 0.03$	0
	$\ge 4$	[ 250, 450 )	[ 15, 50 )	P HL lo	$3.3^{+0.7}_{-0.6}\pm 1.4$	1
			[ 50, $\infty$ )	P HL hi	$1.98^{+0.43}_{-0.38}\pm 0.57$	1
		[ 450, 1200 )	[ 15, 50 )	P HM lo	$4.7^{+0.8}_{-0.7}\pm 1.9$	6
			[ 50, $\infty$ )	P HM hi	$2.37^{+0.50}_{-0.44}\pm 0.55$	1
		[ 1200, $\infty$ )	[ 15, 50 )	P HH lo	$0.43^{+0.24}_{-0.17}\pm 0.27$	0
			[ 50, $\infty$ )	P HH hi	$0.17^{+0.10}_{-0.07}\pm 0.04$	0
M	2–3	[ 250, 450 )	[ 15, 50 )	M LL lo	$3.9^{+1.5}_{-1.2}\pm 1.3$	3
			[ 50, $\infty$ )	M LL hi	$14^{+3.7}_{-3.2}\pm 4.0$	8
		[ 450, 1200 )	[ 15, 50 )	M LM lo	$2.1^{+0.89}_{-0.71}\pm 1.1$	3
			[ 50, $\infty$ )	M LM hi	$0.68^{+0.90}_{-0.45}\pm 0.35$	4
		[ 1200, $\infty$ )	[ 15, 50 )	M LH lo	$0.0^{+0.25}_{-0.0}\pm 0.0$	0
			[ 50, $\infty$ )	M LH hi	$0.0^{+0.7}_{-0.0}$	0
	$\ge 4$	[ 250, 450 )	[ 15, 50 )	M HL lo	$1.8^{+0.6}_{-0.5}\pm 0.9$	0
			[ 50, $\infty$ )	M HL hi	$2.1^{+0.8}_{-0.6}$ $^{+2.3}_{-2.1}$	2
		[ 450, 1200 )	[ 15, 50 )	M HM lo	$2.2^{+0.7}_{-0.6}\pm 1.3$	1
			[ 50, $\infty$ )	M HM hi	$2.9^{+0.9}_{-0.8}\pm 2.3$	0
		[ 1200, $\infty$ )	[ 15, 50 )	M HH lo	$0.23^{+0.23}_{-0.13}\pm 0.11$	0
			[ 50, $\infty$ )	M HH hi	$0.30^{+0.40}_{-0.20}\pm 0.29$	1
L	2–3	[ 250, 1200 )	[ 15, $\infty$ )	L LLM	$0.046^{+0.050}_{-0.034}$ $^{+0.057}_{-0.046}$	0
		[ 1200, $\infty$ )	[ 15, $\infty$ )	L LH	$0.015^{+0.036}_{-0.015}$ $^{+0.022}_{-0.015}$	0
	$\ge 4$	[ 250, 1200 )	[ 15, $\infty$ )	L HLM	$0.092^{+0.136}_{-0.085}$ $^{+0.130}_{-0.092}$	0
		[ 1200, $\infty$ )	[ 15, $\infty$ )	L HH	$0.0^{+0.1}_{-0.0}$	0

**Table 25:** Summary of the 24 signal regions of the search for disappearing tracks for pixel tracks, for the 2017–2018 data set, together with the corresponding background predictions and observations. For the background predictions, the first uncertainty listed is statistical (from the limited size of control samples), and the second is systematic. The systematic uncertainty is not shown when it is negligible

Track length	$N_{\mathrm {j}}$	$H_{\mathrm {T}}$ range ($\text {Ge}\text {V}$)	Track $p_{\mathrm {T}}$ ($\text {Ge}\text {V}$)	Label	Background	Data
P3	2–3	[ 250, 450 )	[ 15, 50 )	P3 LL lo	$78^{+9}_{-9}\pm 34$	73
			[ 50, $\infty$ )	P3 LL hi	$43.9^{+6.7}_{-6.2}\pm 8.1$	41
		[ 450, 1200 )	[ 15, 50 )	P3 LM lo	$30^{+5}_{-5}\pm 16$	21
			[ 50, $\infty$ )	P3 LM hi	$13^{+3}_{-3}\pm 13$	16
		[ 1200, $\infty$ )	[ 15, 50 )	P3 LH lo	$0.0^{+1.0}_{-0.0}$	1
			[ 50, $\infty$ )	P3 LH hi	$0.43^{+0.98}_{-0.36}\pm 0.34$	0
	$\ge 4$	[ 250, 450 )	[ 15, 50 )	P3 HL lo	$25.8^{+3.8}_{-3.4}\pm 7.9$	17
			[ 50, $\infty$ )	P3 HL hi	$10.8^{+2.1}_{-1.8}\pm 3.5$	7
		[ 450, 1200 )	[ 15, 50 )	P3 HM lo	$28.9^{+4.0}_{-3.7}\pm 5.7$	37
			[ 50, $\infty$ )	P3 HM hi	$12.3^{+2.2}_{-1.9}\pm 6.8$	11
		[ 1200, $\infty$ )	[ 15, 50 )	P3 HH lo	$3.1^{+1.5}_{-1.1}\pm 0.5$	5
			[ 50, $\infty$ )	P3 HH hi	$0.49^{+0.65}_{-0.32}\pm 0.12$	3
P4	2–3	[ 250, 450 )	[ 15, 50 )	P4 LL lo	$24^{+5}_{-5}\pm 11$	10
			[ 50, $\infty$ )	P4 LL hi	$4.1^{+1.9}_{-1.5}\pm 3.7$	0
		[ 450, 1200 )	[ 15, 50 )	P4 LM lo	$8.7^{+2.7}_{-2.2}\pm 4.6$	8
			[ 50, $\infty$ )	P4 LM hi	$1.1^{+0.7}_{-0.5}$ $^{+1.4}_{-1.1}$	0
		[ 1200, $\infty$ )	[ 15, 50 )	P4 LH lo	$0.40^{+0.91}_{-0.33}\pm 0.40$	0
			[ 50, $\infty$ )	P4 LH hi	$0.0^{+0.39}_{-0.0}$	0
	$\ge 4$	[ 250, 450 )	[ 15, 50 )	P4 HL lo	$6.3^{+1.6}_{-1.3}\pm 2.2$	7
			[ 50, $\infty$ )	P4 HL hi	$0.62^{+0.35}_{-0.25}\pm 0.43$	0
		[ 450, 1200 )	[ 15, 50 )	P4 HM lo	$6.9^{+1.6}_{-1.4}\pm 6.2$	2
			[ 50, $\infty$ )	P4 HM hi	$1.32^{+0.54}_{-0.43}\pm 0.63$	2
		[ 1200, $\infty$ )	[ 15, 50 )	P4 HH lo	$0.42^{+0.56}_{-0.28}\pm 0.12$	0
			[ 50, $\infty$ )	P4 HH hi	$0.08^{+0.18}_{-0.07}\pm 0.03$	0

**Table 26:** Summary of the 16 signal regions of the search for disappearing tracks for medium (M) length and long (L) tracks, for the 2017–2018 data set, together with the corresponding background predictions and observations. For the background predictions, the first uncertainty listed is statistical (from the limited size of control samples), and the second is systematic. The systematic uncertainty is not shown when it is negligible

Track length	$N_{\mathrm {j}}$	$H_{\mathrm {T}}$ range ($\text {Ge}\text {V}$)	Track $p_{\mathrm {T}}$ ($\text {Ge}\text {V}$)	Label	Background	Data
M	2–3	[ 250, 450 )	[ 15, 50 )	M LL lo	$8.4^{+2.4}_{-2.0}\pm 3.4$	8
			[ 50, $\infty$ )	M LL hi	$5.4^{+2.2}_{-1.8}\pm 2.6$	2
		[ 450, 1200 )	[ 15, 50 )	M LM lo	$1.90^{+0.85}_{-0.66}\pm 0.92$	6
			[ 50, $\infty$ )	M LM hi	$1.12^{+0.77}_{-0.54}\pm 0.97$	1
		[ 1200, $\infty$ )	[ 15, 50 )	M LH lo	$0.00^{+0.36}_{-0}$	0
			[ 50, $\infty$ )	M LH hi	$0.00^{+0.46}_{-0}$	0
	$\ge 4$	[ 250, 450 )	[ 15, 50 )	M HL lo	$1.6^{+0.6}_{-0.5}$ $^{+3.0}_{-1.6}$	3
			[ 50, $\infty$ )	M HL hi	$1.11^{+0.57}_{-0.42}\pm 0.58$	1
		[ 450, 1200 )	[ 15, 50 )	M HM lo	$1.9^{+0.6}_{-0.5}$ $^{+3.5}_{-1.9}$	3
			[ 50, $\infty$ )	M HM hi	$1.5^{+0.7}_{-0.5}\pm 1.1$	0
		[ 1200, $\infty$ )	[ 15, 50 )	M HH lo	$0.38^{+0.31}_{-0.19}$ $^{+0.70}_{-0.38}$	1
			[ 50, $\infty$ )	M HH hi	$0.12^{+0.29}_{-0.10}\pm 0.04$	0
L	2–3	[ 250, 1200 )	[ 15, $\infty$ )	L LLM	$0.46^{+0.26}_{-0.20}$ $^{+0.53}_{-0.46}$	0
		[ 1200, $\infty$ )	[ 15, $\infty$ )	L LH	$0.00^{+0.14}_{-0}$	0
	$\ge 4$	[ 250, 1200 )	[ 15, $\infty$ )	L HLM	$0.013^{+0.015}_{-0.014}$ $^{+0.018}_{-0.013}$	0
		[ 1200, $\infty$ )	[ 15, $\infty$ )	L HH	$0.000^{+0.008}_{-0}$	0

## Detailed results

### Inclusive $M_{\mathrm {T2}}$ search

Figures 23 and 24 show the background estimates and observed data yields in the regions $250< H_{\mathrm {T}} <450$, $450< H_{\mathrm {T}} <575$, $1200< H_{\mathrm {T}} <1500$, and $H_{\mathrm {T}} >1500$ $\,\,\text {Ge}\text {V}$. Each bin corresponds to a single $M_{\mathrm {T2}}$ bin, and vertical lines identify ($H_{\mathrm {T}}$, $N_{\mathrm {j}}$, $N_{{\text {b}}}$) topological regions.
